# Computational and Biological Evaluation of *β*-Adrenoreceptor Blockers as Promising Bacterial Anti-Virulence Agents

**DOI:** 10.3390/ph15020110

**Published:** 2022-01-18

**Authors:** Ahmad J. Almalki, Tarek S. Ibrahim, Sameh S. Elhady, Wael A. H. Hegazy, Khaled M. Darwish

**Affiliations:** 1 Department of Pharmaceutical Chemistry, Faculty of Pharmacy, King Abdulaziz University, Jeddah 21589, Saudi Arabia; tmabrahem@kau.edu.sa; 2 Department of Natural Products, Faculty of Pharmacy, King Abdulaziz University, Jeddah 21589, Saudi Arabia; ssahmed@kau.edu.sa; 3 Department of Microbiology and Immunology, Faculty of Pharmacy, Zagazig University, Zagazig 44519, Egypt; waelmhegazy@daad-alumni.de; 4 Department of Medicinal Chemistry, Faculty of Pharmacy, Suez Canal University, Ismailia 41522, Egypt

**Keywords:** bacterial virulence, *β*-adrenergic blockers, quorum sensing, adrenergic hormones, bacterial espionage, *Pseudomonas aeruginosa*, *Salmonella* *typhimurium*

## Abstract

Bacterial resistance to antibiotics is an increasing public health threat as it has the potential to affect people at any stage of life, as well as veterinary. Various approaches have been proposed to counteract the bacterial resistance development. Tackling bacterial virulence is one of the most promising approaches that confer several merits. The bacterial virulence is mainly regulated by a communication system known as quorum sensing (QS) system. Meanwhile, bacteria can sense the adrenergic hormones and eavesdrops on the host cells to establish their infection, adrenergic hormones were shown to enhance the bacterial virulence. In this study, *β*-adrenoreceptor blockers were proposed not only to stop bacterial espionage on our cells but also as inhibitors to the bacterial QS systems. In this context, a detailed in silico study has been conducted to evaluate the affinities of twenty-two *β*-blockers to compete on different structural QS receptors. Among the best docked and thermodynamically stable *β*-blockers; atenolol, esmolol, and metoprolol were subjected to further in vitro and in vivo investigation to evaluate their anti-QS activities against *Chromobacterium violaceum*, *Pseudomonas aeruginosa* and *Salmonella* *typhimurium*. The three tested *β*-blockers decreased the production of QS-controlled *C. violaceum*, and the formation of biofilm by *P. aeruginosa* and *S. typhimurium*. Additionally, the tested *β*-blockers down-regulated the *P. aeruginosa* QS-encoding genes and *S.* *typhimurium* sensor kinase encoding genes. Furthermore, metoprolol protected mice against *P. aeruginosa* and *S.* *typhimurium*. Conclusively, these investigated *β*-blockers are promising anti-virulence agents antagonizing adrenergic hormones induced virulence, preventing bacterial espionage, and blocking bacterial QS systems.

## 1. Introduction

Bacterial infections are among major burdens which constitute a serious challenge. Despite the marked achievements in discovering new antimicrobial agents over the last seven decades [1], the development of antimicrobial resistance fades these achievements. In the battle against bacterial infections, bacteria showed a magnificent capability in developing resistance to all antibiotic classes [2]. In this context, the development of new strategies to overcome the bacterial resistance is mandatory, one of the effective strategies, is quenching bacterial virulence [3,4,5]. Bacteria utilize several kinds of virulence factors that expand from bacterial structures as capsules, flagella, pili to producing an arsenal of enzymes like protease, urease, elastase, and others [6]. The bacterial community utilizes specific chemical language to orchestrate its virulence in coordination with the surrounding circumstances. This chemical system is called quorum sensing (QS), in which bacteria produce chemical signals that find their specific receptors to arrange the virulence behavior [7]. QS system controls biofilm formation, bacterial motility, production of virulence enzymes and other virulence factors as extensively reviewed [8,9]. Both Gram-positive and Gram-negative bacteria use QS. Gram-positive QS systems alter the expression of virulence genes using sensor kinase receptors and cytoplasmic transcription factors to sense oligopeptides [8]. On the other hand, Gram-negative bacteria often use numerous autoinducers (AIs) that are mainly acyl-homoserine lactones (AHLs) [10]. These AHLs are able to diffuse freely through the bacterial membrane to be detected by the QS receptors which are mainly LuxR-type receptors [9,10]. Then, LuxR-AHLs complexes regulate the expression of virulence genes via binding lux boxes which are short DNA sequences located upstream of targeted genes [11].

The communication between bacteria and their eukaryotic host cells is essential to determine the outcome of infections. Bacteria use membrane sensors to detect the changes and facilitate their adaptation in the surrounding environment inside host cells [12]. Meanwhile, AIs are employed in bacterial communication via QS to orchestrate the bacterial virulence, AIs crosstalk with the host neuroendocrine hormones (NE) adrenaline and noradrenaline for activation of the same signaling pathway [13]. Recently, there is cumulative evidence to propose that Gram-negative bacteria sense and respond to the host NE stress hormones to enhance virulence as reviewed [12,14]. As a result, hindering the adrenergic receptor inhibits the bacterial receptor-based sensing and diminishes the bacterial virulence [12,13,14]. That leads us to hypothesize that adrenergic blockers may be a considerable candidate to diminish the bacterial espionage and hence mitigate the bacterial virulence.

**Figure 1:**
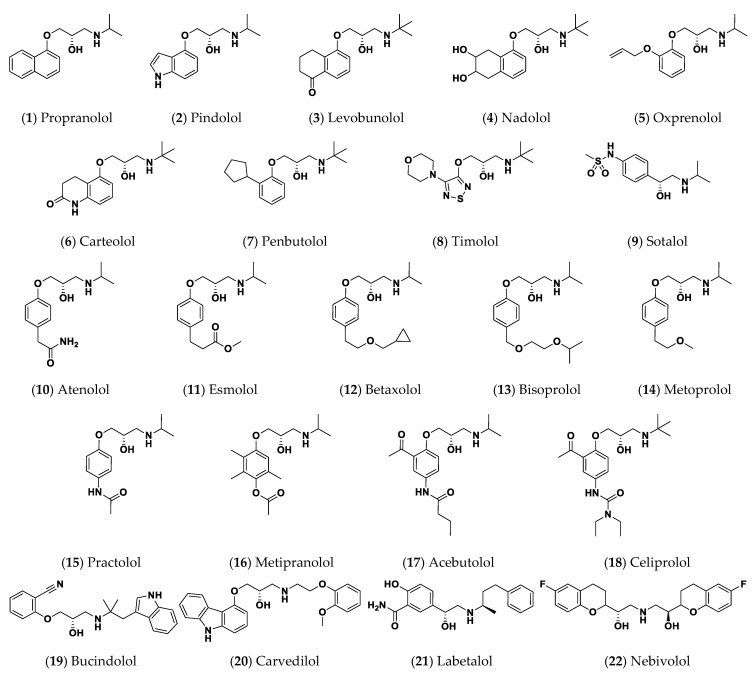
Two-dimensional chemical structural representation of investigated adrenoreceptor antagonists (*β*-blockers).

Bacteria evoke all these interplayed mechanisms to escape from the immunity, causing more aggressive complications [3,4,6]. In this direction, targeting bacterial virulence is a highly appreciated strategy. There are several advantages, this strategy does not affect bacterial growth and so it will not induce bacterial resistance [2,3,4] and will also not destroy the bacterial normal flora [9,15]. Moreover, the diminishing of bacterial virulence gives the chance to the immune system to be activated efficiently against weakened bacteria [16]. Dozens of studies were directed to screen and evaluate the use of diverse chemical moieties as anti-virulence agents, but among all the tested compounds, approved safe drugs gained an increasing interest [3,4,17,18,19]. Drug repurposing is exploring new uses of already known drugs which are famed by specific medical use. Repurposing of already approved drugs is an attractive strategy where it comprises several advantages. Saving costs and time is one of the merits of this strategy [20]. The *β*-blockers are widely used with diverse pharmacological applications as reviewed [21]. The *β*-blockers comprise diverse chemical structures (Figure 1), but all share the presence of an aromatic ring linked to a side alkyl chain including an amine and hydroxyl functional groups [21]. The *β*-blockers possess at least one chiral center in their structures indicating that their interaction with various receptors is highly stereoselective [21,22], supposing the increasing possibility to block bacterial QS receptors.

In this study, we aimed to investigate the inhibitory activities of adrenergic *β*-blockers on the Gram-negative bacteria virulence. We in silico studied the binding ability of *β*-blockers to the well resolved full-length three different LuxR-type QS receptors; TraR from *Agrobacterium tumefaciens*, CviR from *Chromobacterium violaceum*, and QscR from *Pseudomonas aeruginosa* [10]. The promising in silico behaved *β*-blockers were then subjected to further in vitro and in vivo investigations against different Gram-negative bacterial models, including *C. violaceum*, *P. aeruginosa* and *Salmonella typhimurium*.

## 2. Results

### 2.1. Two-Stage Multi-Target Docking Analysis

The docking affinity of 22 FDA-approved adrenoreceptor (*β*)-blockers was evaluated on three LuxR-type quorum sensing transcription factors (QSs) from *A. tumefaciens* (TraR; PDB entry: 1L3L) [23], *P. aeruginosa* (homolog QscR; PDB entry: 3SZT) [24], and *C. violaceum* (CviR; PDB entry: 3QP5) [25]. Molecular docking is a rapid in silico technique for grasping the orientational and conformational degrees of freedom for several small ligands within the target protein pocket at cost-wise computational expenditures. Scoring and ranking ligands on bases of docking affinity scoring functions allow investigators to prioritize these small molecules for further acquisition and investigation [26]. Based on parameterized force-fields calculations, this in silico technique allows scoring the ligand–protein interactions as a function of Kcal/mol binding energies where more negative values represent higher energy of the ligand–protein complex in relation to each individual entity [27]. Using controls is usually employed to enhance the accuracy of docking predictions for increasing the reliability of simulating the molecular flexibility within calculating the binding energies close to experimental values [28].

Within this presented manuscript, the adopted docking workflow involved two stages, where the first stage was a rapid preliminary screening docking protocol for selecting the significant leads in relation to LuxR-type QSs reference co-crystalline ligands. The second stage is a more sophisticated docking protocol which aimed to further validate the previously obtained docking findings, besides obtaining more valid docking binding modes for comprehensive investigation of predicted ligand–protein binding interactions. Preliminary docking analysis illustrated higher docking binding energies for the CviR’s co-crystalline inhibitor (chlorolactone; HLC) as compared to other two co-crystalline ligand controls at their respective biological targets (TraR and QscR autoinducer pheromones; N-3-oxo-octanoyl-_L_-homoserine lactone (O-C8-HSL) and O-C12-HSL, respectively). The co-crystalline inhibitor (HLC) revealed docking energies of −6.6245 Kcal/mol, −7.6488 Kcal/mol, and −7.2051 Kcal/mol for TraR *A. tumefaciens*, QscR *P. aeruginosa*, and CviR *C. violaceum*, respectively. These reference binding energies were set as the threshold for selecting promising hits in term of more negative values. Out of the 22 investigated *β*-blockers, only six compounds on TraR *A. tumefaciens*, seven compounds QscR *P. aeruginosa*, and eight ligands on CviR *C. violaceum* showed significant docking energies better than those of their respective references (Table 1). Interestingly, three promising ligands; atenolol (10), esmolol (11), and metoprolol (14) were found satisfactory for the three investigated bacterial LuxR-type quorum sensing transcription factors. These obtained hit compounds were considered as relevant hits that would worth further investigation.

As a general observation, docking energies across all investigated ligands were at lower negative values at TraR Agrobacterium tumefaciens as compared to the other two biological targets. This was significantly obvious for the large-sized ligands including the third generation *β*-blockers (20, 21, and 22). To further investigate the differential pocket size across the three targets, the on-line Computed Atlas of Surface Topography of proteins server (CASTp; http://sts.bioe.uic.edu/castp/index.html, accessed on 17 September 2021) was used with 1.4 Å probe and at default settings to estimate the pocket area/volume across the three QSs proteins [29]. The calculated Richard’s solvent accessible surface area andvolume were estimated as; 562.41 Å^2^/315.97 Å^3^, 579.64 Å^2^/331.18 Å^3^, and 516.30 Å^2^/363.28 Å^3^ for the binding sites of TraR *A. tumefaciens*, QscR *P. aeruginosa*, and CviR *C. violaceum*, respectively (Supplementary Data, Figure S1). Additionally, the CASTp pocket analysis further illustrated the differential topology between CviR *C. violaceum* and QscR *P. aeruginosa* pockets where the earlier is quite wider, while as QscR was depicted narrower and more elongated. Comprehensive investigation of ligand–protein interaction for the obtained hits was proceeded through the second stage docking protocol.

#### 2.1.1. Binding Interaction Analysis of Ligand–TraR *A. tumefaciens* Complexes

**Figure 2:**
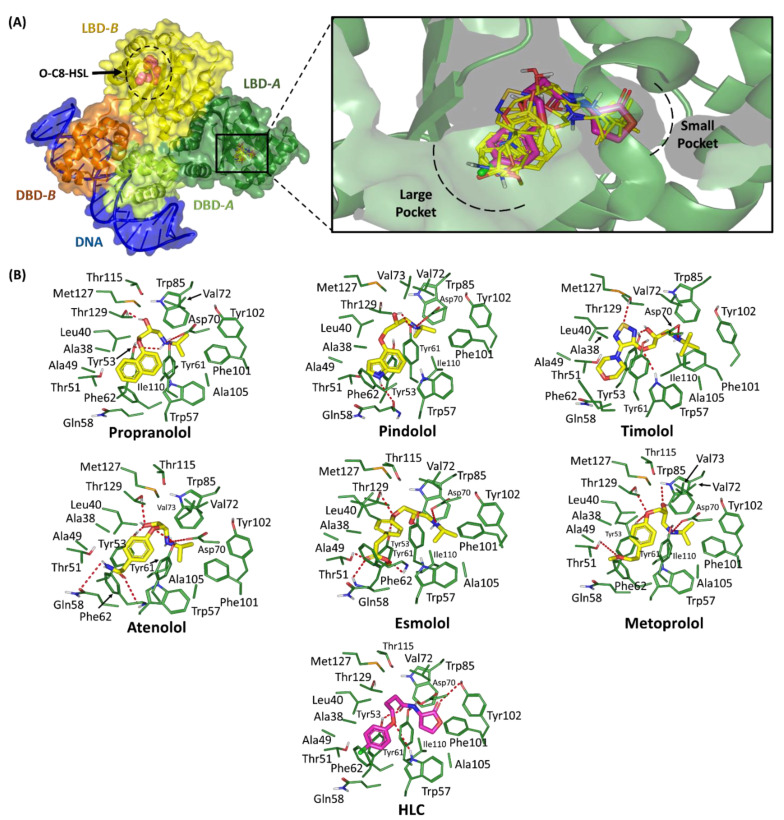
Ligand–protein binding interactions. (**A**) Three-dimensional cartoon and surface representation of TraR *A. tumefaciens* bounded to DNA sites (PDB entry: 1L3L), where each protomer is colored differently in regard to its ligand binding domain (LBD) and DNA binding domain (DBD) as light/dark green and dark/light orange for protomer-A and -B, respectively, whereas DNA is presented as the blue cartoon. The co-crystalline ligand, O-C8-HSL, is presented as magenta spheres at the LBD of protomer A (LBD-A; yellow). Showing at LBD of protomer B (LBD-B; green) an overlay of investigated compounds (yellow lines) and HLC (magenta sticks) binding to the protein’s canonical binding site comprising of large hydrophobic and small sub-pockets; (**B**) Predicted binding modes of the docked ligands (yellow sticks). Only residues located within 5Å radius of bound ligands are displayed as green lines as being colored according to their subsite location (green for LBD), and finally labeled with sequence number. Non-polar hydrogens are removed for clarity. Hydrogen bonding is depicted as red dashed lines.

The directed flexible docking protocol of the six promising TraR *A. tumefaciens* hits illustrated a general common conformation/orientation for these ligands within the target’s pocket. The alkylated nitrogen moieties of the investigated ligands were settled at the small sub-pocket having their nitrogen atoms at significant superimposition with the amide group of the reference control ligand, HLC (Figure 2). On the other hand, the ligands’ terminal aromatic (phenolic or heterocyclic) scaffolds were perfectly anchored at the pocket’s large hydrophobic site at similar orientation as that of the HLC’s non-polar aromatic terminal functionality. Polar interaction with the anionic charged residue, Asp90, was conserved across all docked propanolamine-based adrenoreceptor hits owing to their respective alkylated nitrogen atom being quaternary under physiological conditions (Table 2). Almost all ligands depicted strong hydrogen bond interactions with the sidechains of Tyr53 and/or Thr126 as being mediated via the ligands’ free hydroxyl group or oxygen linker of the ligands’ respective aryloxypropanolamine scaffold (Supplementary Data, Table S1). On the other hand, extra hydrogen bond pairings were predicted for the Trp57 sidechain ε N-atom with compound **8**, Tyr61 sidechain hydroxyl group with compounds **2** and **10**, as well as Trp85 sidechain NH with only compound **14**. A similar polar interaction pattern was assigned for the reference inhibitor, HLC, where its lactone head and double carbonyl groups mediated hydrogen bonding with Tyr53, Trp57, and Asp70, as well as the small sub-pocket vicinal residue Tyr102.

Concerning the ligand accommodation at the large hydrophobic site, it was noticed that ligands’ terminal aryl scaffolds were significant for mediating extra stability of the investigated hits at the TraR target pocket. Generally, comparable hydrophobic contacts with several pocket lining residues including Ala38, Leu40, Ala49, Tyr53, Trp57, Phe62, and Ile110, were depicted for all investigated hits. The latter was related to the TraR’s pocket tightness bringing several hydrophobic amino acids at close proximity towards the ligand’s terminal aryl groups. Further stability of these aromatic moieties was mediated through π-π interaction with the Tyr61 sidechain where the latter was settled at relevant orientation at the ligands’ aromatic rings. Only compound **8** depicted π-H interaction between the Tyr61 sidechain and ligand’s terminal morpholine ring since the central 1,2,5-thiadiazole ring adopted quite a far orientation in relation to the Tyr61 aromatic sidechain.

#### 2.1.2. Binding Interaction Analysis of Ligand–QscR *P. aeruginosa* Complexes

**Figure 3:**
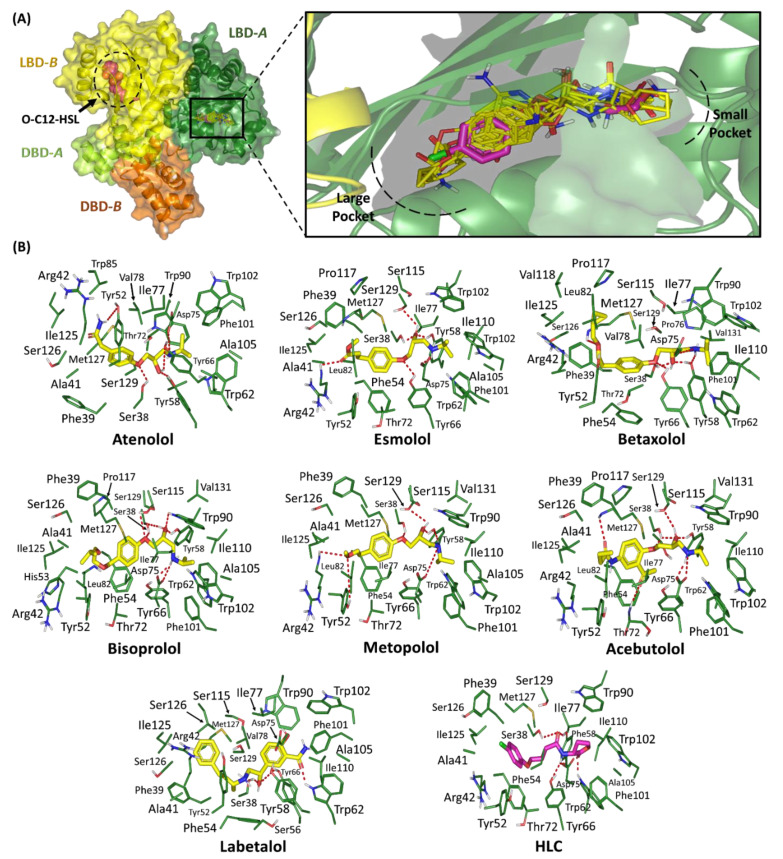
Ligand–protein binding interactions. (**A**) Three-dimensional cartoon and surface representation of QscR *P. aeruginosa* bounded to DNA sites (PDB entry: 3SZT), where each protomer is colored differently in regard to its ligand binding domain (LBD) and DNA binding domain (DBD) as light/dark green and dark/light orange for protomer-A and -B, respectively. The co-crystalline ligand, O-C12-HSL, is presented as magenta spheres at the LBD of protomer A (LBD-A; yellow). Showing at LBD of protomer B (LBD-B; green) an overlay of investigated compounds (yellow lines) and HLC (magenta sticks) binding to the protein’s canonical binding site comprising of large hydrophobic and small sub-pockets; (**B**) Predicted binding modes of the docked ligands (yellow sticks). Only residues located within 5 Å radius of bound ligands are displayed as green lines as being colored according to their subsite location (green for LBD), and finally labeled with sequence number. Non-polar hydrogens are removed for clarity. Hydrogen bonding is depicted as red dashed lines.

The seven preliminary hits, obtained from the first docking-based screening stage, illustrated favorable accommodations owing to the relevant contacts depicted for these compounds with important residues of the target’s pocket. Common conformation/orientation positions were also illustrated for these investigated adrenoreceptor blockers (Figure 3). The ligand’s aromatic hydrophobic features were settled at the large-sized hydrophobic sub-pocket offering minimal steric hinderance for these large scaffolds. Concerning the other end of the ligands’ structures, significant docking poses at the target’s small sub-pocket were assigned for the ligand’s *N*-alkylated nitrogen heads. The predicted ligand target poses illustrated great superimposition for the ligand’s ionizable heads with the lactone ring of QscR’s co-crystalline ligand, O-C12-HSL, while as their respective aromatic scaffold being directed towards the large hydrophobic sub-pocket. The docked ligands at the QscR *P. aeruginosa* binding site exhibited a more extended conformation as compared to those within the TraR *A. tumefaciens* pockets. This was obvious where the *β*-blocker drug class members predicted almost linear conformation for their aliphatic spacer (propanolamine scaffold) extending their terminal aryl and alkylated nitrogen moieties at the far ends of the QscR sub-pockets. 

Several key pocket residues were depicted important for anchoring the docked ligands within the QscR pocket. Polar interactions with the sidechain of the anionic Asp75 were illustrated for almost all docked propanolamine-based adrenoreceptor hits (Table 3). Showing proximity towards the ligand’s quaternary nitrogen atom, the Asp75-mediated polar binding interaction was suggested significant for anchoring the ligand at the QscR’s small sub-pocket. Notably, the large-sized ligands, such as compound **21**, lacked relevant interaction with this charged Asp75 amino acid owing to their respective bulkiness and extended orientations within the QscR pocket. The latter compounds depicted docking poses that disfavored the close proximity of the ligands’ alkylated nitrogen scaffold near the Asp75 sidechain. Further stabilization of the docked adrenoreceptor hits was mediated through a wide range of polar residues such as Ser38, Arg42, Tyr52, Tyr58, Trp62, Tyr66, Thr72, Trp90, Met127, and/or Ser129 (Supplementary Data, Table S2).

Besides the ligand binding polar interactions, the docket hits also depicted relevant van der Waals hydrophobic interactions with QscR non-polar residues including; Phe39, Ala41, Tyr52, His53, Tyr58, Trp62, Ile77, Val78, Leu82, Trp90, Phe101, Trp102, Ile110, Pro117, Ile125, Met127, and/or Val131. Extended π-mediated hydrophobic interactions were also depicted as significant for stabilizing the ligand/QscR complexes, particularly through π–π interaction with Phe54 as well as CH-π contacts with Tyr58, Tyr66, Trp90, and/or Trp102. Additional, hydrophobic van der Waals binding with Arg42 sidechain hydrocarbons were also depicted for almost all QscR-docked hits and reference ligand. Comparable residue-wise binding profile was illustrated for the reference potent QscR inhibitor, HLC, where its amidic lactone scaffold mediated several polar contacts with Ser38, Tyr58, Trp62, Tyr66, and Asp75, besides the significant hydrophobic contacts with Phe58 and Trp90 via the ligand’s aromatic lipophilic tail.

#### 2.1.3. Binding Interaction Analysis of Ligand–CviR *C. violaceum* Complexes

**Figure 4:**
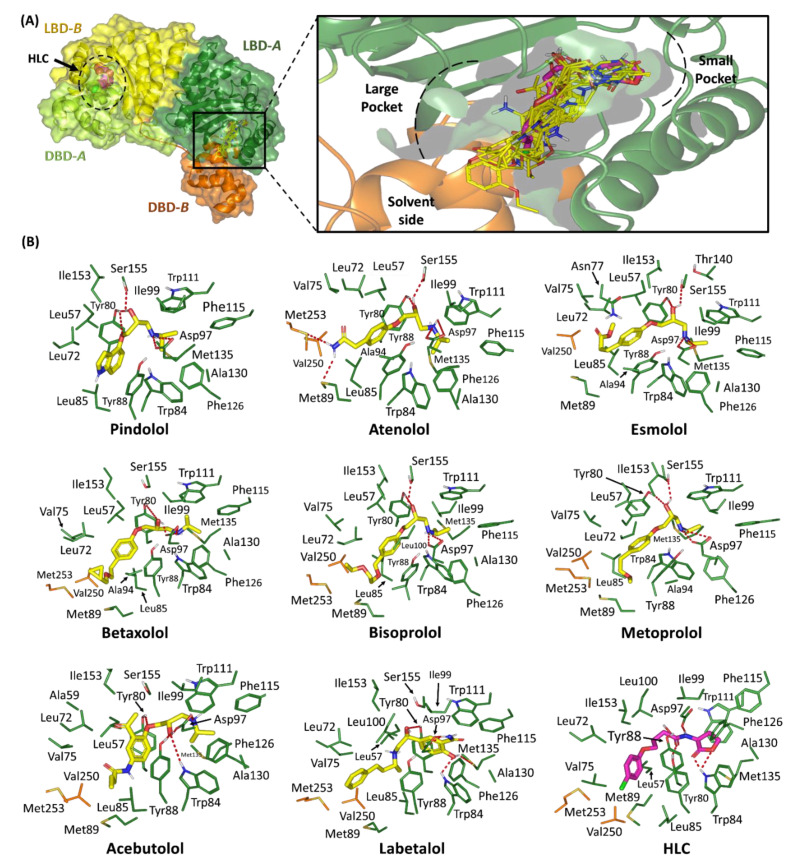
Ligand–protein binding interactions. (**A**) Three-dimensional cartoon and surface representation of CviR *C. violaceum* bounded to DNA sites (PDB entry: 3QP5), where each protomer is colored differently in regard to its ligand binding domain (LBD) and DNA binding domain (DBD) as light/dark green and dark/light orange for protomer-A and -B, respectively. The co-crystalline ligand, HLC, is presented as magenta spheres at the LBD of protomer A (LBD-A; yellow). Showing at LBD of protomer B (LBD-B; green) an overlay of investigated compounds (yellow lines) and HLC (magenta sticks) binding to the protein’s canonical binding site comprising of large hydrophobic and small sub-pockets; (**B**) Predicted binding modes of the docked ligands (yellow sticks). Only residues located within 5Å radius of bound ligands are displayed as lines, colored according to their subsite location (green for LBD and orange for DBD), and finally labeled with sequence number. Non-polar hydrogens are removed for clarity. Hydrogen bonding is depicted as red dashed lines.

Results of the directed induced-fitting docking protocol at the CviR *C. violaceum* canonical binding site revealed favorable anchoring of the eight preliminary hits. Common conformation/orientation positions were illustrated for these investigated adrenoreceptor blockers showing great superimposition with the comparable pharmacophoric features of the crystalline ligand, HLC (Figure 4). The ligand’s aromatic hydrophobic scaffolds were oriented towards the large-sized hydrophobic sub-pocket within comparable orientation to the HLC’s chlorinated phenyl ring. Such orientation offered minimal steric hinderance for these large scaffolds at the depicted pocket. The *N*-alkylated nitrogen heads of the investigated were directed into the target’s small sub-pocket depicting great superimposition with the lactone ring of CviR’s co-crystalline ligand, HLC. The docked ligands at the CviR *C. violaceum* binding site exhibited a more extended conformation as compared to those within the TraR *A. tumefaciens*. On the other hand, ligands were in a curved conformation with respect to their *N*-alkylated nitrogen heads where these polar scaffolds depicted deep anchoring into the target’s small sub-pocket. That is why, the docked compounds showed inverted L-shaped conformations within the CviR *C. violaceum* canonical binding site. This was significantly different from the almost linear conformations adopted by same ligands at the QscR active site.

Stability of the docked ligands within the CviR *C. violaceum* active site were assigned to several pocket’s lining residues (Table 4). The catalytic Asp97 residues illustrated consistent polar interactions with the quaternary nitrogen atoms of the investigated *β*-blockers being at close distance for optimum hydrogen bonding with the negatively charged amino acid. The ligand–Asp97 polar pairing was suggested significant for ligand’s anchoring at the CviR’s small sub-pocket. Notably, all docked adrenoreceptor blockers predicted multiple polar interactions with the Asp97 sidechain. In addition to the polar interaction with the ligand’s quaternary nitrogen atom, the oxygen atoms of the latter residue’s sidechain served as significant hydrogen bond acceptors. The latter polar functionalities depicted significant hydrogen bond pairing with the ligand’s NH-head as well as were able to even interact with some ligand’s free hydroxyl group at their respective propanolamine linkers. Further stabilization of the docked adrenoreceptor hits at the CviR *C. violaceum* canonical binding site was mediated through extended hydrogen bond networks with wide range of pocket’s polar residues including Tyr80, Trp84, Tyr88, Met89, Ser155, and/or Met253. 

Besides the ligand-binding polar interactions, the docket hits depicted almost conserved van der Waals/hydrophobic contacts with CviR non-polar residues including; Leu57, Leu72, Val75, Trp84, Leu85, Tyr88, Met89, Ala94, Ile99, Leu100, Phe115, Phe126, Ala130, Met135, Ile153, Val250, and/or Met253. Extended π-mediated hydrophobic interactions were also depicted significant for stabilizing the ligand/CviR complexes. These significant non-polar interactions were mediated through π–π interaction with several aromatic pocket’s residues, particularly Tyr80, and/or Tyr8, being settled at close proximity from the ligands’ hydrophobic moieties (Supplementary Data, Table S3). Moreover, relevant close-range CH–π interactions between the docked compounds and the sidechains of Tyr88 or Trp111 were also depicted. Unlike TraR and QscR–ligand complexes, no additional ligand-directed hydrophobic van der Waals binding were predicted with the sidechain hydrocarbons of pocket’s polar residues lining the large hydrophobic sub-pocket. Comparable residue-wise binding profile was illustrated for the crystalline and reference potent CviR inhibitor, HLC, where its amidic lactone scaffold mediated polar interactions Tyr80, Trp84, and Asp97, in addition to significant π-mediated hydrophobic contacts with Tyr80, Tyr88, and Trp111 via the ligand’s aromatic scaffold.

### 2.2. Molecular Dynamics Simulation

For gaining insights regarding the ligand/protein thermodynamic behavior while accounting for solvent effect (solvation energies/Gibbs free energy changes) on the ligand–protein interaction., the molecular dynamics simulation is an effective computational tool [30]. This approach was adopted within the presented study to validate the potential affinity of the investigated adrenoreceptor inhibitor hits against the QS biological targets within a near physiological conditions [31]. 

#### 2.2.1. Analysis of Ligand–TraR *A. tumefaciens* Complexes

**Figure 5:**
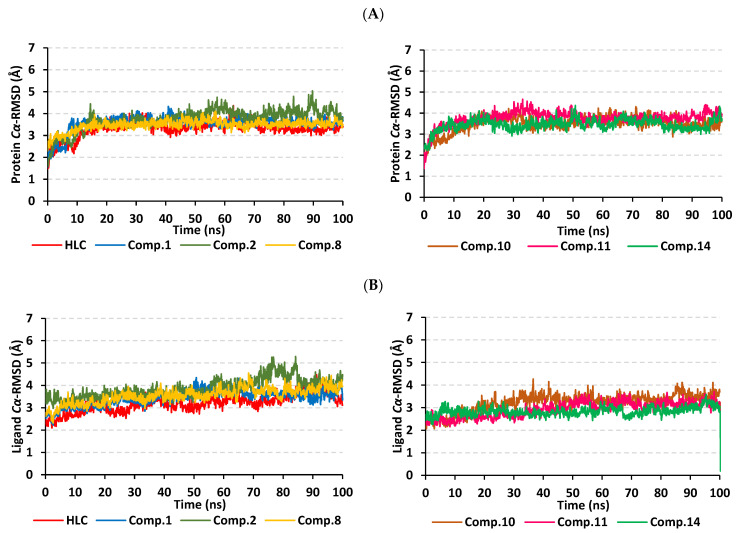
Stability analysis of generated *Cα*-RMSD trajectories for investigated compounds and reference inhibitor in complex with TraR *A. tumefaciens* protein along 100 ns all-atom MD simulation. (**A**) protein *Cα*-RMSD; (**B**) sole ligand *Cα*-RMSD trajectories (Å), both in reference to the protein alpha-carbon atoms of the initial frame, across MD simulation time (ns).

The root-mean-square deviation (RMSD) trajectory analysis was adopted for investigating the thermodynamic nature of the ligand–protein complex as well as the individual protein and ligand identities. Generally, RMSD estimates the molecular deviation of a particular ligand relative to a designated original/reference structure. Such an analytical tool would provide a good indication for the ligand–target stability and the adopted MD simulation protocol was valid. Target’s instability and significant conformational alterations are associated with high RMSD trajectories [32]. On the other hand, high RMSDs would correlate to a limited ligand–target affinity where the ligand is unable to be confined within the target’s canonical binding site along the simulation periods [33]. Herein, the estimated RMSD deviations for the TraR *A. tumefaciens* proteins, in reference to their respective alpha-carbon (*Cα*-RMSD), depicted an overall typical behavior for molecular dynamics (MD) simulations (Figure 5A). Over the initial frames, the protein’s *Cα*-RMSD tones increase as a result of constraining release at the beginning of MD simulation runs. Following the first 20 ns, steady protein’s *Cα*-RMSD trajectories were obtained for more than half of the simulation runs (>50 ns) except for minimal fluctuation for the compound **2**-bound protein around 60 and 90 ns timeframes. Notably, almost all investigated proteins leveled-off at comparable *Cα*-RMSD trajectories across the trajectory plateau and till the end of MD simulation courses. Comparable *Cα*-RMSD tones were obtained for the HLC as well as compounds **10**, **11**, and **14**-bound TraR proteins following their respective equilibration with average values of 3.31 ± 0.36 Å, 3.48 ± 0.38 Å, 3.46 ± 0.35 Å, and 3.44 ± 0.28 Å, respectively. However, slightly higher values were assigned for compound **2**-bound protein (3.87 ± 0.48 Å) being correlated to its depicted limited fluctuations as well as quite late equilibration following the 30 ns of the MD simulation timeframes. The compound **14**-bound TraR protein managed to exhibit the steadiest *Cα*-RMSD tones with the lowest standard deviation value after the equilibration being attained.

To investigate the ligand’s confinement within the TraR *A. tumefaciens* binding site, the sole ligand’s *Cα*-RMSDs relative to the reference protein backbone frame were monitored along the whole MD timeframe. Despite limited fluctuations, all examined compounds and reference ligand managed to illustrate backbone *Cα*-RMSD plateau reflecting their significant confinement within the target pocket (Figure 5B). Steady tones were depicted for all ligands with averages ranging from 3.02 ± 0.42 Å to 3.85 ± 0.43 Å. However, only compound **2** showed relevant fluctuations around the 80 ns MD simulation time, yet it managed to converge at comparable *Cα*-RMSD like the other investigated ligands at the end of the MD run (100 ns).

Conformational analysis of the ligand–protein models across the MD simulation timeframe was performed through examining the ligand–protein models at trajectories of the start and final timeframes. Frames at 0 ns and 100 ns for each ligand–protein model were extracted and minimized to a 0.001 kcal/mol·A^2^ gradient using the MOE system preparation package. Notably, stable binding profiles were assigned for all simulated *β*-blockers as well as the reference ligand (Supplementary Data, Figure S2). Ligands showed favored orientation/conformation at both sub-pockets of the TraR binding site, while as limited ligand orientation alterations were depicted at the end of the MD simulation runs.

**Figure 6:**
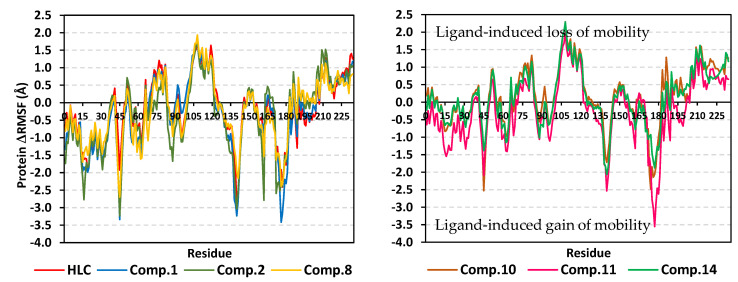
Analysis of ΔRMSF trajectories versus residue number for TraR *A. tumefaciens* protein, in complex with the investigated *β*-blockers and reference ligand, throughout the whole 100 ns MD simulation window. The ΔRMSF values, in reference to protein backbone atoms, were estimated considering independent MD simulation of TraR apo/unliganded state against the holo ones where the latter were complexed with the investigated ligands or crystalline reference inhibitor, HLC. The ΔRMSF trajectories are represented as a function of residue number of the whole bounded protomer.

Further investigation of the local protein flexibility and how this could be contributed to the ligand–protein binding, the RMS-fluctuations (RMSF) stability analysis was performed. Generally, the RMSF provides a valuable evaluation of the target’s residues dynamic behavior represented as both fluctuation and flexibility, through estimating the average deviation of each protein’s amino acid in relation to its respective reference position across time [34]. Thus, monitoring the fluctuation of QS’s residues by estimating the RMSF stability validation parameter for each protein residue would highlight the residue-wise contribution within the target protein stability. The fluctuation of TraR’s residues was monitored by estimating the difference root-mean-square fluctuation (ΔRMSF = apo RMSF—holo RMSF) as a stability validation parameter. Investigating the RMSF trajectories essentially execute for a trajectory region considered stable. Since the TraR protein targets were of significant conformational stability along the 100 ns MD simulations for all systems, despite some limited fluctuations, the *Cα*-RMSF calculations were done across the whole MD simulation trajectories. Applying the cut-off mobility threshold at ΔRMSF of 0.30 Å, lower fluctuation patterns were depicted for the TraR residues of each ligand–protein complex at the carboxy end in relation to those located near the *N*-terminus (average 0.82 ± 0.23 Å versus −0.62 ± 0.68 Å) (Figure 6). Beside the *C*-terminal, the residue ranges along 70–80 and 100–120 showed the highest immobility profiles with ΔRMSF up to 1.32 and 2.00 Å, respectively. On the contrary, residues around 40–65, 55–70, 125–150, and 160–180 ranges were of the most flexible pattern (ΔRMSF down to the highest negative values~−3.50 Å). Trends of more positive/less negative ΔRMSF values were assigned for compounds **8**, **10**, **11**, and **14**-bound protein residues relative to those of the other *β*-blockers or even the reference ligand inhibitor. This was recognized across several ranges of protein residues being most noticeable for the flexible 1–40 and 50–85 residue ranges near the *N*-terminus. Comparative analysis of the furnished ΔRMSF trajectories was proceeded regarding the specific flexibility of the pocket’s key lining residues. Interestingly, several canonical pocket residues, as well as vicinal residues, showed significant ΔRMSF values above the cut-off mobility threshold 0.30 Å (Supplementary Data, Table S4). Compound **10** showed the widest range of pocket’s residue for immobility as compared to other ligands. On the other hand, compounds **8** and **14** were with pocket residue-associated inflexibility profile being comparable to that of the reference inhibitor, HLC. Pocket’s residues including; Thr51, Tyr53, Val72, Phe101, Tyr102, Ala105, Ile110, Thr115, as well as the catalytic Asp70 showed the most recognized inflexibility profiles (ΔRMSF up to 1.69 Å) being consistent across several simulated ligand–TraR models. 

The Molecular Mechanics/Poisson–Boltzmann Surface Area (MM/PBSA) calculation of the binding free energy was performed to further understand the nature of the ligand–protein interaction, explore the comparative ligand binding site affinity, and obtain more information concerning the individual ligand/residue contributions [35]. The MM/PBSA is considered of comparable accuracy to the Free Energy Perturbation approaches, yet with much smaller computational expenses [36]. The SASA-only model of the binding free energy calculation (Δ*G*_Total_ = Δ*G*_Molecular Mechanics_ + Δ*G*_Polar_ + Δ*G*_Apolar_), as well as the single trajectory approach, were adopted and the higher negative binding energy explains more ligand affinity towards its respective target pocket. The MM/PBSA approach estimates binding free energy as a contribution of several energy terms through these given Equations [36]:

Δ*G*_binding_ = *G*_complex_ − (*G*_ligand_ + *G*_protein_)

*Gx* = (*E*_Molecular Mechanics_) − *TS* + *G*_solvation_

*E*_Molecular Mechanics_ = *E*_bonded_ + (*E*_vdW_ + *E*_electrostatic_)

*G*_solvation_ = *G*_polar_ + *G*_Apolar_

*G*_Apolar_*= γSASA* + *b*

where, Δ*G*_binding_ is the binding free energy correlating to ligand–protein binding where the higher negative energy values infer greater protein–ligand affinity where the *G*_complex_ is of higher Gibbs free energy than the individual entity (ligand/protein). The Gibbs energy terms of each entity (*Gx*) are respective total free energies of ligand−protein complex, isolated protein, and isolated ligand in solvent, where x is the protein or ligand or protein–ligand complex. Vacuum molecular mechanics potential energy (*E*_Molecular Mechanics_) together with entropic contribution to free energy (*TS*) and free energy of solvation (*G*_solvation_) provided the total free energy of the protein, ligand, or ligand−protein complex (*Gx*). The *E*_bonded_ is the bonded interactions comprising the angle, bond, improper interactions, and torsions. The non-bonded interactions (*E*_non-bonded_) include both van der Waals (*E*_vdw_) and electrostatic (*E*_elec_) interactions being modeled via Lennard-Jones’s and Coulomb’s potentials, respectively. Terms *T* and *S* denote temperature and entropy, respectively, while *G*_Molecular Mechanics_ was calculated based on the molecular mechanics force-field parameters. The *G*_solvation_ energy form comprises polar (electrostatic) and non-polar (non-electrostatic) parts contributing to the solvation free energy, where the latter was estimated using the most widely used non-polar model (SASA-only non-polar model). Within this model, the SASA and *b* terms represent solvent accessible surface area and fitting constant, respectively. Finally, *G*_polar_ was solved from the Poisson–Boltzmann equation.

To our delight, all of the investigated *β*-blockers depicted significant free binding and affinity towards the target’s pocket (Table 5). The latter binding free energy pattern came in great concordance with the preliminary docking investigation showing preferential higher docking scores for the hit *β*-blockers in relation to the positive control inhibitor, HLC. Interestingly, the highest comparable total binding free energies (Δ*G*_Total binding_ at significant negative values) were furnished for the simulated compounds **1**, **11**, and **14** (−84.32 ± 0.66, −89.71 ± 3.56, and −89.05 ± 4.71 kJ/mol, respectively). Compounds **2** and **10** showed moderate free-binding energies, while as both compound **8** and reference ligand depicted nearly comparable ligand–target binding affinities. Dissecting the obtained binding free energy into its contributing energy terms showed a dominant energy contribution for the van der Waal interactions (Δ*G*_van der Waals_) within the binding free energy calculation with the highest values being assigned for compounds **10**, **11**, and **14**. On the other hand, the electrostatic energy contributions (Δ*G*_Electrostatic_) were significantly higher in compounds **2**, **10**, and **11**. Finally, lower polar solvation energies (Δ*G*_Solvation; Polar_) were illustrtaed for compound **1** and **14**, as well as the reference control inhibitor, whereas the apolar solvation energy (Δ*G*_Solvation; SASA_) was almost comparable for all ligands. 

**Figure 7:**
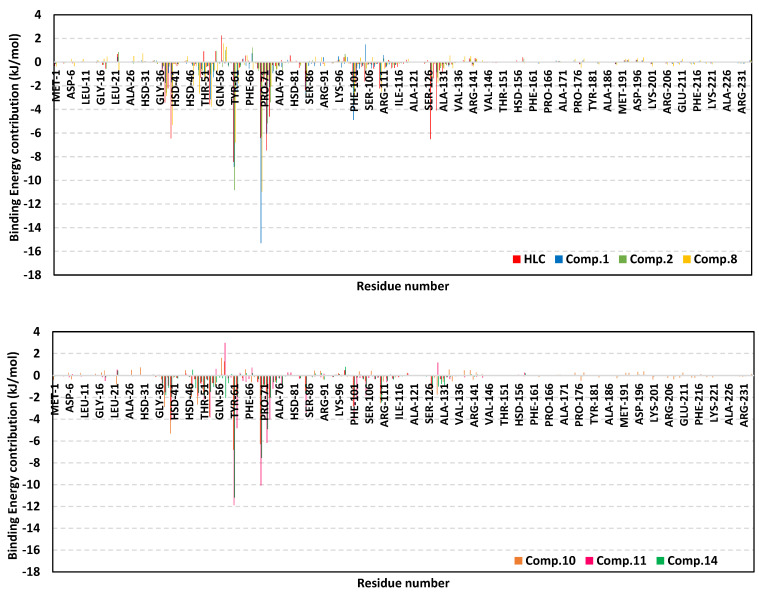
Binding free energy/residue decomposition illustrating the protein residue contribution at ligand–TraR *A. tumefaciens* complex Δ*G*_Total binding_ calculation. The binding energy contributions are represented as a function of residue number of the whole bounded protomer.

For gaining more insights regarding ligand–residue interactions, the binding free energy decomposition within the *g_mmpbsa* module was utilized to identify the key residues involved within the obtained binding free energies [36]. Significant residues that exhibited favored contribution (high negative values) within the ligand–protein binding energy were those comprising the TraR binding site as well as their vicinal residues (Figure 7). Residues including Tyr53, Tyr61, Asp70, Val72, exhibited the most favored free energy contributions (>−5.00 kJ/mol) across the simulated ligand–TraR models. On the other hand, Trp57 pocket residue showed positive energy contribution with all ligands inferring a repulsion effect and an unfavored role in ligand–pocket binding. It worth noting that this latter pocket residue (Trp75) depicted significant flexibility and instability (down to −0.64 Å) at the above described ΔRMSF analysis.

#### 2.2.2. Analysis of Ligand–QscR *P. aeruginosa* Complexes

**Figure 8:**
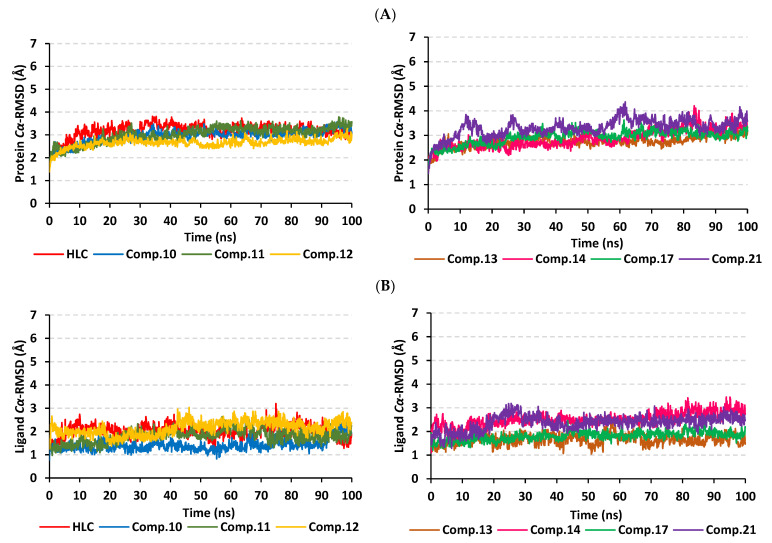
Stability analysis of generated *Cα*-RMSD trajectories for investigated compounds and reference inhibitor in complex with QscR *P. aeruginosa* protein along 100 ns all-atom MD simulation. (**A**) protein *Cα*-RMSD; (**B**) sole ligand *Cα*-RMSD trajectories (Å), both in reference to the protein alpha-carbon atoms of the initial frame, across MD simulation time (ns).

The typical MD thermodynamic behavior of the simulated QscR proteins was illustrated through monitoring the protein’s *Cα*-RMSD trajectories. The QscR protein managed to attain early equilibration/convergence state for more than half the MD simulation run (Figure 8A). Only compound **17**-bound QscR protein showed limited fluctuations around 60 ns and at the far end of the MD simulation timeframe. Nevertheless, the latter fluctuations were not exceeding an *Cα*-RMSD values of 0.5 Å above those of the other proteins. Comparable findings were illustrated with compound **14**-bound protein yet much limited RMSD tone fluctuations were depicted. Average protein’s *Cα*-RMSD values were the lowest for compound **10** and **14**-bound proteins (2.76 ± 0.23 Å and 2.88 ± 0.37 Å, respectively), while as being the highest for HLC and compound **21**-bound QscR proteins (3.17 ± 0.30 Å and 3.31 ± 0.36 Å, respectively).

Regarding the ligand’s *Cα*-RMSD and its confinement within the target pocket, an overall steady trajectory pattern was observed for all ligands (Figure 8B). Managing to achieve a *Cα*-RMSD plateau with average RMSD trajectories ranging from 1.52 ± 0.25 Å to 2.82 ± 0.29 Å, with only just highest RMSD values being assigned for compound **14**. The latter high ligand’s *Cα*-RMSD tones were never exceeding 1.5 Å of any of the simulated ligands across the whole MD simulation time frame. Notably, the comparative RMSD values for the same ligands across different QSs showed relatively lower values at the QscR *P. aeruginosa* pocket in relation to the TraR *A. tumefaciens* binding site. Ligand’s orientation stability within the QscR active site was illustrated through the conformational analysis of the ligand–protein models across the MD simulation timeframes 0 ns and 100 ns (Supplementary Data, Figure S3). Limited orientation/conformation changes were illustrated across the MD simulation run being even at more profound stability profiles as compared to those for the same ligands at the TraR binding site.

**Figure 9:**
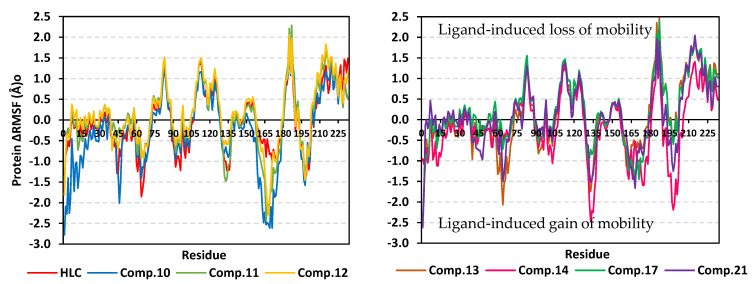
Analysis of ΔRMSF trajectories versus residue number for QscR *P. aeruginosa* protein in the complex with the investigated *β*-blockers and reference ligand, throughout the whole 100 ns MD simulation window. The ΔRMSF values, in reference to protein backbone atoms, were estimated considering independent MD simulation of QscR apo/unliganded state against the holo ones where the latter were complexed with the investigated ligands or crystalline reference inhibitor, HLC. The ΔRMSF trajectories are represented as a function of residue number of the whole bounded protomer.

Comparable residue-wise flexibility modes were depicted for the MD simulated QscR protein targets in regard to TraR ones. Throughout the ΔRMSF analysis and along the 100 ns MD run, higher immobility profiles were assigned for the residue’s region being vicinal to the carboxy end rather than those at the *N*-terminus (average 0.82 ± 0.23 Å versus −0.62 ± 0.68 Å) (Figure 9). Corresponding residue ranges at the core region of the simulated QscR proteins showed comparable flexibility/inflexibility profiles as those at TraR proteins. That is why residue ranges along 75–90 and 110–130 showed the significantly high immobility profiles with ΔRMSF up to 1.50 Å. On similar comparable bases to TraR proteins, residues across the 40–60, 65–70, 130–140, and 160–180 ranges were of the most flexible patterns (ΔRMSF down to the highest negative values~−2.50 Å). Unlike the TraR targets, the simulated QscR proteins depicted an extra stabilized residue region (185–195) near the carboxy terminus showing the highest immobility profile (ΔRMSF = 2.50 Å). Trends of less negative ΔRMSF values were assigned for the amino acids at proteins in complex with compounds **11**, **12**, **14**, and **17** relative to those of the other *β*-blockers or even the reference ligand inhibitor. This was recognized across several ranges of protein residues being most noticeable for the flexible regions across 1–70 residue range at the ligand-binding domain. Specific flexibility of the pocket’s key lining residues in relation to bounded ligand showed interesting findings (Supplementary Data, Table S5). Compounds **11**, **12**, and **17** showed the widest residue range of immobility, whereas the rest of investigated ligands depicted nearly comparable pocket residue-associated inflexibility profile in relation to HLC. Pocket’s residues including; Arg42, Ile77, Val78, Leu82, Trp90, Ile125, Met127, and Ser129 showed the most recognized inflexibility profiles (ΔRMSF up to 1.02 Å). The latter three amino acids (Ile125, Met127, and Ser129) showed consistent stability across all simulated ligand–TraR models. Important pocket–ligand binding residues such as Ser38 and the catalytic Asp75 amino acids were depicted significantly immobile at proteins in complex with compounds **10**–**13** and/or HLC.

**Figure 10:**
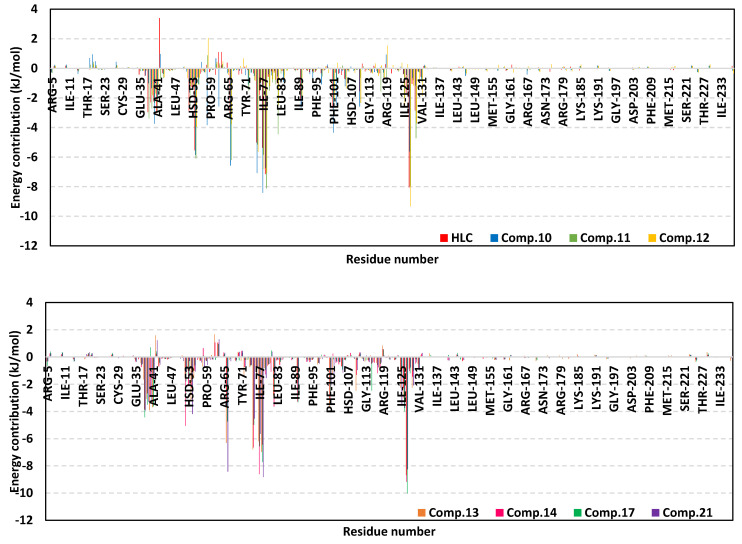
Binding free energy/residue decomposition illustrating the protein residue contribution at ligand–QscR *P. aeruginosa* complex Δ*G*_Total binding_ calculation. The binding energy contributions are represented as a function of residue number of the whole bounded protomer.

The MM/PBSA calculation of the ligand–QscR complexes’ binding free energy showed higher negative values and better binding affinity for the simulated *β*-blockers as compared to reference inhibitor, HLC (Table 6). Among the investigated *β*-blocker agents, compounds **10**, **11**, and **14** showed the highest comparable total binding free energies (−117.63 ± 6.12, −118.47 ± 13.07, and −114.40 ± 13.87 kJ/mol, respectively). Compounds **12**, **13**, and **21** showed moderate binding free energies (−93.00 ± 9.32 to −109.04 ± 2.77 kJ/mol), where as the acyl substituted *β*-blocker (compound **17**) was of the lowest ligand–target binding affinities among all investigated drug class members. It worth noting that similar ligands depicted higher binding free energies at QscR as compared to TraR complexes. Dissecting the obtained ligand–QscR binding free energy into its contributing energy terms showed a dominant energy contribution of the van der Waal interactions. Compounds **11**, **12**, **14**, and **21** showed the highest hydrophobic energy contribution terms. On the other hand, the electrostatic energy contributions were significantly higher in compounds **10** and **11**. Finally, lower polar solvation energies (Δ*G*_Solvation; Polar_) were assigned for compound **13**, as well as the reference control inhibitor, whereas the Δ*G*_Solvation; SASA_ was almost comparable for all lignads. Similar to TraR findings, the residues that showed favored contribution (high negative values) within the ligand–protein binding energy were those comprising the active binding site as well as their vicinal residues (Figure 10). Pocket’s residues including Phe54, Asp73, Ile77, Val78, and Met127, exhibited the most favored free energy contributions (>−5.00 kJ/mol) across the simulated ligands. Moderate energy contributions (around −3.00 kJ/mol) were assigned for Ser38, Tyr66, Trp90, and Phe101 amino acids. On the contrary, Tyr58, and Lys63 pocket residue showed positive energy contribution with few simulated ligands (HLC, compounds **10**, **11**, **12**, **13**, **14**, and/or 21) inferring repulsion effect and unfavored role in their respective ligand–pocket binding.

#### 2.2.3. Analysis of Ligand–CviR *C. violaceum* Complexes

**Figure 11:**
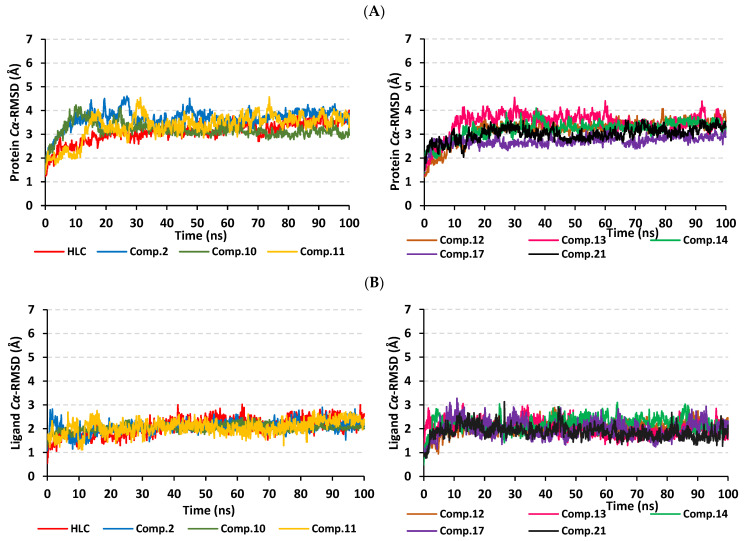
Stability analysis of generated *Cα*-RMSD trajectories for investigated compounds and reference inhibitor in complex with CviR *C. violaceum* protein along 100 ns all-atom MD simulation. (**A**) protein *Cα*-RMSD; (**B**) sole ligand *Cα*-RMSD trajectories (Å), both in reference to the protein alpha-carbon atoms of the initial frame, across MD simulation time (ns).

Monitoring the protein’s *Cα*-RMSD tones illustrated typical MD behavior and efficient convergence for the simulated CviR proteins. These proteins managed to attain valid equilibration/convergence state beyond the initial 30 ns and for more than half the MD simulation run (Figure 11A). Limited initial fluctuations were depicted for compounds **2**, **11**, and **13**, before they rapidly attain their equilibration plateau. These latter fluctuations were not exceeding an *Cα*-RMSD values of 0.5 Å above those of other proteins where they all converge within close tones (~3.50 Å) at the end of the MD simulation timeframe. Average protein’s *Cα*-RMSD were of the lowest values for compounds **10** and 17-bound proteins (2.72 ± 0.20 Å and 3.07 ± 0.26 Å, respectively), while their was the highest for compound **2**-bound CviR protein (3.66 ± 0.38 Å). Beyond the initial 30 ns MD run, the steadiest protein’s *Cα*-RMSD tones were assigned for the proteins bounded to compounds **10**, **14**, and **17** as well as reference inhibitor, HLC.

Moving towards the ligand’s *Cα*-RMSD and its confinement within the target pocket, an overall steady trajectory pattern was observed for all ligands (Figure 11B). Managing to achieve *Cα*-RMSD plateau, the average RMSDs trajectories ranged from 1.87 ± 0.28 Å to 2.09 ± 0.36 Å, being only at the highest RMSD values for compound **14**. The latter high ligand’s *Cα*-RMSD tones were never exceeding 1.5 Å of any of the simulated ligands across the whole MD simulation timeframes. Interestingly, the comparative RMSD values for the same ligands across different QSs showed relatively lower values at the CviR *C. violaceum* pocket in relation to the TraR *A. tumefaciens* binding site yet being almost comparable to QscR *P. aeruginosa*. Ligand’s orientation stability within the CviR active site was illustrated through the conformational analysis of the ligand–protein models across the MD simulation timeframe at frames 0 ns and 100 ns (Supplementary Data, Figure S4). Limited orientation/conformation changes were illustrated across the MD simulation run being at more profound stability profiles as compared to those at the TraR binding site, while as of slightly lower stability profiles as compared to those at QscR.

**Figure 12:**
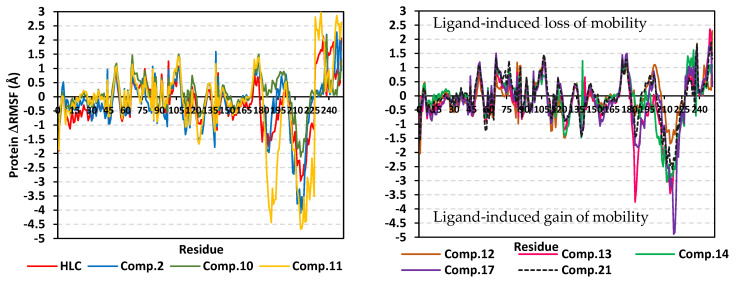
Analysis of ΔRMSF trajectories versus residue number for CviR *C. violaceum* protein in complex with the investigated *β*-blockers and reference ligand, throughout the whole 100 ns MD simulation window. The ΔRMSF values, in reference to protein backbone atoms, were estimated considering independent M D simulation of CviR apo/unliganded state against the holo ones where the latter were complexed with the investigated ligands or crystalline reference inhibitor, HLC. The ΔRMSF trajectories are represented as a function of residue number of the whole bounded protomer.

The ΔRMSF analysis along the 100 ns MD run showed interesting findings. Like the above two QS proteins, significant immobility profiles were assigned for the C-terminus residues as compared to those at the amine end of the proteins (average 0.88 ± 0.87 versus −0.69 ± 0.63 Å) (Figure 12). However, the stability pattern at carboxy terminus is less profound as compared to both TraR *A. tumefaciens* and QscR *P. aeruginosa*. Residue ranges along 65–80 and 95–115 showed a high inflexibility pattern with ΔRMSF up to 1.50 Å which is somewhat comparable to the simulated TraR and QscR core protein regions. Moreover, residues around 80–90, 135–140, 150–165, 180–195, and 210–225 ranges were of the most flexible pattern (ΔRMSF down to the highest negative values~−5.00 Å). Unlike the previously described QS proteins, the simulated CviR proteins depicted an extra stabilized residue region (45–55) near the *N*-terminus showing a significant immobility profile (ΔRMSF~1.20 Å). Compounds **2**, **11**, **13**, and **17** depicted high negative ΔRMSF values across the flexible regions; 180–195 and 210–225 residue range. Nevertheless, almost all compounds showed comparable ΔRMSF-determined immobility trends across the several regions at the ligand-binding domain. Specific flexibility of the pocket’s key lining residues in relation to bounded ligand was illustrated in Supplementary Data, Table S6. Notably, compounds **10**, **14**, and **17** showed the widest residue range of inflexibility as compared to other ligands, whereas the rest of the investigated ligands depicted nearly a comparable pocket residue-associated inflexibility profile in relation to HLC. Pocket’s residues including; Leu57, Tyr80, Trp84, Tyr88, Asp79, Trp111, Ile153, Ser155, and Val250 showed the most recognized immobility profiles (ΔRMSF up to 2.37 Å). Four amino acids (Leu57, Tyr80, Trp111, and Ile153) showed consistent stability across all simulated ligand–CviR models. Key pocket–ligand-binding residues including Leu85, Met89, Phe115, and the catalytic Asp97 amino acids were depicted significantly immobile at proteins in complex with compounds **10**, **13**, **14**, **17**, **21**, and/or HLC.

**Figure 13:**
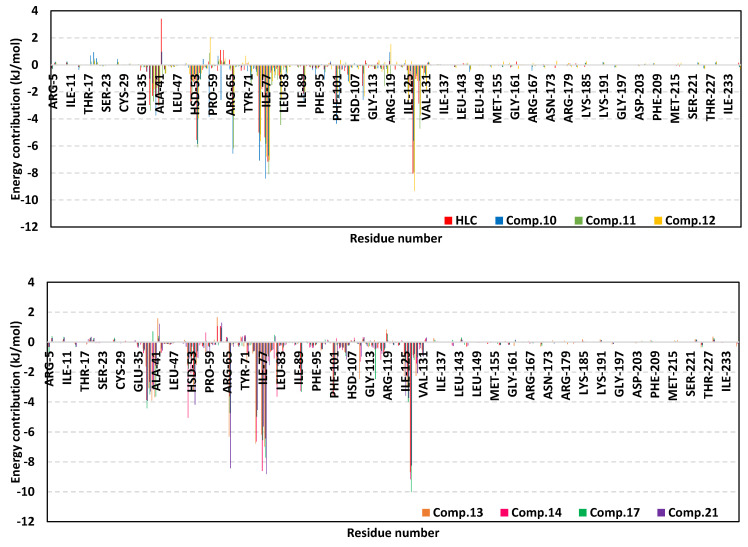
Binding free energy/residue decomposition illustrating the protein residue contribution at ligand–CviR *C. violaceum* complex Δ*G*_Total binding_ calculation. The binding energy contributions are represented as a function of residue number of the whole bounded protomer.

The MM/PBSA calculation of the ligand–CviR complexes’ binding free energy showed higher negative values and better binding affinity for the simulated *β*-blockers as compared to reference inhibitor, HLC (Table 7). Among the investigated *β*-blocker agents, compounds **10**, **11**, and **17** showed the highest comparable total binding free energies (−111.07 ± 6.04, −104.64 ± 4.79, and −101.46 ± 4.40 kJ/mol, respectively). Compounds **13** and **14** showed moderate binding free energies (−85.66 ± 4.18 and −98.51 ± 13.26 kJ/mol, respectively), whereas compounds **2**, **12**, and **21**, were of comparable ligand–target binding affinities. It worth noting that similar ligands depicted moderate binding free energies at CviR being in between the top affinities at QscR and the lower ones at TraR complexes. Dissecting the obtained ligand–CviR binding free energy into its contributing energy terms showed a dominant energy contribution of the van der Waal interactions within the binding free energy calculation of both the investigated and reference ligands. The highest van def Waal values were for compounds **11**, **14**, and **17**. On the other hand, the electrostatic energy contributions were significantly higher in compounds **10** and **11**. Finally, lower polar solvation energies (Δ*G*_Solvation; Polar_) were assigned for compound **12**, as well as the reference control inhibitor, whereas the Δ*G*_Solvation; SASA_ was almost comparable for all ligands. In similar fashion to both previously described QS complexes, residues of the active binding site showed favored contribution (high negative values) within the ligand–protein binding energies (Figure 13). Depicting high binding energy contribution (>−5.00 kJ/mol), pocket’s residues including Leu57, Tyr88, Asp97, Ile99, and Leu100, were considered significant for ligand–protein complex stability. The highest residue-wise energy contribution was assigned for Tyr88 pocket residue being up to −12.33 kJ/mol and −13.13 for compound **11** and **14**, respectively. Moderate energy contributions (around −3.00 kJ/mol) were assigned for Leu72, Val75, Leu85, Met89, Phe126, Ile153, and Ser155 amino acids. Nevertheless, both Arg71 and Tyr80 pockets residue showed positive energy contribution with HLC and compound **12** (for Tyr80) as well as almost all simulated ligands (for Arg71). The latter infers a repulsion effect and an unfavored impact on the stability of respective ligand–target complexes.

### 2.3. Determination of Selected B-Blockers’ Minimum Inhibitory Concentrations (MICs) against P. aeruginosa, C. violaceum and S. typhimurium

In order to attest the anti-virulence and anti-QS activities of promising *β*-blockers, the virtually identified hits with relevant docking energies, significant thermodynamic stability, and QS-directed binding affinity across MD simulation, were selected to be further investigated. Atenolol (10), esmolol (11), and metoprolol (14) were selected to test their anti-virulence activities against *P. aeruginosa, C. violaceum,* and *S. typhimurium*. Atenolol, esmolol, and metoprolol inhibited the growth of *P. aeruginosa* at concentrations (4, 4 and 2 mg/mL, respectively), *C. violaceum* (2, 2 and 1 mg/mL, respectively), and *S. typhimurium* at 2 mg/mL.

**Figure 14:**
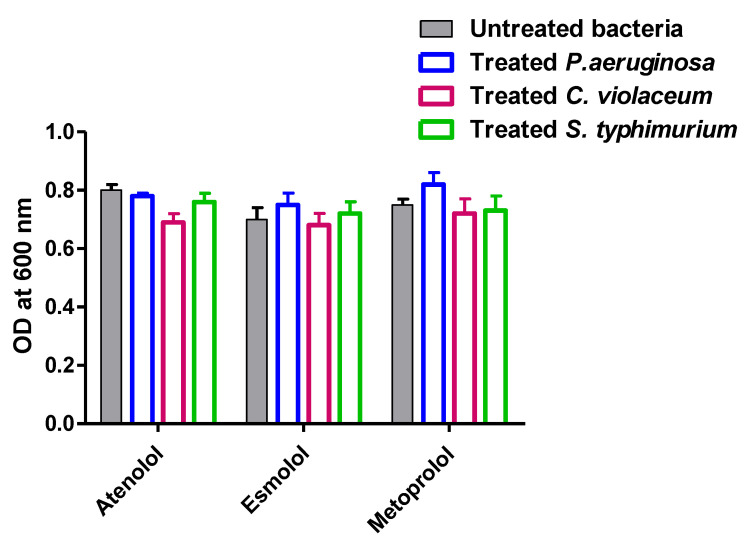
Effect of *β*-blockers on bacterial growth. The optical density of bacterial growth was measured at OD600 after overnight incubation in the absence and presence of 1/4 MIC of tested drugs. The test was done in triplicates. Two-way ANOVA test followed by Bonferroni post-test was used, *p* value < 0.05 was considered statistically significant. There were no statistically significant effects on bacterial growth.

The potential inhibition of virulence and QS by tested *β*-blockers may be owed due to their effects on bacterial growth. To exclude such possibility, bacterial overnight cultures in (Luria-Bertani) LB broth with and without tested drugs at their sub-MIC (1/4 MIC) were prepared and the turbidities of the bacterial suspensions were measured at 600 nm. There were no significant differences between the turbidities of bacterial growth in presence or absence of tested drugs at sub-MIC (Figure 14). All the subsequent assays were performed using tested *β*-blockers at sub-MIC (1/4 MIC). The experiment was performed in triplicate and the data were presented as means ± standard errors. Two-way ANOVA test followed by the Bonferroni post-test was employed to calculate the significance, *p* values < 0.05 were considered statistically significant.

### 2.4. Inhibition of Violacein Production

**Figure 15:**
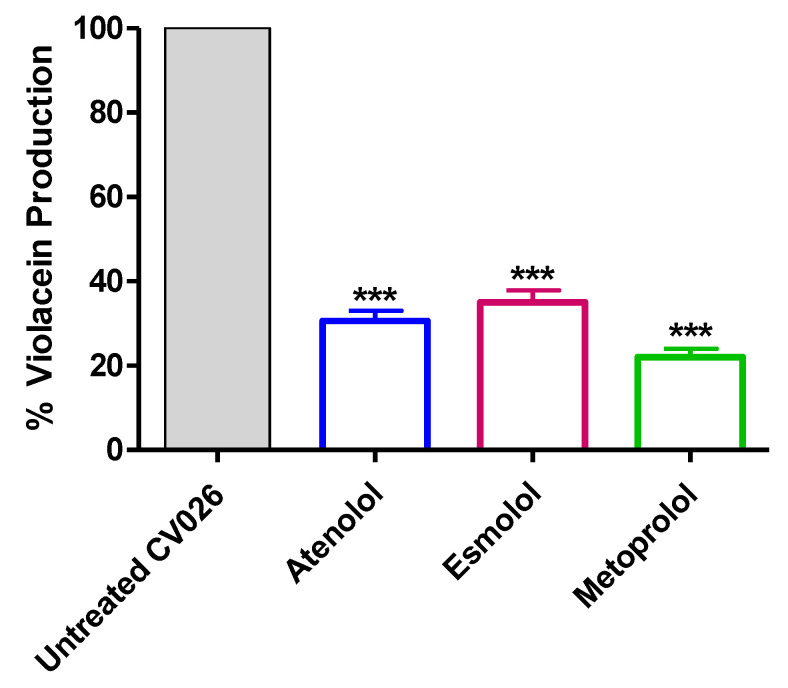
Effect of *β*-blockers on violacein pigment production. The absorbances of extracted violacein from the cells incubated in the presence of tested drugs (1/4 MIC) were measured and calculated as percentage change from untreated control. The test was done in triplicates and one-way ANOVA test followed by Tukey’s post-test was applied to determine the significance; significance was considered when *p* < 0.05. The three tested drugs significantly reduced the production of violacein (*** = *p* < 0.0001).

The biosensor *C. violaceum* CV026 is usually employed to assess the QS due to its ability to release the pigment violacein in response to acyl-homoserine lactones under the CVi/R QS system control [37]. To attest the ability of tested *β*-blockers to inhibit the QS systems, the production of QS-controlled violacein dye was quantified in the presence and absence of tested drugs at sub-MIC. The experiment was performed in triplicates and one-way ANOVA test followed by the Tukey’s post-test was used to calculate the significance. The results were presented as percentage change from untreated bacterial control as means ± standard errors. The three tested drugs significantly reduced the production of violacein at their sub-MIC (*p* < 0.0001) (Figure 15).

### 2.5. Anti-Biofilm Activities of β-Blockers in P. aeruginosa and S. typhimurium 

**Figure 16:**
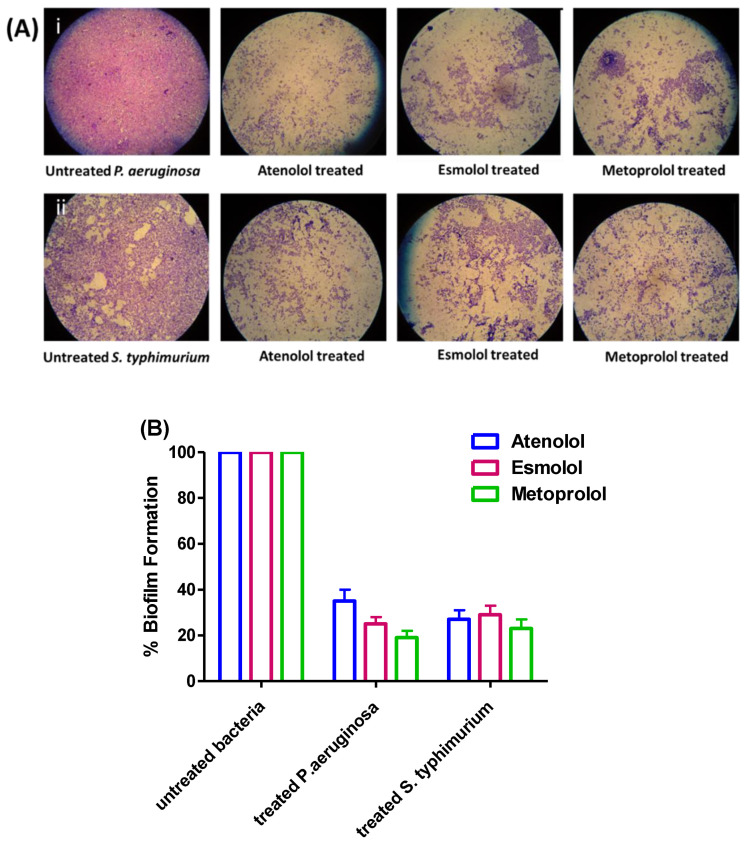
The Inhibitory effects of *β*-blockers against the biofilm formation in *P. aeruginosa* and *S. typhimurium*. (**A**) Representative light microscope images: the formed biofilms on sterile glass cover slips in the absence and presence of metoprolol at sub-MIC were stained with crystal violet and examined under the light microscope. Tested drugs treated (**i**) *P. aeruginosa* and (**ii**) *S. typhimurium* samples showed much fewer scattered cells in comparison to untreated cells. (**B**) The absorbances of crystal violet staining biofilm forming cells were measured. The data are presented as means ± standard errors of percentage changes from untreated bacterial cells. Two-way ANOVA test followed by the Bonferroni post-test was employed and significance was considered when *p* < 0.05. Three tested drugs significantly reduced the biofilm formation (*p* < 0.001).

To investigate the anti-biofilm effects of tested drugs, the absorbances of the extracted crystal violet that stained the adhered biofilm forming cells in the presence or absence of tested drugs at their sub-MICs were measured. The assay was repeated in triplicates and a two-way ANOVA test followed by the Bonferroni post-test was used to attest the significance. The data were presented as percentage change from untreated bacterial control as means ± standard errors. The three tested drugs at sub-MIC significantly reduced the formation of biofilm by *P. aeruginosa* and *S. typhimurium* (*p* < 0.001) (Figure 16).

### 2.6. Effect of β-Blockers on the Expression of Virulence and QS-Encoding Genes in P. aeruginosa and S. typhimurium

**Figure 17:**
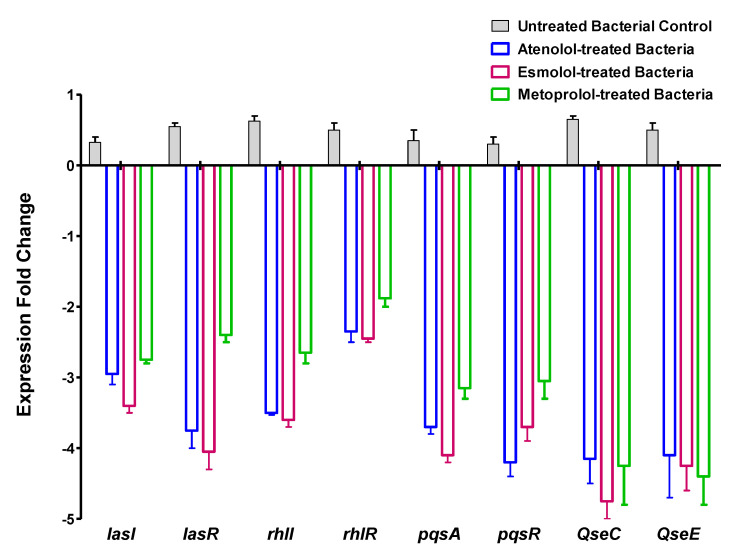
*β*-blockers down-regulate the QS-encoding genes in *P. aeruginosa* and adrenergic sensor kinases encoding genes in *S. typhimurium*. The expressions of tested genes in the presence of *β*-blockers were compared to untreated bacterial cells, and the data shown are the means ± standard errors. The test was done in triplicate and a two-way ANOVA test followed by the Bonferroni post-test was used to test the significance as *p* < 0.05 was considered significant. The tested drugs at sub-MIC significantly decreased the expression of all tested genes (*p* < 0.001).

To investigate the effect of tested *β*-blockers on the expression levels of QS-encoding genes of *P. aeruginosa* and bacterial adrenergic sensor kinases encoding genes in *S. typhimurium* qRT-PCR was performed (Figure 17). The expressions of tested genes were assessed in *P. aeruginosa* or *S*. *typhimurium* treated with tested drugs at sub-MIC using 2^−∆∆Ct^ method. The test was done in triplicates and a two-way ANOVA test followed by the Bonferroni post-test was used to test the significance (where *p* < 0.05 was considered significant). A significant decrease in the expression levels of *P. aeruginosa* QS-regulating genes *rhlI*, *rhlR, lasI, lasR*, *pqsA,* and *pqsR* was observed as compared to control *P. aeruginosa* culture (*p* < 0.0001). Furthermore, the tested drugs significantly decreased the expressions of *S. typhimurium* sensor kinases genes *qseC* and *qseE* as compared to untreated control culture (*p* < 0.0001). 

### 2.7. Metoprolol Protects Mice against P. aeruginosa and S. typhimurium

**Figure 18:**
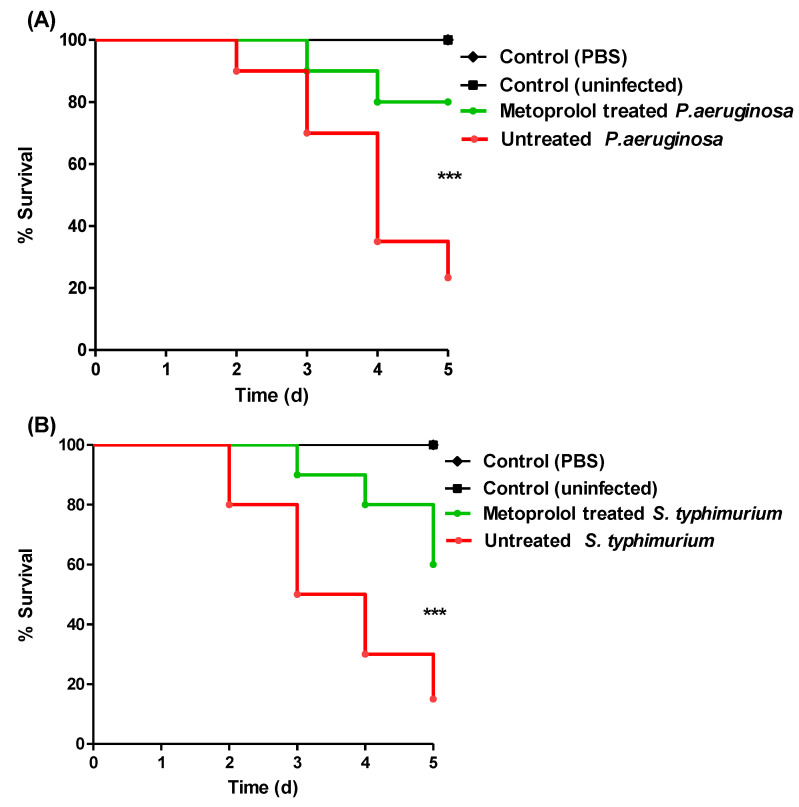
Protection of mice from (**A**) *P. aeruginosa* and (**B**) *S. typhimurium* by metoprolol. The survival of mice in each group (*n* = 10) was reported every day for 5 days and plotted using the Kaplan–Meier method and the significance (*p* < 0.05) was examined using the log-rank test. All mice in the negative control groups survived, while only 30% or 20% of mice survived in the groups that were injected with *P. aeruginosa* and *S. typhimurium,* respectively. Meanwhile, 80% or 60% of mice injected with metoprolol-treated *P. aeruginosa* or *S. typhimurium* survived, conferring 50% or 40% protection in comparison to the mice injected with untreated *P. aeruginosa* and *S. typhimurium*, respectively. Log-rank test for trend (*** = *p* < 0.0001).

The metoprolol protective activity against *P. aeruginosa* and *S. typhimurium* virulence was in vivo evaluated. All mice survived in the negative control (uninfected or PBS injected) groups. Meanwhile only three mice survived after injection with untreated *P. aeruginosa*, metoprolol conferred the protection to eight mice (Figure 18A). Furthermore, metoprolol protected six mice injected with *S. typhimurium* in comparison to eight deaths in the control group injected with untreated *S. typhimurium* (Figure 18B). The treatment of *P. aeruginosa* and *S. typhimurium* with metoprolol at sub-MIC significantly decreased their capacity to kill mice (*p* < 0.0001) using the log-rank test for trend.

## 3. Discussion

Antimicrobial resistance is a growing global health threat particularly in the diminished supply of new agents [1]. Conquering bacterial resistance requires the adoption of new treatment strategies and more efficient approaches to facilitate microbial eradication [4]. Targeting of bacterial virulence is one of the most resourceful approaches to diminish the increasingly microbial resistance [38,39]. Bacterial communities employ QSs to harmonize their responses competently and circumvent the host immune systems. These QSs systems produce diffusible signal molecules, called autoinducers (AIs) that bind to their cognate receptors to orchestrate the expression of bacterial virulence to guarantee maximum chances of survival [3,8,10]. Parallelly, bacteria use an array of sensors to accommodate themselves with their environment. For instance, bacteria can eavesdrop on the signaling systems in mammalian cells such as neuroendocrine (NE) stress hormones adrenaline and noradrenaline via membranal sensor kinase receptors [13,14,40]. Most interestingly, bacterial pathogens exploit these NE as signals to regulate the expression of their virulence genes [14,41]. For example, in enterohemorrhagic *E. coli*, adrenaline and noradrenaline can substitute for autoinducers (AI-3). This observation emphasize the existence of crosstalk between the two signaling systems and the presence of adrenergic receptors on the bacterial membranes [40,42]. Moreover, both adrenaline and noradrenaline enable bacteria to acquire iron from the host, as they are ferric iron chelators which release the iron from transferrin and lactoferrin [43]. Furthermore, bacterial lipopolysaccharides activate the macrophage to induce the release of adrenaline and noradrenaline and hence augment the bacterial pathogenesis [44]. Bearing in mind all the above findings, adrenergic blockers are suggested as promising candidates to target bacterial virulence, they can at least decrease the bacterial espionage on the mammalian signaling systems. The possibility of repurposing approved safe adrenergic blocker drugs to target bacterial virulence can decrease efforts, time, and costs. In this work, we further aimed to explore the anti-QS activities of adrenergic *β*-blockers against Gram-negative bacteria which often employ QS LuxR-type receptors to sense a wide array of AIs [9,10].

Molecular docking simulation was conducted to investigate the affinity of 22 FDA-approved *β*-blockers towards the QSs of three highly resistant bacteria species. The adopted 133.74 kDa TraR biological target from *A. tumefaciens* is a homo-2-mer-A2 transcription factor solved at 1.66 Å atomic resolution as a heterocomplex with its respective DNA-binding site and pheromone (Supplementary Data, Figure S5A). The other adopted QSs, QscR *P. aeruginosa* (55.19 kDa) and CviR *C. violaceum* (120.20 kDa), are cyclic protein homodimers solved at 2.55 Å and 3.25 Å, respectively, with their respective binding ligands (Supplementary Data, Figure S5B,C). The three biological targets are quite similar comprising of the *N*-terminal α-helix/*β*-sheet/α-helix sandwiched ligand binding domains and α-helix/*β*-turn/α-helix motifs for specific DNA binding sites down to the *C*-terminus. Ligands are fully embedded within the protein targets and are virtually deprived from solvent contacts, allowing them to form several hydrophobic interactions with pocket lining residues in addition to few hydrogen bonding either being direct with polar residues or indirect through water bridges. The lactone heads of the co-crystalline ligands are settled within a small inner sub-pocket lining with polar residues that stabilize the hydrophilic head via hydrogen interactions as well as hydrophobic ones. The amide portion of the ligands’ bodies are anchored near to several polar residues allowing interaction with significant hydrogen bond donors/acceptors of the pocket residue sidechains. Finally, the crystallized terminal acyl tails are directed deep into a larger hydrophobic sub-pocket possessing of few polar residues.

Despite ligand binding domain similarity, the overall architecture of the adopted dimers is quite different where TraR shows perpendicular two-fold symmetry axis of each respective sub-unit resulting in pronounced overall complex asymmetry. On the other hand, both QscR and CviR exhibit near symmetry across their protomer architectures. The co-crystalline ligand of each target protein was used as QS reference inhibitor within our two-stage docking protocol. Literature reports have illustrated the potent inhibition activity of HLC allowing protection of *Caenorhabditis elegans* against the quorum-sensing-driven lethality mediated by the *C. violaceum* virulence microorganism [45]. This synthetic compound also showed superior antagonism for *C. violaceum* CviR over other tested inhibitors, *N*-octanoyl-_L_-homoserine lactone (C8-HSL) and C10-HSL, where the latter ligands showed partial antagonism in presence of CviR natural autoinducer, C6-HSL [25]. Additionally, Geske et al. showed significant antagonistic activity of HLC across three strains; TraR *A. tumefaciens*, LasR *P. aeruginosa*, and LuxR *Vibrio fischeri* which was preferentially higher than most other closely related synthetic multi-target antagonists [46]. Since HLC is an available potent co-crystalline wide-range LuxR-type QS inhibitor, the ligand was also adopted as an additional relevant reference standard for the other two target proteins. Therefore, the adrenoreceptor blocker exhibiting more negative docking energies than the reference co-crystalline ligands and HLC, as well as illustrating comparable binding modes relative to HLC, would be highly suggested to confer preferential competitive inhibition potentiality towards these QS targets.

The depicted differential ligand orientation/conformation for the investigated ligands across the three biological pockets were rationalized for the small-sized binding site of TraR *A. tumefaciens* in relation to other targets. This was clearly reported by Geske et al., where investigating several synthetic antagonists across three QSs proteins revealed that the TraR *A. tumefaciens* ligand-binding domain appeared the most restrictive being highly sensitive to the length of pheromone’s acyl group [46]. This was further confirmed via the CASTp server analysis, where as expected, the TraR *A. tumefaciens* was assigned the least accommodating, whereas the largest pocket volume was assigned to CviR *C. violaceum* having its ligand extend into the solvent region. On the other hand, the pocket of QscR *P. aeruginosa* was of tighter packing density, the thing that is consistent with the reported binding modes of different pheromones [24]. It worth noting that the CASTp server analysis showed that the CviR’s large-spaced pocket was wide rather than being elongated or narrow which was the case of the QscR pocket. This finding was confirmed through conformational analysis of the obtained docking poses where the docked ligands at the CviR’ pocket adopted an inverted L-shaped conformation rather than the almost linear one being seen by the same ligands at the QscR pocket. Despite the differential pocket size/topology of both QscR *P. aeruginosa* and CviR *C. violaceum* target proteins, several ligands (1, 3, 5, 7–10, 17, 18, 21) showed comparable binding energies across both targets. Nevertheless, the rest of the investigated ligands, particularly compounds **11**, **13**, and **14**, predicted preferentially higher docking energies for the QscR binding site. The latter findings could confer the significant impact of ligand’s topology, type/number of ligand’s substitutions, and the nature of pocket lining residues on the ligand–protein bindings and pocket accommodation.

It worth mentioning that the second sophisticated docking approach provided validation of the docking workflow where redocking the co-crystalline ligands through the same adopted second-stage docking protocol revealed significantly low RMSDs. To our delights, the RMSD values of redocked co-crystalline ligands were 1.0332 Å, 1.2416 Å, and 1.5834 Å at TraR *A. tumefaciens*, QscR *P. aeruginosa*, and CviR *C. violaceum*, respectively, with great superimposition binding modes (Supplementary Data, Figure S6). Clearly, depicting RMSD values below 2.0 Å indicates that both the adopted docking parameters and algorithms were sufficient for determining the best docking pose [47]. Therefore, findings obtained out of the adopted directed docking protocol was confirmed valid, ensuring the biological significance of the obtained docking binding modes and in turn their respective docking energies. It worth mentioning that the second-stage docking analysis further validated the preliminary docking results where the obtained hits still exhibited higher docking scores than those of the reference ligands across this sophisticated docking step. In this regard, it was of great importance to thoroughly investigate the depicted ligand–protein interactions in correlation with the obtained valid docking scores. The latter would provide valuable insights regarding the ligands’ structural characteristics impacting their respective pocket binding.

Differential docking scores across the investigated hits against TraR *A. tumefaciens* were significantly correlated to the ligands’ terminal aryl groups rather than their *N*-substituted propanolamine scaffolds. Despite the fact that Gln58 is one of the limited polar residues of the large hydrophobic site that can be even charged under physiological condition, such amino acid depicted van der Waal interactions via their side chain C*β* and C*δ* atoms with certain investigated ligands including compound **1** and the reference inhibitor (HLC). On the contrary, ligands with specific terminal aryl moieties managed to furnish polar contacts with the few hydrophilic residues lining the large hydrophobic site (Thr51 and Gln58). The latter was illustrated for the top-docked ligands, 10, 11, and 14, where extended polar networks were maintained with the sidechains of Thr51, Gln58, and/or the mainchain of Phe62 residue through the ligands’ terminal polar substitutions. Depicting these polar interactions at the target’s hydrophobic was suggested to satisfy the H-bonding or ion-pairing potentiality of the limited hydrophilic residues comprising this large hydrophobic site.

Notably, compound **8** with its hydrogen bond acceptor morpholine ring showed no predicted hydrogen bonding with the large site lining residues. It was suggested that the deep anchoring of the morpholine nucleus being directed towards the site’s polar residues (Thr51 and Gln58) would lessen the electrostatic penalty assigned for the ligand at such highly hydrophobic site. The latter was reasonably translated into an intermediate docking energy of −7.4923 Kcal/mol being only second to the top-docked ligands. The opposite could be translated for other short-tailed adrenoreceptor blockers which failed to achieve better docking scores than the reference inhibitor (HLC) at the TraR’s target pocket. Having their polar functionalities such as sulphonamide, carboxamide, or ester groups close to their central aryl core, compounds such as **9**, **15**, and **16** were predicted to lack the relevant closeness towards the site’s polar residues (Thr51 and Gln58) that allows favored ligand–protein and high docking score. On the contrary, extended tails with terminal hydrocarbon chains, as incorporated within compounds **12**, **13**, **17**, and **18,** were predicted to impose steric hinderance at the TraR tight pocket as well as being unable to compensate the electrostatic penalty assigned for these ligands at such a highly hydrophobic site. In brief, ligands of proper length terminal aryloxy scaffold and harboring polar substitutions at close proximity towards the hydrophobic sub-pocket’s polar residues could be translated into significant ligand anchoring and potential stability at the TraR *A. tumefaciens* binding site.

The above ligand–target preferential bindings came in great agreement with current literature were the above-described interactions were thoroughly reported as being important for binding several small molecule inhibitors at the TraR *A. tumefaciens* binding site. Both Trp51 and Asp70 sidechains at the TraR’s small sub-pocket were found essential for mediating polar contacts with crystalline ligand’s lactone ring. Further ligand stability was mediated through polar interaction with the two carbonyl moieties of the O-C8-HSL body [23]. Several solid phase organic synthesized analogues of *N*-sulfonyl- and *N*-nicotinyl-_L_-HSL reported by Kim et al. exhibited significant hydrogen bonding with Tyr53 polar sidechain. The docking results of these were highly correlated with their significant in vivo inhibition activity against TraR *A. tumefaciens* via reporter systems as well as anti-biofilm activity towards *P. aeruginosa* [48]. The authors reported high biological activities for the derivatives mediating the above-mentioned polar interactions. Similarly, derivatives of 2,2-dimethylbutanoyl-, *N*-(fluoroalkanoyl)-, *N*-(sulfanyl ethanoyl)-, and *N*-(fluorosulfonyl)-_L_-HSL showed significant antagonism against TraR quorum sensing of *A. tumefaciens* as per furnishing polar contacts with Thr129, Tyr53, Tyr57, Gln58, and/or Tyr61 confirmed through molecular modelling studies [49]. Molecular docking investigation of several 4-quinolone and phenazine-based analogues illustrated the important role of several pocket’s polar residues including; Tyr53, Trp57, Tyr61, Asp70, and/or Thr129, providing good explanation for their differential in vitro anti-quorum sensing activity in relation to respective docking scores [50]. The above reported polar interactions were also consistent with several anthraquinone- and chromone-derived active components of the traditional Chinese medicines possessing antibacterial activities and being identified as promising anti-QSTR agents through structure-based virtual screening, in vitro inhibition of bacterial biofilm formation, and/or proteolysis of bacterial quorum sensing signal receptor approaches [51,52].

Preferential docking scores for the investigated ligands at QscR *P. aeruginosa* in relation to TraR *A. tumefaciens* were reasoned for the earlier where residues at the large hydrophobic sub-pocket impose less steric hinderance for anchoring the ligand’s terminal aromatic/heterocyclic groups. This also had a significant impact on ligand–target binding since hits exhibited more extended polar networks with the QscR’s lining residues as compared to those of the TraR binding site. Such observation could confer the higher comparative importance of hydrophilic interactions as an important driving force for anchoring small molecules within the QscR binding pocket. Significance of binding to Ser38 was highlighted through our docking studies which was also reported crucial for determining the signal specificity of QscR, where this polar uncharged residue can guide the preferential binding of native 3-O-HSL over the unsubstituted native ligands [24]. Thus, significant affinity towards the QscR pocket site has been suggested for the investigated adrenoreceptor hits depicting relevant hydrogen bonding with Ser38 residue. Ligand–protein target interactions were thoroughly investigated to explore the differential docking scores across the investigated hits. Since hydrophobic interactions were illustrated to be conserved for all docked ligands, it was suggested that polar-directed binding was of more significant impact on the ligand/pocket accommodation. The latter was reasoned since several high-docking scored adrenoreceptor hits (−9.3164 up to −9.7616 kcal/mol) showed highly ordered and more extended polar contacts with the QscR lining residues. Compound **13** predicted significant double polar interactions with Asp75 and Ser38 as well as relevant hydrogen bonding with Tyr58, Trp90, and Ser129 all being mediated via its 3-oxo-propanol central scaffold. A comparable pattern of extended polar interactions was depicted for compounds **14** and **17** where Ser38, Tyr58, Asp75, and/or Ser129 showed preferential strong hydrogen bond distances and angles. The docking of these three ligands were further fortified via extended non-polar interactions with aromatic/heterocyclic as well as alkyl chained residues. The latter confer the important balance between polar and non-polar interaction for mediating best docking scores for the ligand/QscR binding. 

It worth mentioning that the polar substitutions at the tail part of compounds **14** and **17** showed relevant polar interactions with specific hydrophilic residues (Arg42 and Thr72) at the large hydrophobic QscR sub-pocket. Despite possessing polar oxygen functionality at its tail part, compound **13** could not depict relevant polar contacts at the QscR distal sub-pocket in similar fashion to the other two top-docking ligands. Such differential binding mode could be due to possible steric clashes imposed by the longer terminally branched alkyl ether chain of compound **13** which would disfavor its close contact with the pocket’s specific hydrophilic residues (Arg42 and Thr72). The latter docking poses were reasoned to minimize potential steric penalties due to the presence of branched bulky hydrophobic residues (Tyr52, Val78, Leu82, Ile125, and Arg42) at the distal end of the QscR binding pocket. On the contrary, compounds **13** and **17** possess shorter linear terminal substitutions (methoxyethyl and butylamido, respectively) the thing that would favor unhindered anchoring of their respective terminal polar functionalities near the polar residues at the distal pocket. A similar differential binding mode was also obvious for compounds **10**–**12**, where the latter exhibited highly hindered orientation at the distal hydrophobic sub-pocket. Having a long four-atom distance alkyl ether chain with a terminal bulky cyclopropyl moiety as its crown, compound **12** predicted upright conformation of its terminal tail rather than a linear coplanar one in relation to its central aromatic ring. Thus, compound **12** lacked polar contacts with distal polar residues, yet compounds **10** and **11** predicted hydrogen bonding with Tyr52 and Arg42, respectively. These binding modes were reasonably translated into a lower docking score for compound **12** (−8.2918 kcal/mol) while higher comparable docking energies for compound **10** and **11** (−8.9102 and −8.9182 kcal/mol, respectively). The above depicted size-directed binding mode for the investigated ligands was highly rationalized through reported literature where the distal portion of QscR binding pocket impose preferential binding for short chained acyl native ligands rather than large steric ones [24]. Single amino acid mutagenesis for these distal branched bulky hydrophobic residues with more bulkier residues showed a significant reduction of QscR activated signaling in response to autoinducer with longer acyl chains. 

Significant docking findings were also represented for the largest size docked adrenoreceptor hits, compound **21**. Compared to its aryloxy propanol-based member, compound **21** exhibited reversed orientation where it quaternary alkylate nitrogen atom is positioned at the center of the QscR binding site rather than typical orientation at the small sized sub-pocket. The possession of a large extended flexible substitution on the ligand’s nitrogen head imposed disfavored anchoring at the smaller sized sub-pocket. The inherited flexibility of the *N*-substitution allowed the ligand to adopt a favored orientation at the distal large QscR pocket with predicted reduced steric clashes with the end branched bulky hydrophobic residues. However, adopting such reversed docking pose caused compound **21** to lose key polar binding interactions with Asp75 which in turn furnished a moderate docking score of −8.7880 Kcal/mol. In brief, ligands possessing a moderately sized terminal tail with polar functionalities and significant flexibility would exhibit favored maneuvers to bind at the QscR distal pocket with minimal steric clashes while being able to mediate relevant polar interactions with specific polar residues at the pocket end.

Interestingly, the above depicted ligand–QscR binding interactions were consistent with current literature where several promising anti-*P. aeruginosa* QS small molecules have depicted these kinds of interactions being correlated to significant in vitro biological activities. Screening hits reported by Xu et al. were novel anti-QS scaffold of substituted double phenyl rings and a central amide-based linker/spacer which showed significant *Pseudomonas aeruginosa* biofilm inhibition activities [53]. These promising hits were identified through combined pharmacophores of LasR antagonist and QscR agonist, followed by docking simulations. Consistent polar interactions were depicted between their amide linkers and sidechains of QscR homologous pocket residues; Trp62, Tyr66, and/or Asp75. Stability of these ligands were further mediated through π-mediated interactions with Tyr66 or Trp90, in addition to hydrophobic contacts with Ala41, Tyr52, Val78, Leu82, Ile125, and Met127. Similar polar interactions were illustrated by Sadiq et al. where several FDA-approved sulphonamide antibacterial agents and their carboxamide-based close analogues showed favored hydrogen bonding with QscR homologous residues; Trp62 and Asp75 as well as additional polar contacts with Tyr58 and Ser129 through docking and subsequent molecular dynamics simulations [54]. Significant π–π hydrophobic interaction with Tyr66 was also depicted stable for all sulphonamide antibacterial agents across the simulation studies. The above hydrogen bonding with Try58, Trp62, Asp75, and Ser129 was also considered significant for stabilizing a series of triphenyl-structured antagonists within the ligand binding domain of *P. aeruginosa* QS protein at preferentially higher binding energies than a synthetic triphenyl mimic super-inducer, TP-1 [55]. The triple aromatic-based pharmacophores of these ligands further permitted favored ligand anchoring within the binding site through face-to-face hydrophobic interactions with Tyr66, Trp63, Trp90, and/or Phe101 residues. The in vitro LasR-reporter gene assay came to recapitulate their in silico findings, revealing significant antagonistic activity of these triphenyl-based compounds in the presence of native autoinducer, O-C8-HSL.

Moving towards the obtained *β*-blocker/CviR binding complexes, the differential docking scores of these ligands at CviR in relation to the other QS could be reasoned to the inherited differences within each pocket size and topology. Our CASTp analysis showed that both CviR and QscR pockets are large-sized spaces owing to the relevant orientation of the large hydrophobic sub-pocket residues imposing minimal steric hinderance against the anchoring of the ligand’s terminal aromatic/heterocyclic groups. In this regard, our investigated ligands at the CviR active pocket exhibited higher docking scores as compared to their respective positions at the TraR binding site. On the other hand, the CviR terminal sub-pocket is considered wider at its large hydrophobic sub-pocket as being compared to that of QscR. This would further allow less steric hindering binding and more favored pocket orientations for the bulky *β*-blocker agents the thing that was highly obvious for the second generation *β*-blocker (Compounds **19**–**21**). The latter compounds exhibited significant steric groups like the large aliphatic-associated aromatic substitution at their respective quaternary nitrogen heads. These bulky ligands managed to exhibit higher docking scores at CviR in relation to QscR, where they managed to establish favored polar interactions with key residues, including catalytic Asp97 at CviR, the thing that was missing for compound **21** at QscR pocket. Nevertheless, the wider CviR’s pocket topology rather than being narrow and elongated seemed to allow several small-sized ligands to be at far proximity from the pocket’s lining residues making them unable to mediate relevant ligand–target binding interactions. The latter was confirmed since several investigated *β*-blockers (compounds **10**, **11**, **13**, and **14**) depicted higher docking scores at the relatively narrower, more elongated QscR pocket rather than at the CviR binding site. Notably, the obtained ligand–CviR docking scores were correlated with less extended polar interaction networks for these investigated small-sized ligands as compared to QscR. Based on such docking behavior, it was suggested that polar-directed binding has a significant impact on the ligand/pocket accommodation at different QS proteins. The latter pocket structures differences and their impact on ligand binding were also highlighted by Lintz et al. where they investigated the comparative architectures of the ligand-binding domains and AHL-related binding pockets of several QS including CviR, QscR, LasR, TraR, and SdiA [24,56]. 

Differential docking analysis between investigated ligands at CviR has correlated better ligand’s docking scores to the extent and magnitude of furnished polar contacts between the ligands and conserved key polar residues. One of the top docked small-sized ligands, compound **10**, showed highly ordered polar contacts with Tyr80, Asp97, and Ser155 via its quaternary nitrogen head and free hydroxy group of its propanolamine linker. Despite that, the latter polar network was also assigned to other *β*-blockers, compounds **2**, **11**, **13**, and **14**, yet these ligands depicted lower docking scores as compared to compound **10** (−8.9128 Kcal/mol versus −8.0192, −8.2830, −8.6064, and −8.7912 Kcal/mol). Such differential docking behavior was suggested for the extra polar contacts mediated by the terminal polar functionalities at the compound **10** tail chain which furnished significant hydrogen bond pairing with Met89 and Met253 at the large hydrophobic sub-pocket. Similar to QscR-associated binding modes, polar interactions with these large hydrophobic sub-pocket residues could have minimized the potential steric penalties during ligand anchoring which are mainly mediated by the branched bulky hydrophobic residues (Leu57, Leu72, Val75, Leu85, Ile153, and Val250) at the distal end of the CviR binding pocket. On the other hand, compounds **11**–**14** lacked these kinds of terminal polar interactions despite sharing significant polar substitutions (ester, ether, or diether) at their respective tail scaffolds. The latter docking result could be reasoned for the end alkyl groups (methyl, ethyl, or cyclopropyl) at compounds **11**–**14** that could disfavor the proper orientation of the ligand’s tail towards these end terminal polar residues as a result of repulsion forces. However, this was not the case for the several same ligands at QscR pocket where compounds **11** and **14** exhibited polar interactions with large pocket end residues, Arg42 and Thr72. Thus, it was suggested that the CviR wide topology might have either brought the end polar residues at far distances or non-proper orientations/angles from the ligand functionalities causing compounds **11**–**14** to just miss such relevant binding interactions. 

It was interesting that despite the lack of polar interactions with the CviR terminal polar residues, compound **17** managed to achieve a high docking score (−9.0849 Kcal/mol) which was even more profound than that obtained by compound **10**. Despite the fact that both compounds **10** and **17** possess the carboxamide moiety at their terminal alkyl chains, only compound **10** managed to predict significant binding interaction with large sub-pocket terminal residues. This was reasoned for the hydrophobic terminal alkyl group at compound **17** being next to the carboxamide moiety which could impose repulsive penalties against these polar amino acids. Both CviR complexes with compounds **10** and **17** were stabilized via comparable pattern of extended non-polar interactions with aromatic/heterocyclic as well as alkyl chained residues. Nevertheless, it was noticed that only compound **17** managed to achieve more extended π-mediated hydrophobic contacts with several non-polar pocket residues, Tyr80 (π-π) Tyr88 (π-π), and Trp111 (π-H), the thing that could be correlated to its high docking energy. In these regards, it was concluded that a balance between polar and non-polar interactions mediated by a certain ligand would be efficiently translated into best docking scores for the ligand/CviR binding.

Validity of the obtained residue-wise ligand/CviR interactions was assured since several reported studies depicted comparable ligand/residue profiles during their quest for identifying new potential CviR-based quorum sensing inhibitors form synthetic, natural, and/or chemical library sources. Structural-based screening hits obtained from Mu.Ta.Lig Virtual-Chemotheca and ZINC/FDA-approved databases showed significant energy contributions of Met72, Tyr80, Trp84, Leu85, Tyr88 and/or Ser155 within the ligand free-energy binding calculations [57]. The four top performing ligands shared a common structural topology of central heterocyclic ring core (triazole or piperazine) flanked on both of its sides with terminal aromatic/heterocyclic scaffolds having variable polar substitutions. Interestingly, the negatively charged Asp97 residues which is considered crucial for native ligand binding was of lower, yet still relevant, contribution for stabilizing the identified promising hits at the CviR *C. violaceum* binding site. Another study identified several flavonoid and chalcone-based hits from natural product libraries as promising inhibitors of the CviR quorum sensing protein through virtual-screening as well as in vitro violacein and biofilm inhibition biological assays [58]. These promising natural-based hits exhibit preferential polar interactions with Trp84, Asp97, Met135, and Ser155, in addition to relevant hydrophobic contacts towards Tyr80, Leu85, Tyr88, Met89, Trp111, Phe115, and Phe126, which have been successfully translated into high biological findings. Comparable residue-wise binding interactions were reported for several chemically synthesized 2-imidazoline/oxazoline-based analogues through molecular docking and dynamics simulations [59]. The compounds predict favourable polar binding contributions for Trp84, Asp97, Tyr88, Ser155, and Pro189 residues with the ligands’ 2-imidazoline/oxazoline polar heads. Hydrophobic π-mediated interactions with Tyr111 and Phe126 pocket residues were significant for these synthesized ligands. Finally, the active natural metabolites isolated from *Passiflora edulis* ethyl acetate extract showed relevant accommodation of the CviR binding site, particularly for hexadecanoic acid methyl ester [60]. Polar interactions with Tyr84 as well as hydrophobic contacts with Ile57, Tyr80, Leu85, Tyr88, Ile99, Trp111, Phe115, Met135, and Ile153 were found consistent across the top active metabolites. The letter suggested the important role of these residue-wise binding interactions for mediating promising in vitro *C. violaceum*-oriented inhibition activities.

Following our depicted docking study, we aimed to investigate the thermodynamic stability of the predicted *β*-blocker/QS complexes. Throughout the 100 ns all-atom MD runs, the examined agents illustrated significant global stability within the three target’s canonical binding site as being confirmed through the monitored *Cα*-RMSD trajectories. All the above-described dynamic behaviours of the investigated target proteins indicate the successful convergence of the target proteins across the designated MD simulation timeframe. Moreover, the above-depicted protein’s RMSD tones also infer that successful system minimization, relaxation, and thermal equilibration stages have been adopted before the MD production step and thus, no further extension of the MD simulation beyond the 100-ns period was needed. Achieving steady ligand’s *Cα*-RMSD tones as well as the rapid attaining of dynamic equilibrium for more than 50 ns, all highlighted the significant ligand’s retainment within the protein active site, the thing that was highly comparable to the potent QS inhibitor, HLC. The latter ligand–pocket confinement was further confirmed through the illustrated conformational analysis where limited ligand orientation shifts were depicted within any of the three QSs binding site. However, preferential ligand’s conformational/orientation stability were assigned to QscR and CviR as compared to the TraR binding site. The latter could explain the depicted comparatively lower *Cα*-RMSD values for the same ligands at QscR *P. aeruginosa* or even CviR *C. violaceum* in relation to TraR *A. tumefaciens* binding sites. In this regard, it was suggested that these preferential ligand accommodations could be highly correlated to differentiate the pocket size. The latter dynamic behavior further confirmed the reported TraR *A. tumefaciens* pocket constriction as well as great sensitivity towards the length of pheromone’s acyl group [46]. It is worth mentioning that ligand’s versus respective protein’s *Cα*-RMSD trajectories were not more than 1.5-fold, the thing that further confirms the successful convergence of ligand–protein complexes and ligand–pocket confinement, as well as inferring the suitability of 100 ns MD simulation runs requiring no further extension.

Findings obtained from monitoring the RMSFs came in great concordance with the above- mentioned RMSD-based stability analysis. To our delight, the depicted comparable residue-wise flexibility modes across the three investigated bacterial LuxR-type QSs based on the ΔRMSF analysis would highlight the validity of the MD simulation study and adopted protocol. Additionally, the illustrated higher immobility profiles for the residue regions towards the carboxy terminal as compared to *N*-terminus conferred the inherited preferential stability of the DNA binding domain over the ligand anchoring one. This was in good concordance with the reported conformational stability analysis of both domains at the LuxR-type QS proteins [23,24,25]. The significantly high stability profiles for pocket residues as well as vicinal residues across the corresponding regions 70–90 and 100–130 inferred the significant influence of ligand’s binding upon the stability of these residue ranges. It worth noting that these residue ranges were proven to possess relatively conserved hydrogen bond interactions among the constituting residues as well as with the binding ligands [23,24,25,58,61,62,63]. On the other hand, the flexible residue ranges around 130–145 and 170–180 are at regions being at distance of >15 Å from the binding site residues, indicating the capability of the active site to accommodate bulkier inhibitors. As a final observation, several pocket lining residues which were reported as relevant for ligand anchoring showed significantly high immobility profiles being consistent across several simulated ligands. The latter dynamic behavior highlights the pivotal role of these residues for the stability of ligand within the protein’s binding site. Moreover, the majority of these ligand-conserved inflexible pocket residues are hydrophobic in nature the thing that confers the significant role of the large hydrophobic pocket as well as the structure of the ligands terminal chain for mediating stabilized ligand–target complexes [63]. Nevertheless, selected polar pocket residues (Ser, catalytic Asp and others) were also shown with significant inflexibility which would emphasize the importance of these amino acids to satisfy the polar functionality of the ligands as well as permitting selectivity for their respective binding [25].

Interestingly, the above-described pocket’s residue-wise flexibility profiles and preferentiality for hydrophobic contacts in stabilizing the simulated ligands were also highlighted within the MM/PBSA binding free energy calculations. Both QS pocket’s lining residues and vicinal amino acids showed the favored contribution (high negative values) within the ligand–protein binding energy, the thing that implied significant ligand confinement within the target binding sites. Dominance of Δ*G*_van der Waals_ over the electrostatic energy contributions conferred the significant role of hydrophobic contacts with the QSs large hydrophobic sub-pocket to stabilize the binding ligands. Additionally, the nature of the top-energy contributing residues is mostly hydrophobic which further emphasizes the predominance of the van der Waals potentials for binding the investigated ligands deep into the target binding site. This came in great agreement with the reported data within the current literature considering the LuxR-type QSs pocket to be more hydrophobic in nature being deep, and with conserved hydrophobic pocket lining residues [23,24,25]. However, the strong polar contacts with the catalytic Asp residue as well as key polar pocket residues provided a significant role of the Coulomb’s electrostatic potential energy for enforced ligand–protein biding.

Notably, the *β*-blocker members possessing higher numbers of polar functionalities (hydrogen bond donors and acceptors), as seen in compounds **8**, **10**, and **11**, were able to furnish higher Δ*G*_Electrostatic_ as compared to other drug class members. The latter would be reasoned for the ability of the earlier compounds to satisfy the electrostatic potentiality of polar lining residues at the small QSs sub-pocket as well as at the terminal part of the large hydrophobic site. On the contrary, lignads with less polar potential, particularly at the terminal aromatic chains (HLC and compounds **1**, **12**, **13**, and **14**), illustrtaed beneficial lower polar solvation energies (Δ*G*_Solvation; Polar_) that favoures their respective relevant target binding since the latter is a solvent-substitution process. In this regard, the *β*-blocker agents with balanced hydrophobic/polar functionalities, particularly at their terminal aromatic schaffold, would mediate favoured non-polar contacts with the QSs large hydrophobic pocket and polar interactions with hydrophilic lining residues being vicinal to the small more polar QSs sub-pocket, while as minimize any potential solvation energy penealties that would compromise the ligand’s anchoring process.

Finally, the differential pocket topology across the three investigated QSs was highlighted via the estimated binding free energies where the same ligand depicted higher binding energies at QscR following by CviR and then TraR. This was in corcordance with the initial docking findings suggesting preferential *β*-blockers’ affinity towards the narrow elengated QscR pocket. Additionally, the obtained higher total non-polar interactions (Δ*G*_van der Waal_ plus Δ*G*_SASA_) for ligands at the QscR and CviR in regard to TraR binding sites would have been directly related to the larger surface area of the earlier pockets. Thus, the ability of *β*-blocker agents to attain more extended conformation within the target’s pocket would be beneficial for effeceint binding at QscR and CviR, in relation to TraR. This has been throughly explained through presented docking simulation, pocket size analysis, as well as reported literature [24,46].

Based on the findings of the in silico molecular study; atenolol, esmolol, and metoprolol (compounds **10**, **11**, and **14**, respectively) were selected to be further investigated for their anti-virulence and anti-QS activities. The biosensor *C. violaceum* CV026 is usually employed to assess the QS in Gram-negative bacteria due to its ability to release the pigment violacein in response to acyl-homoserine lactones under the CViI/R QS system control [18,37]. Initially, the anti-QS effects of three tested *β*-blockers on violacein production were assessed. In great compliance with the molecular docking findings, three of the tested drugs significantly diminished the production of violacein pigment indicating the predicted anti-QS activities of the tested drugs. It is worth noting that the tested *β*-lockers were assayed at their sub-MIC in all the tests to exclude any effect on the bacterial growth.

For attesting the anti-QS activities of the tested *β*-blockers, two famed pathogenic Gram-negative bacterial models *P. aeruginosa* and *S. typhimurium* were chosen for this purpose. *P. aeruginosa* is the causative agent of diverse types of infections including eye, wound, and respiratory infections [17]. Moreover, the elevated levels of resistance to various antibiotics and disinfectants books a place for *P. aeruginosa* among the major global health concerns [15]. *P. aeruginosa* employs three QS systems, two LuxI/LuxR types (LasI/LasR and RhlI/RhlR), and one non-LuxI/LuxR PQS system in addition to QscR (LuxR homolog) [9,10]. The other Gram-negative bacteria *S. typhimurium* was chosen because of its pathogenesis and also for its different QS system. Different *Salmonella* spp. cause a wide range of infections from localized to systematic infections [64]. *Salmonella* spp. do not synthetize their own AHLs; but they acquire a *sdiA*-encoded functional AHL receptor that responds to AHLs [65,66,67]. In vitro, *S. typhimurium* respond to AHLs in a sdiA-dependent manner to activate *sdiA*-regulated genes (srgs) to regulate the bacterial virulence [66,67]. Interestingly, *S. typhimurium* harbors adrenergic sensor kinases QseC and QseE that bind to adrenaline or noradrenaline which greatly enhance the virulence [14,41,68], these responses can be inhibited by adrenergic *β*-blockers. Interestingly, the tested *β*-blockers down-regulated the expression of *qseC* and *qseE* genes in *S. typhimurium* indicating the ability of tested drugs to diminish the bacterial eavesdrop on host cells that results in mitigating the bacterial virulence. 

In Gram-negative, autoinducers bind to their cognate receptors forming to complexes which in turn bind to bacterial chromosome at what are called lux boxes regulating the expression of QS-controlled virulence encoding genes [11]. In this study, the three tested *β*-blockers significantly down-regulated the expression of QS receptor and inducer encoding the main three *P. aeruginosa* QS systems LasI/LasR, RhlI/RhlR, and PqsR/PqsA. These results are in compliance with the molecular docking findings which approve the ability of *β*-blockers to compete on QS receptors conferring a considered possibility to quench the QS activities. Bacterial biofilm formation is a QS-controlled virulence factor that enhances the bacterial pathogenesis as extensively reviewed [69], as they confer an additional increase in resistance to antibiotics [70,71]. The three tested *β*-blockers significantly decreased the biofilm formation by *P. aeruginosa* or *S. typhimurium*. To attest the anti-virulence activities of *β*-blockers, the in vivo ability of metoprolol to protect mice from *P. aeruginosa* or *S. typhimurium* was explored. Our results indicate that the treatment of *P. aeruginosa* or *S. typhimurium* with sub-MIC of metoprolol significantly reduced the bacterial capacity to kill mice.

## 4. Materials and Methods

### 4.1. Target Preparation and Ligand Construction for Docking Analysis

The designated adrenoreceptor blockers and reference antagonist were constructed within MOE2019.01 software package (CCG^TM^, Montreal, QC, Canada). The ligands’ respective isomeric/canonical SMILES strings, obtained from the PubChem database, were utilized to build the ligands. Each constructed ligand was energy-minimized through a conjugate-gradient approach of 2000 steps till reaching a root-mean-square gradient convergence of 1 × 10^−3^ Kcal/mol/Å^2^ using MMFF94s partial charges and MMFF94s-modified forcefield. Biological targets were obtained from the RCSB-Protein Data Bank TraR *A. tumefaciens* (PDB entry: 1L3L), QscR *P. aeruginosa* (PDB entry: 3SZT), and CviR *C. violaceum* (PDB entry: 3QP5). Proteins were structurally prepared via 3D protonation, as well as autocorrection of atoms types, partial charges, and bond connectivity at physiological pH [72]. Finally, MOE Loop modeler was used for modeling missing loops within the LuxR-type QscRs PDB files.

### 4.2. Two-Stage Multi-Target Docking Protocol

The binding site of each target was defined by the MOE-Alpha Site Finder geometrical approach while being refined for including the crucial residues reported in current literature. The size of the defined pockets for the TraR, QscR, and CviR quorum sensing transcription factor were of 51, 83, and 93, respectively, where these values indicate the number of *alpha spheres* (geometric features of the target’s Voronoi diagram) comprising each binding site [73]. The lining residues of TraR *A. tumefaciens* binding site include; Ala38, Tyr39, Leu40, Thr51, Tyr53, Trp57, Tyr61, Phe62, Asp70, Val72, Trp85, Phe101, Tyr102, Ala105, Ile110, Thr115, Met127, Phe128, and Thr129. Concerning QscR *P. aeruginosa* active pocket; Ser38, Phe39, Gly40, Ala41, Arg42, Tyr52, His53, Phe54, Ser56, Tyr58, Trp62, Lys63, Tyr66, Ile67, Thr72, Asp75, Ile77, Val78, Leu82, Trp90, Phe101, Trp102, Ala105, Ile110, Ile125, Met127, and Ser129. Lastly, CviR *C. violaceum* pocket residue involves; Leu57, Ile69, Gln70, Arg71, Leu72, Val75, Asn77, Tyr80, Trp84, Leu85, Tyr88, Met89, Ala94, Gln95, Asp97, Pro98, Ile99, Leu100, Arg101, Trp111, Phe115, Phe126, Ala130, Met135, Thr140, Ile153, Ser155, and Val250.

Docking workflow was performed on two stages, where the first was a rapid preliminary screening stage using MOE high-throughput virtual screening docking protocol for identifying the significant hits exhibiting more negative docking energies (Kcal/mol) in relation to reference antagonist. Throughout the virtual screening docking protocol, all protein residues were kept rigid and the ligand conformations were developed through a bond rotation method and ligand placement technique, within the defined active site, being guided via the triangular matcher protocol which is the most efficient approach for well-defined binding sites [74]. Finally, the obtained ligand conformations were ranked via the London_dG scoring system. The second more sophisticated stage was considered as a refinement approach which was performed for the preliminary identified hits of each target and proceeded through MOE induced-fit docking protocol for increasing the pose prediction accuracy of the preliminary identified hits. Adopting the induced-fit docking protocol allowed significant flexibility of residues building up the canonical binding pocket of each target protein. Both the triangular matcher and London_dG scoring function were utilized for ligand placement and initial scoring in the induced-fit (flexible) docking protocol. However, the top ten docked poses for each ligand were retained for subsequent refinement and energy minimization, within the target pockets, where only the sidechains of the protein residues were set to tethered within the forcefield configuration options. Following refinement, the poses were then rescored using GBVI/WSA_dG forcefield for a second scoring. The latter forcefield-based scoring function relies on explicit solvation electrostatics, current-loaded charges, exposure-weighted surface area, and Coulombic electrostatics via protein–ligand van der Waals scores [75,76]. High docking energy, RMSD values at 2.0 Å cut-off in relation to co-crystalline ligand, as well as significant interactions with reported crucial pocket residues were all considered for selecting the best docking pose of the investigated ligands. 

Visual inspection and protein–ligand interaction analysis for the furnished docking poses were achieved through using PyMol2.0.6 Graphical Visualization Software (Schrödinger^TM^, New York, NY, USA) [77]. The cut-off values for all polar hydrogen bond (Donor-H…Acceptor) were assigned at a respective angle (20°) and distance (3.3 Å) being optimal for hydrogen bond strength [78,79]. Hydrophobic interactions were determined via the MOE ligand interactions tool, in addition to manual measurements performed via PyMol bond distance measurement tools keeping a distance cut-off 5.0 Å measured from the nearest interacting ligand atom to residue’s α-carbon atom. 

### 4.3. Molecular Dynamics Simulations 

Models of promising hits or HLC, in complex bacterial QS were chosen as starting coordinates for 100 ns all-atom MD simulations using GROMACS-2019 software package using CHARMM36m forcefield and CHARMM-General Forcefield program for protein and ligands, respectively [80]. Each ligand–protein model was solvated within a TIP3P cubic box under periodic boundary conditions implementation with 10 Å marginal distance [81]. Residues of bacterial QS target protein were assigned at their standard ionization states under physiological conditions pH (7.4), while the net charge of the entire system was neutralized using sufficient numbers of potassium and chloride ions being added via Monte-Carlo ion-placing approach [82]. 

Constructed system were minimized for 5 ps under the steepest descent algorithm double-staged equilibration for 100 ps easch, and production at 100 ns. First-stage equilibration was proceeded under a constant number of particles, Volume, and Temperature (NVT) ensemble (303.15 K; Berendsen temperature coupling method), while as the second equilibration stage was performed under a constant number of particles, Pressure, and Temperature (NPT) ensemble (303.15 K, 1 atmospheric pressure; Parrinello–Rahman barostat method). A force constant of 1000 kJ/mol.nm^2^ was used for preserving original protein folding and restraining all heavy atoms during the minimization and equilibration processes. Production stage involved 100 ns MD simulation runs under (NPT ensemble using the Particle Mesh Ewald algorithm for computing the long-range electrostatic interactions [83]. All covalent bond lengths, including hydrogens, were modeled under the implemented linear constraint LINCS method with s fs integration time step [84]. Both Coulomb’s and van der Waals non-bonded interactions were truncated at 10 Å using the Verlet cut-off scheme [85]. 

Both RMSD and RMSF analyses were estimated using GROMACS built-in tools. The ΔRMSF was estimated for each ligand-bound protein relative to the bacterial QS apo/unliganded state (ΔRMSF = RMSF_(apo—holo)_) being simulated at same procedures as the holo state proteins. GROMACS “*g_mmpbsa*” module and was used to estimate the ligand–protein binding free energy using the MM/PBSA calculation [36]. The MM/PBSA calculations of all simulated systems were applied on representative frames for the whole MD simulation runs (100 ns). For representing the ligand–protein conformational analysis across specific timeframes, the PyMol2.0.6 was used.

### 4.4. Chemicals, Microbiological Media and Bacterial Strains

All microbiological media, Luria-Bertani (LB) broth and agar, Mueller Hinton (MH) broth and agar, and Tryptone soya broth (TSB) were obtained from Oxoid (Hampshire, UK). *P. aeruginosa* PAO1 (ATCC BAA-47-B1), *C. violaceum* CV026 (ATCC 31532) and *S. enterica* serovar *typhimurium* (NCTC 12023) were used in this study. *β*-blockers atenolol, esmolol, and metoprolol (CAS Numbers: 29122-68-7, 81161-17-3 and 56392-17-7, respectively) were ordered from Sigma-Aldrich (St. Louis, MO, USA). All the chemicals used were of pharmaceutical grade.

### 4.5. Determination of MICs of β-Blockers, and the Effect of β-Blockers at Sub-MIC on Bacterial Growth

To determine the MICs of tested *β*-blockers, the agar dilution method was employed according to the protocol of Clinical and Laboratory Standards Institute (CLSI, 2015) [86]. To ensure that the tested *β*-blockers had no influence on the growth of *P. aeruginosa*, *S. typhimurium* or *C. violaceum*, the effects of 1/4MIC of tested drugs on bacterial growth were evaluated [64]. Briefly, overnight cultures of each bacterial strain were inoculated in LB broth provided with atenolol, esmolol or metoprolol at sub-MIC, and cultured at 37 °C for 24 h. The turbidity of bacterial cultures was measured at 600 nm.

### 4.6. Assy of Violacein Production

To evaluate the anti-QS activities of tested drugs, their abilities to inhibit the production of QS- controlled violacein pigment by *C. violaceum* were assessed as described earlier [5]. Briefly, LB broth aliquots (100 µL) containing the autoinducer *N*-hexanoyl homoserine lactone in the presence and absence of 1/4 MIC of tested drugs were transferred to the wells of microtiter plates and mixed with 100 μL of *C. violaceum* suspensions (1 at O.D600). The plates were incubated for 16 h at room temperature and then completely dried at 60 °C. One hundred μL dimethyl sulfoxide (DMSO) were added to elute violacein pigment by incubation with shaking at 30 °C. The violacein in presence of tested drugs was quantified by measuring the absorbance at 590 nm and was evaluated as a percentage change from untreated cultures (negative control). 

### 4.7. Assay of Biofilm Formation

The tested *β*-blockers’ abilities to inhibit biofilm formation were assessed as described before [87]. Aliquots of 100 µL of *P. aeruginosa* or *S. typhimurium* (1 × 10^6^ CFU/mL) were added to the wells of microtiter plates in the presence or absence of tested drugs at sub-MIC. After incubation for 24 h at 37 °C, the non-adhered cells were washed out and the attached biofilm-forming cells were fixed with methanol and stained with 1% crystal violet for 20 min. Excess crystal violet was removed, washed and left to air dry. Then, attached crystal violet was eluted with glacial acetic acid (33%), and the absorbances were measured at 590 nm. The formation of biofilm was calculated for each drug as a percentage change from untreated bacterial cultures. 

To visualize the effect of tested drugs on the formation of biofilms, the biofilms were allowed to be developed on cove slips in the presence or absence of tested drugs as described before [18]. Briefly, cover slips were placed in Falcon tubes with TSB with or without tested drugs at sub-MIC, and inoculated with *P. aeruginosa* or *S. typhimurium* (1 × 10^6^ CFU/mL). After overnight incubation at 37 °C, the cover slips were washed to remove the non-adherent cells and the adhered biofilm-forming cells were fixed with methanol and stained with 1% crystal violet. Then, the coverslips were imaged under a light microscope (Leica DM750 HD digital microscope, Mannheim, Germany).

### 4.8. Quantitative RT-PCR of P. aeruginosa QS-Encoding Genes and S. typhimurium Sensor Kinase Encoding Genes

To approve the inhibitory activity of tested *β*-blockers against QS and virulence of *P. aeruginosa* and *S. typhimurium*, a quantitative real-time PCR was performed. The RNA of *β*-blocker treated and untreated *P. aeruginosa* or *S. typhimurium* cultures were extracted by the Purification Kit Gene JET RNA (Thermoscientific, Waltham, MA, USA) according to the manufacturer’s protocol, and the extracted RNA were stored at −80 °C until use [39]. 

The expression of *P. aeruginosa* QS-encoding genes (*rhlI*, *rhlR, lasI, lasR*, *pqsA* and *pqsR*) were evaluated in the presence and absence of tested drugs at sub-MIC by qRT-PCR. The relative expression levels were normalized to the expression level of the housekeeping gene *ropD*. Furthermore, the expression levels of sensor kinase encoding genes *qseC* and *qseE* in *S. typhimurium* were quantified in the presence and absence of tested *β*-blockers at sub-MIC and normalized to the expression of endogenous control gene *gyrB*. (The primers used in this study are listed in Supplementary Data, Table S7). Untreated bacteria and bacteria treated with sub-MIC of tested drugs, were employed for cDNA synthesis by reverse transcription using a high-capacity cDNA reverse transcriptase kit (Applied Biosystem, Waltham, MA, USA).Then, the Syber Green I PCR Master Kit (Fermentas, Waltham, MA, USA) was used to amplify the cDNA in a multi-well plate using the Step One instrument (Applied Biosystem, Waltham, MA, USA).To attest the specific PCR amplification, agarose gel electrophoresis and a melting curve analysis of products were used according to the recommendation of the manufacturer. The relative genes’ expressions were calculated by the comparative threshold cycle (∆∆Ct) method [88].

### 4.9. In-Vivo Mice Protection Assay

In order to evaluate the in vivo anti-Qs and anti-virulence activities of *β*-blockers; the mice survival model was employed to assess the in vivo protective activity of metoprolol against *P. aeruginosa* or *S. typhimurium* pathogenesis as previously described [17]. Briefly, fresh *P. aeruginosa or S. typhimurium* overnight cultures in LB broth containing or not metoprolol at sub-MIC, were adjusted to ≈1 × 10^6^ CFU/mL) in phosphate-buffered saline (PBS). For evaluating the protective activity of metoprolol against *P. aeruginosa*, female *Mus musculus* mice at three weeks old were divided into 4 groups (*n* = 10). Group one was intraperitoneally injected with 100 μL of metoprolol-treated *P. aeruginosa* in sterile PBS. The positive control group was intraperitoneally injected with untreated *P. aeruginosa*. Two negative controls were injected with sterile PBS or kept uninfected.

For evaluating the protective activity against *S. typhimurium*, four mice groups were recruited and each comprises ten mice. Group one was injected with *S. typhimurium* treated with metoprolol at sub-MIC. One positive and two negative mice groups were injected with untreated *S. typhimurium,* PBS or kept uninfected, respectively. The mice survival was observed over five days and plotted using the Kaplan–Meier method.

## 5. Conclusions

Bacterial resistance development to diverse antimicrobial agents is an emerging problem that mandates an efficient solution. Several approaches have been conducted, among the most promising ones is tackling bacterial virulence because of its numerous advantages. In the current study, a detailed in silico study was performed to evaluate the ability of 22 *β*-adrenergic blockers to compete on three different structurally Gram-negative QS receptors. The tested drugs showed diverse abilities to bind different QS receptors; however, atenolol, esmolol, and metoprolol showed the highest binding affinity to the different QS receptors. Atenolol, esmolol, and metoprolol as representative for *β*-adrenergic blockers were selected for further in vitro and in vivo investigations. Atenolol, esmolol, and metoprolol significantly in vitro diminished the QS-controlled virulence in *Chromobacterium violaceum*, *Pseudomonas aeruginosa,* and *Salmonella typhimurium*. Furthermore, the three tested drugs down-regulated the QS-encoding genes in *P. aeruginosa* and sensor kinase encoding genes that sense adrenergic hormones on the surface of *S. typhimurium*. Interestingly, metoprolol was selected to be tested in vivo where it protected mice from *P. aeruginosa* and *S. typhimurium* pathogenesis. *β*-adrenergic blockers are promising anti-virulence agents, hindering bacterial QS systems, decreasing the adrenergic hormones induced virulence, and diminishing the bacterial espionage on host cells.

## Figures and Tables

**Table 1:** Docking energies of investigated adrenoreceptor blockers and co-crystallized reference controls at the binding sites of three bacterial LuxR-type quorum sensing transcription factors from *A. tumefaciens* (TraR; PDB entry: 1L3L), *P. aeruginosa* (QscR; PDB entry: 3SZT), and *C. violaceum* (CviR; PDB entry: 3QP5) throughout the preliminary docking analysis.

Compound	Name	Binding Energy (Kcal/mol) ^a^		
		1L3L	3SZT	3QP5
**1**	Propranolol	**−6.9518**	−6.8120	−6.9592
**2**	Pindolol	**−6.7337**	−6.7648	**−7.2291**
**3**	Levobunolol	−5.8300	−5.3392	−5.2812
**4**	Nadolol	−5.3036	−6.6720	−6.0193
**5**	Oxprenolol	−5.5981	−6.7365	−6.7374
**6**	Carteolol	−5.3073	−6.7183	−7.0493
**7**	Penbutolol	−4.0056	−5.2514	−5.1763
**8**	Timolol	**−6.8266**	−6.9061	−6.6453
**9**	Sotalol	−6.1145	−6.3450	−6.8093
**10**	Atenolol	**−7.9923**	**−8.2452**	**−8.2351**
**11**	Esmolol	**−6.9957**	**−8.2803**	**−7.4849**
**12**	Betaxolol	−5.0198	**−7.6193**	**−7.7426**
**13**	Bisoprolol	−6.1495	**−9.2750**	**−7.3777**
**14**	Metoprolol	**−7.2391**	**−8.7438**	**−7.8018**
**15**	Practolol	−5.6051	−6.2557	−6.9717
**16**	Metipranolol	−4.0760	−5.8692	−6.7320
**17**	Acebutolol	−5.7674	**−8.4568**	**−8.4732**
**18**	Celiprolol	−3.8698	−5.1621	−5.3801
**19**	Bucindolol	−2.2153	−5.8371	−6.2381
**20**	Carvedilol	−1.8936	−3.8607	−4.2513
**21**	Labetalol	−4.7226	**−7.7411**	**−7.8677**
**22**	Nebivolol	−1.9527	−5.2294	−4.8969
**1L3L Reference**	**O-C8-HSL**	−6.2977	–	–
**3SZT Reference**	**O-C12-HSL**	–	−7.5547	–
**3QP5 Reference**	**HLC**	−6.6245	−7.6488	−7.2051

^a^ Docking binding energy depicted at more negative values as compared to those of control reference ligands are highlighted and shown in bold numbers.

**Table 2:** Parameters of ligand–protein binding interactions at the canonical binding site of TraR *A. tumefaciens* (PDB entry: 1L3L) during the directed flexible receptor docking protocol.

Compound	Docking Energy (Kcal/mol) ^a^		H-Bond Interactions	Hydrophobic Interactions	π-Interactions	Van Der Waal with Side Chain Carbons
	Preliminary (Rigid)	Induced-Fit (Flexible)				
**Propranolol**	−6.9518	−7.5013	Tyr53, Asp70, Thr129	Ala38, Leu40, Ala49, Tyr53, Trp57, Phe62, Val72, Trp85, Phe101, Tyr102, Ala105, Ile110, Met127	Tyr61 (π-π)	Gln58 (C*β*, C*δ*)
**Pindolol**	−6.7337	−7.4013	Gln58, Tyr61, Asp70	Ala38, Leu40, Ala49, Tyr53, Trp57, Phe62, Val72, Val73, Trp85, Phe101, Tyr102, Ile110, Met127	Tyr61 (π-π)	-
**Timolol**	−6.8266	−7.4812	Tyr53, Trp57, Asp70, Thr129	Ala38, Leu40, Ala49, Tyr53, Trp57, Val72, Trp85, Phe101, Tyr102, Ala105, Ile110, Met127	Tyr61 (H-π)	-
**Atenolol**	−7.9923	−8.5142	Tyr53, Gln85, Tyr61, Phe62, Asp70, Thr129	Ala38, Leu40, Ala49, Tyr53, Trp57, Val72, Val73, Trp85, Phe101, Tyr102, Ala105, Ile110, Met127	Tyr61 (π-π)	-
**Esmolol**	−6.9957	−7.5913	Thr51, Tyr53 *, Phe62, Asp70, Thr129	Ala38, Leu40, Ala49, Tyr53, Trp57, Val72, Trp85, Phe101, Tyr102, Ala105, Ile110, Met127	Tyr61 (π-π)	-
**Metoprolol**	−7.2391	−8.1923	Thr51, Tyr53, Asp70, Trp85, Thr129	Ala38, Leu40, Ala49, Tyr53, Trp57, Val72, Val73, Trp85, Phe101, Tyr102, Ala105, Ile110, Met127	Tyr61 (π-π)	-
**HLC**	−6.6245	−7.1612	Tyr53, Trp57, Asp70, Tyr102	Ala38, Leu40, Ala49, Tyr53, Trp57, Val72, Trp85, Phe101, Tyr102, Ala105, Ile110, Met127	Tyr61 (π-π)	Gln58 (C*β*, C*δ*)

^a^ MOE docking energies; Docking scores utilizing the scoring function assigned for the best-ranking poses following refinement through the GBVI/WSA_dG forcefield rescoring function being incorporated within the MOE package; * indicates ligand’s multiple polar interactions with the designated amino acids.

**Table 3:** Parameters of ligand–protein binding interactions at the canonical binding site of QscR *P. aeruginosa* (PDB entry: 3SZT) during the directed flexible receptor docking protocol.

Compound	Docking Energy (Kcal/mol) ^a^		H-Bond Interactions	Hydrophobic Interactions	π-Interactions	Van Der Waal with Side Chain Carbons
	Preliminary (Rigid)	Induced-Fit (Flexible)				
**Atenolol**	−8.2452	−8.9102	Ser38, Tyr52, Tyr58, Tyr66, Asp75	Ala41, Tyr52, Tyr58, Trp62, Tyr66, Ile77, Val78, Leu82, Phe101, Trp102, Ala105, Ile110, Ile125, Met127, Val131	Trp90 (π-H) Trp102 (π-H)	Arg42 (C*β*)
**Esmolol**	−8.2803	−8.9182	Ser38, Arg42, Tyr58, Trp66, Ser129, Asp75	Phe39, Ala41, Tyr52, His53, Phe54, Tyr58, Trp62, Pro76, Ile77, Val78, Leu82, Trp90, Phe101, Trp102, Ala105, Ile110, Pro117, Ile125, Met127, Val131	Phe54 (π-π) Trp90 (π-H)	-
**Betaxolol**	−7.6193	−8.2918	Ser38, Tyr58 *, Trp66, Asp75, Met127	Phe39, Ala41, Tyr52, His53, Tyr58, Trp62, Ile77, Val78, Leu82, Trp90, Phe101, Trp102, Ala105, Ile110, Pro117, Ile125, Met127, Val131	Phe54 (π-π) Trp90 (π-H)	-
**Bisoprolol**	−9.2750	−9.7616	Ser38 *, Tyr58, Trp90, Asp75 *, Leu82, Ser129	Phe39, Ala41, Tyr52, His53, Tyr58, Trp62, Ile77, Val78, Leu82, Trp90, Phe101, Trp102, Ile110, Pro117, Ile125, Met127, Val131	Phe54 (π-π) Trp90 (π-H) Tyr66 (π-H)	Arg42 (C*β*, C*δ*)
**Metoprolol**	−8.7438	−9.5953	Ser38, Arg42, Tyr52, Tyr58 *, Asp75, Ser129	Phe39, Ala41, Tyr52, Tyr58, Trp62, Ile77, Val78, Leu82, Trp90, Phe101, Trp102, Ala105, Ile110, Ile125, Met127, Val131	Phe54 (π-π) Trp90 (π-H) Trp102 (π-H)	Arg42 (C*β*, C*δ*)
**Acebutolol**	−8.4568	−9.3164	Ser38 *, Tyr58, Thr72, Asp75 *, Met127, Ser129	Phe39, Ala41, Tyr52, His53, Tyr58, Trp62, Ile77, Val78, Leu82, Trp90, Phe101, Trp102, Ile110, Pro117, Ile125, Met127, Val131	Phe54 (π-π) Tyr66 (π-H) Trp90 (π-H)	Arg42 (C*β*, C*δ*)
**Labetalol**	−7.7411	−8.7880	Ser38, Tyr58, Trp62, Trp90	Phe39, Ala41, Tyr52, His53, Phe54, Tyr58, Trp62, Ile77, Val78, Leu82, Phe101, Trp102, Ala105, Ile110, Ile125, Met127	Tyr52 (π-π) Phe54 (π-H) Tyr58 (π-H) Tyr66 (π-H)	Arg42 (C*β*)
**HLC**	−7.6488	−7.9912	Ser38, Tyr58, Trp62, Tyr66, Asp75	Phe39, Ala41, Tyr52, Tyr58, Trp62, Ile77, Val78, Phe101, Trp102, Ala105, Ile110, Ile125, Met127	Phe54 (π-π) Trp90 (π-H)	Arg42 (C*β*)

^a^ MOE docking energies; Docking scores utilizing the scoring function assigned for the best-ranking poses following refinement through the GBVI/WSA_dG forcefield rescoring function being incorporated within the MOE package; * indicates the ligand’s multiple polar interactions with the designated amino acids.

**Table 4:** Parameters of ligand–protein binding interactions at the canonical binding site of CviR *C. violaceum* (PDB entry: 3QP5) during the directed flexible receptor docking protocol.

Compound	Docking Energy (Kcal/mol) ^a^		H-Bond Interactions	Hydrophobic Interactions	π-Interactions	Van Der Waal with Side Chain Carbons
	Preliminary (Rigid)	Induced-Fit (Flexible)				
**Pindolol**	−7.2291	−8.0192	Tyr80 *, Asp97 *, Ser155	Leu57, Leu72, Trp84, Leu85, Ala94, Ile99, Leu100, Phe115, Phe126, Ala130, Met135, Ile153	Tyr80 (π-π) Tyr88 (π-π) Trp111 (π-H)	-
**Atenolol**	−8.2351	−8.9128	Tyr80, Met89, Asp97 *, Ser155, Met253	Leu57, Leu72, Val75, Trp84, Leu85, Tyr88, Met89, Ala94, Ile99, Leu100, Phe115, Phe126, Ala130, Met135, Ile153, Val250, Met253	Tyr80 (π-π) Trp111 (π-H)	-
**Esmolol**	−7.4849	−8.2830	Tyr80, Asp97 *, Ser155	Leu57, Leu72, Val75, Tyr80, Trp84, Leu85, Met89, Ala94, Ile99, Leu100, Phe115, Phe126, Ala130, Met135, Ile153, Val250, Met253	Tyr88 (π-π) Trp111 (π-H)	-
**Betaxolol**	−7.3777	−8.1034	Tyr80, Asp97 *	Leu57, Leu72, Val75, Tyr80, Trp84, Leu85, Met89, Ala94, Ile99, Leu100, Phe115, Phe126, Ala130, Met135, Ile153, Val250, Met253	Tyr88 (π-π) Trp111 (π-H)	-
**Bisoprolol**	−7.8018	−8.8064	Tyr80, Asp97 *, Ser155	Leu57, Leu72, Val75, Tyr80, Trp84, Leu85, Met89, Ala94, Ile99, Leu100, Phe115, Phe126, Ala130, Met135, Ile153, Val250, Met253	Tyr88 (π-π) Trp111 (π-H)	-
**Metoprolol**	−7.7426	−8.7912	Tyr80, Asp97 *, Ser155	Leu57, Leu72, Val75, Tyr80, Trp84, Leu85, Met89, Ala94, Ile99, Leu100, Phe115, Phe126, Ala130, Met135, Ile153, Val250, Met253	Tyr88 (π-π) Trp111 (π-H)	-
**Acebutolol**	−8.4732	−9.0849	Tyr80, Trp84, Tyr88, Asp97 *	Leu57, Ala59, Leu72, Val75, Trp84, Leu85, Met89, Ala94, Ile99, Leu100, Phe115, Phe126, Ala130, Met135, Ile153, Val250, Met253	Tyr80 (π-π) Tyr88 (π-π) Trp111 (π-H)	-
**Labetalol**	−7.8677	−8.8048	Tyr80, Trp84, Asp97 *, Met135	Leu57, Ala59, Leu72, Val75, Tyr80, Trp84, Leu85, Met89, Ala94, Pro98, Ile99, Leu100, Phe115, Phe126, Ala130, Met135, Ile153, Val250, Met253	Tyr88 (π-H) Trp111 (π-π)	-
**HLC**	−7.2051	−8.08374	Tyr80, Trp84 *, Asp97	Leu57, Leu72, Val75, Trp84, Leu85, Met89, Ala94, Ile99, Leu100, Phe115, Phe126, Ala130, Met135, Ile153, Val250, Met253	Tyr80 (π-H) Tyr88 (π-π) Trp111 (π-H)	-

^a^ MOE docking energies; Docking scores utilizing the scoring function assigned for the best-ranking poses following refinement through the GBVI/WSA_dG forcefield rescoring function being incorporated within the MOE package; * indicates the ligand’s multiple polar interactions with the designated amino acids.

**Table 5:** Total binding free energies and individual energy term (Δ*G*_Total binding_) concerning the promising *β*-blockers and reference ligand at TraR *A. tumefaciens* protein binding site.

Energy (kJ/mol ± SD)	Ligand–Protein Complex						
	HLC	Comp.1	Comp.2	Comp.8	Comp.10	Comp.11	Comp.14
**Δ*G*_van der Waals_**	−122.79 ± 14.13	−140.41 ± 3.50	−145.10 ± 10.69	−141.05 ± 25.17	−154.95 ± 28.56	−178.84 ± 11.78	−157.27 ± 15.93
**Δ*G*_Electrostatic_**	−46.75 ± 2.55	−39.64 ± 2.46	−47.33 ± 2.69	−42.92 ± 3.70	−52.74 ± 8.07	−47.20 ± 3.46	−36.65 ± 9.51
**Δ*G*_Solvation; Polar_**	120.42 ± 1.28	112.99 ± 5.10	135.90 ± 12.55	132.66 ± 16.36	155.59 ± 9.55	156.12 ± 5.68	122.03 ± 20.69
**Δ*G*_Solvation; non-polar; SASA_**	−18.75 ± 0.04	−17.26 ± 0.19	−16.34 ± 0.39	−18.21 ± 0.37	−18.21 ± 0.04	−19.79 ± 0.93	−17.17 ± 0.05
**Δ*G*_Total binding_**	−67.87 ± 10.34	−84.32 ± 0.66	−72.87 ± 4.16	−69.52 ± 12.88	−70.31 ± 27.13	−89.71 ± 3.56	−89.05 ± 4.71

**Table 6:** Total binding free energies and individual energy term (Δ*G*_Total binding_) concerning the promising *β*-blockers and reference ligand at QscR *P. aeruginosa* protein binding site.

Energy (kJ/mol ± SD)	Ligand–Protein Complex							
	HLC	Comp.10	Comp.11	Comp.12	Comp.13	Comp.14	Comp.17	Comp.21
**Δ*G*_van der Waals_**	−156.93 ± 22.35	−186.11 ± 6.21	−215.32 ± 5.77	−202.48 ± 6.06	−188.95 ± 0.89	−219.53 ± 1.04	−194.57 ± 5.13	−218.16 ± 8.55
**Δ*G*_Electrostatic_**	−67.65 ± 16.73	−109.52 ± 7.45	−71.01 ± 9.23	−56.97 ± 1.48	−48.43 ± 0.51	−58.31 ± 6.72	−54.54 ± 0.91	−46.50 ± 6.78
**Δ*G*_Solvation; Polar_**	162.60 ± 5.19	195.45 ± 7.07	188.52 ± 1.51	170.69 ± 7.56	160.40 ± 6.81	185.98 ± 8.38	185.73 ± 8.25	192.86 ± 7.42
**Δ*G*_Solvation; non-polar; SASA_**	−17.52 ± 0.71	−17.45 ± 0.29	−20.66 ± 0.42	−20.28 ± 0.20	−19.34 ± 0.11	−22.53 ± 0.18	−18.87 ± 0.64	−21.20 ± 0.13
**Δ*G*_Total binding_**	−79.50 ± 1.14	−117.63 ± 6.12	−118.47 ± 13.07	−109.04 ± 2.77	−96.33 ± 7.31	−114.40 ± 13.87	−82.25 ± 1.57	−93.00 ± 9.32

**Table 7:** Total binding free energies and individual energy term (Δ*G*_Total binding_) concerning the promising *β*-blockers and reference ligand at CviR *C. violaceum* protein binding site.

Energy (kJ/mol ± SD)	Ligand–Protein Complex								
	HLC	Comp.2	Comp.10	Comp.11	Comp.12	Comp.13	Comp.14	Comp.17	Comp.21
**Δ*G*_van der Waals_**	−120.62 ± 8.69	−173.26 ± 2.38	−170.77 ± 4.23	−181.41 ± 4.10	−147.37 ± 23.03	−171.40 ± 29.61	−182.07 ± 9.12	−184.75 ± 15.74	−168.13 ± 6.62
**Δ*G*_Electrostatic_**	−52.81 ± 15.96	−31.78 ± 8.88	−99.14 ± 7.53	−60.14 ± 4.73	−31.87 ± 9.69	−35.48 ± 12.10	−39.67 ± 1.36	−39.57 ± 22.05	−39.02 ± 16.73
**Δ*G*_Solvation; Polar_**	120.50 ± 15.88	149.10 ± 1.64	176.95 ± 9.60	157.83 ± 4.39	126.96 ± 13.52	142.19 ± 47.71	144.95 ± 2.54	144.14 ± 34.16	150.94 ± 2.65
**Δ*G*_Solvation; non-polar; SASA_**	−18.00 ± 0.27	−17.06 ± 0.21	−18.11 ± 0.27	−20.92 ± 1.04	−18.83 ± 0.70	−20.96 ± 1.82	−21.72 ± 0.24	−21.28 ± 0.77	−20.30 ± 0.63
**Δ*G*_Total binding_**	−70.93 ± 9.05	−73.00 ± 9.41	−111.07 ± 6.04	−104.64 ± 4.79	−71.10 ± 19.89	−85.66 ± 4.18	−98.51 ± 13.26	−101.46 ± 4.40	−76.51 ± 21.33

## Data Availability

Data is contained within the article or supplementary material.

## Supplementary Materials

The following are available online at https://www.mdpi.com/article/10.3390/ph15020110/s1, Figure S1: Three-dimensional representation of the binding site topology at the three bacterial LuxR-type quorum sensing transcription factors; Figure S2: Conformational analysis of simulated ligand–TraR *A. tumefaciens* protein complexes; Figure S3: Conformational analysis of simulated ligand–QscR *P. aeruginosa* protein complexes; Figure S4: Conformational analysis of simulated ligand–CviR *C. violaceum* protein complexes; Figure S5: The architecture of three bacterial LuxR-type quorum sensing transcription factors; Figure S6: Superimposing the co-crystallized (magenta sticks) and redocked (yellow sticks) ligands; Table S1: Descriptive ligand–TraR *A. tumefaciens* binding interactions through directed flexible docking protocol; Table S2: Descriptive ligand–QscR *P. aeruginosa* binding interactions through directed flexible docking protocol; Table S3: Descriptive ligand–CviR *C. violaceum* binding interactions through directed flexible docking protocol; Table S4: Estimated ΔRMSF values for ligand–TraR *A. tumefaciens* proteins along the whole MD simulation; Table S5: Estimated ΔRMSF values for ligand–QscR *P. aeruginosa* proteins along the whole MD simulation; Table S6: Estimated ΔRMSF values for ligand–CviR *C. violaceum* proteins along the whole MD simulation; Table S7: Sequences of the used primers in this study.
