# Supplementary material for: Computational and Biological Evaluation of β-Adrenoreceptor Blockers as Promising Bacterial Anti-Virulence Agents
Source: Pharmaceuticals (Basel). 2022 Jan 18;15(2):110. doi: 10.3390/ph15020110 (PMC8877484; doi:10.3390/ph15020110)
Supplement: Supplementary file 1 [file pharmaceuticals-15-00110-s001.zip › pharmaceuticals-1535719-supplementary.pdf]

Computational and Biological Evaluation of β-adrenoreceptor Blockers as Promising Bacterial Anti-Virulence Agents

Ahmad J. Almalki [1,*], Tarek S. Ibrahim [1], Sameh S. Elhady [2], Wael A. H. Hegazy [3] and Khaled M. Darwish [4,*]

[1] Department of Pharmaceutical Chemistry, Faculty of Pharmacy, King Abdulaziz University, Jeddah, 21589, Saudi Arabia; tmabrahem@kau.edu.sa
[2] Department of Natural Products, Faculty of Pharmacy, King Abdulaziz University, Jeddah 21589, Saudi Arabia; ssahmed@kau.edu.sa
[3] Department of Microbiology and Immunology, Faculty of Pharmacy, Zagazig University, Zagazig, 44519, Egypt; waelmhegazy@daad-alumni.de
[4] Medicinal Chemistry Department, Faculty of Pharmacy, Suez Canal University, Ismailia 41522, Egypt
* Correspondence: ajalmalki@kau.edu.sa (A.J.A.); khaled_darwish@pharm.suez.edu.eg (K.M.D.)

Additional Experimental Detail

1) **Table S1.** Descriptive ligand-TraR *A. tumefaciens* binding interactions through directed flexible docking protocol.
2) **Table S2.** Descriptive ligand-QscR *P. aeruginosa* binding interactions through directed flexible docking protocol.
3) **Table S3.** Descriptive ligand-CviR *C. violaceum* binding interactions through directed flexible docking protocol.
4) **Table S4.** Estimated ΔRMSF values for ligand-TraR *A. tumefaciens* proteins along the whole MD simulation.
5) **Table S5.** Estimated ΔRMSF values for ligand-QscR *P. aeruginosa* proteins along the whole MD simulation.
6) **Table S6.** Estimated ΔRMSF values for ligand-CviR *C. violaceum* proteins along the whole MD simulation.
7) **Table S7.** Sequences of the used primers in this study
8) **Figure S1.** 3D-representation of the binding site topology at the three bacterial LuxR-type quorum-sensing transcription factors.
9) **Figure S2.** Conformational analysis of simulated ligand-TraR *A. tumefaciens* protein complexes.
10) **Figure S3.** Conformational analysis of simulated ligand-QscR *P. aeruginosa* protein complexes.
11) **Figure S4.** Conformational analysis of simulated ligand-CviR *C. violaceum* protein complexes.
12) **Figure S5.** The architecture of three bacterial LuxR-type quorum sensing transcription factors.
13) **Figure S6.** Superimposing the co-crystallized (magenta sticks) and redocked (yellow sticks) ligands

Table S1. Descriptive ligand-TraR *A. tumefaciens* binding interactions through directed flexible docking protocol

Compound	Ligand-target interaction description<br>[Type; Length (Å); Angle (°); Binding Residues]
**Propranolol**	Polar ; 2.8 **Å** ; Asp70 (sidechain C**O**⁻ with **N**⁺H₂)<br>H-bond ; 3.1 **Å** ; 121 ° ; Tyr53 (sidechain **O**H with N⁺**H**H)<br>H-bond ; 1.9 **Å** ; 148 ° ; Thr129 (sidechain **O**H with propanol-O**H**)<br>π-π interaction ; 2.9 **Å** ; Tyr61<br>van der Waal ; 3.9 **Å** and 4.4 **Å;** Gln58 sidechain Cβ and Cδ
**Pindolol**	Polar ; 2.7 **Å** ; Asp70 (sidechain C**O**⁻ with **N**⁺H₂)<br>H-bond ; 3.2 **Å** ; 159 ° ; Gln58 (mainchain C=**O** with indole N**H**)<br>H-bond ; 2.6 **Å** ; 127 ° ; Tyr61 (sidechain **O**H with propanol-O**H**)<br>π-π interaction ; 3.2 **Å** ; Tyr61
**Timolol**	Polar ; 3.3 **Å** ; Asp70 (sidechain C**O**⁻ with **N**⁺H₂)<br>H-bond ; 2.0 **Å** ; 140 ° ; Tyr53 (sidechain O**H** with propanol-**O**H)<br>H-bond ; 3.1 **Å** ; 155 ° ; Trp57 (sidechain N**H** with aryloxy-**O**)<br>H-bond ; 2.9 **Å** ; 167 ° ; Thr129 (sidechain O**H** with ring **S**)<br>π-H interaction ; 4.1 **Å** ; Tyr61
**Atenolol**	Polar ; 3.0 **Å** ; Asp70 (sidechain C**O**⁻ with **N**⁺H₂)<br>H-bond ; 1.8 **Å** ; 176 ° ; Tyr53 (sidechain O**H** with propanol-**O**H)<br>H-bond ; 2.9 **Å** ; 125 ° ; Gln58 (sidechain C=**O** with amide N**H**)<br>H-bond ; 3.1 **Å** ; 123 ° ; Tyr61 (sidechain O**H** with aryloxy-**O**)<br>H-bond ; 3.2 **Å** ; 135 ° ; Phe62 (mainchain N**H** with amide C=**O**)<br>H-bond ; 2.8 **Å** ; 121 ° ; Thr129 (sidechain O**H** with propanol-**O**H)<br>π-π interaction ; 3.1 **Å** ; Tyr61
**Esmolol**	Polar ; 2.8 **Å** ; Asp70 (sidechain C**O**⁻ with **N**⁺H₂)<br>H-bond ; 2.3 **Å** ; 174 ° ; Tyr53 (sidechain O**H** with aryloxy-**O**)<br>H-bond ; 3.2 **Å** ; 131 ° ; Tyr53 (mainchain N**H** with ester C=**O**)<br>H-bond ; 3.1 **Å** ; 121 ° ; Thr51 (sidechain O**H** with ester C=**O**)<br>H-bond ; 3.0 **Å** ; 125 ° ; Phe62 (mainchain N**H** with ester **O**CH₃)<br>H-bond ; 2.8 **Å** ; 131 ° ; Thr129 (sidechain O**H** with aryloxy-**O**)<br>π-π interaction ; 4.0 **Å** ; Tyr61
**Metoprolol**	Polar ; 2.8 **Å** ; Asp70 (sidechain C**O**⁻ with **N**⁺H₂)<br>H-bond ; 2.9 **Å** ; 170 ° ; Tyr53 (sidechain O**H** with aryloxy-**O**)<br>H-bond ; 3.0 **Å** ; 128 ° ; Thr51 (sidechain C=**O** with amide N**H**)<br>H-bond ; 2.9 **Å** ; 120 ° ; Trp85 (mainchain N**H** with propanol-**O**H)<br>H-bond ; 2.7 **Å** ; 147 ° ; Thr129 (sidechain O**H** with terminal methoxy **O**CH₃)<br>π-π interaction ; 3.2 **Å** ; Tyr61
**HLC**	H-bond ; 3.2 **Å** ; 127 ° ; Asp70 (sidechain C=**O** with N**H**)<br>H-bond ; 2.1 **Å** ; 137 ° ; Tyr53 (sidechain O**H** with amide C=**O**)<br>H-bond ; 3.3 **Å** ; 153 ° ; Trp57 (sidechain N**H** with aryloxy-**O**)<br>H-bond ; 3.1 **Å** ; 120 ° ; Trp102 (sidechain O**H** with lactone C=**O**)<br>π-π interaction ; 4.1 **Å** ; Tyr61<br>van der Waal ; 3.5 **Å** and 4.8 **Å;** Gln58 sidechain Cβ and Cδ

Table S2. Descriptive ligand-QscR *P. aeruginosa* binding interactions through directed flexible docking protocol

Compound	Ligand-target interaction description<br>[Type; Length (Å); Angle (°); Binding Residues]
**Atenolol**	Polar ; 3.1 **Å** ; Asp75 (sidechain C**O**$^-$ with **N**$^+$H$_2$)<br>H-bond ; 2.6 **Å** ; 129 ° ; Ser38 (sidechain O**H** with aryloxy-**O**)<br>H-bond ; 3.3 **Å** ; 153 ° ; Tyr52 (sidechain **O**H with amide NH**H**)<br>H-bond ; 2.4 **Å** ; 154 ° ; Tyr58 (sidechain O**H** with propanol-**O**H)<br>H-bond ; 3.3 **Å** ; 137 ° ; Tyr66 (sidechain **O**H with propanol-O**H**)<br>π-H interaction ; 3.0 **Å** ; Trp90<br>π-H interaction ; 3.3 **Å** ; Trp102<br>van der Waals ; 3.6 **Å;** Arg42 sidechain Cβ
**Esmolol**	Polar ; 3.0 **Å** ; Asp75 (sidechain C**O**$^-$ with **N**$^+$H$_2$)<br>H-bond ; 2.1 **Å** ; 121 ° ; Ser38 (sidechain O**H** with propanol-**O**H)<br>H-bond ; 2.6 **Å** ; 132 ° ; Arg42 (mainchain N**H** with ester C=**O**)<br>H-bond ; 1.8 **Å** ; 147 ° ; Tyr58 (sidechain O**H** with propanol-**O**H)<br>H-bond ; 2.5 **Å** ; 129 ° ; Tyr66 (sidechain O**H** with aryloxy-**O**)<br>H-bond ; 2.7 **Å** ; 123 ° ; Ser129 (sidechain **O**H with propanol-O**H**)<br>π-π interaction ; 4.7 **Å** ; Phe54<br>π-H interaction ; 3.2 **Å** ; Trp90
**Betaxolol**	Polar ; 3.3 **Å** ; Asp75 (sidechain C**O**$^-$ with **N**$^+$H$_2$)<br>H-bond ; 1.9 **Å** ; 137 ° ; Ser38 (sidechain O**H** with propanol-**O**H)<br>H-bond ; 1.7 **Å** ; 170 ° ; Tyr58 (sidechain O**H** with propanol-**O**H)<br>H-bond ; 2.2 **Å** ; 159 ° ; Tyr58 (sidechain **O**H with N$^+$**H**H)<br>H-bond ; 3.2 **Å** ; 129 ° ; Tyr66 (sidechain O**H** with aryloxy-**O**)<br>H-bond ; 3.2 **Å** ; 128 ° ; Met127 (mainchain N**H** with terminal-**O**-linker)<br>π-π interaction ; 4.5 **Å** ; Phe54<br>π-H interaction ; 3.4 **Å** ; Trp90
**Bisoprolol**	Polar ; 3.3 **Å** ; Asp75 (sidechain C**O**$^-$ with **N**$^+$H$_2$)<br>H-bond ; 2.5 **Å** ; 123 ° ; Ser38 (sidechain O**H** with aryloxy-**O**)<br>H-bond ; 2.6 **Å** ; 148 ° ; Ser38 (sidechain **O**H with propanol-O**H**)<br>H-bond ; 1.9 **Å** ; 120 ° ; Tyr58 (sidechain O**H** with propanol-**O**H)<br>H-bond ; 2.1 **Å** ; 124 ° ; Asp75 (sidechain C=**O** with N$^+$**H**H)<br>H-bond ; 3.3 **Å** ; 121 ° ; Trp90 (sidechain N**H** with propanol-**O**H)<br>H-bond ; 3.0 **Å** ; 128 ° ; Ser129 (sidechain O**H** with aryloxy-**O**)<br>π-π interaction ; 4.4 **Å** ; Phe54<br>π-H interaction ; 3.6 **Å** ; Tyr66<br>π-H interaction ; 2.9 **Å** ; Trp90<br>van der Waal ; 3.7 **Å** and 4.2 **Å** ; Arg42 sidechain Cβ and Cδ
**Metoprolol**	Polar ; 3.1 **Å** ; Asp75 (sidechain C**O**$^-$ with **N**$^+$H$_2$)<br>H-bond ; 3.0 **Å** ; 137 ° ; Ser38 (sidechain **O**H with propanol-O**H**)<br>H-bond ; 3.3 **Å** ; 129 ° ; Arg42 (mainchain N**H** with terminal-**O**-linker)<br>H-bond ; 3.3 **Å** ; 124 ° ; Tyr52 (sidechain N**H** with propanol-**O**H)<br>H-bond ; 2.6 **Å** ; 170 ° ; Tyr58 (sidechain **O**H with N$^+$**H**H)<br>H-bond ; 2.0 **Å** ; 128 ° ; Tyr58 (sidechain O**H** with propanol-**O**H)<br>H-bond ; 3.0 **Å** ; 127 ° ; Ser129 (sidechain **O**H with propanol-O**H**)<br>π-π interaction ; 4.9 **Å** ; Phe54<br>π-H interaction ; 3.1 **Å** ; Trp90<br>π-H interaction ; 3.5 **Å** ; Trp102<br>van der Waal ; 3.0 **Å** and 4.0 **Å** ; Arg42 sidechain Cβ and Cδ
**Acebutolol**	Polar ; 3.2 **Å** ; Asp75 (sidechain C**O**$^-$ with **N**$^+$H$_2$)<br>H-bond ; 1.9 **Å** ; 128 ° ; Ser38 (sidechain O**H** with aryloxy-**O**)

	H-bond **;** 2.1 **Å ;** 127 ° **;** Ser38 (sidechain O**H** with propanol-**O**H)<br>H-bond **;** 1.7 **Å ;** 154 ° **;** Tyr58 (sidechain O**H** with propanol-**O**H)<br>H-bond **;** 3.0 **Å ;** 137 ° **;** Thr72 (mainchain N**H** with acetyl C=**O**)<br>H-bond **;** 1.8 **Å ;** 157 ° **;** Asp75 (sidechain C=**O** with N^+**H**H)<br>H-bond **;** 3.3 **Å ;** 124 ° **;** Met127 (mainchain N**H** with terminal amide C=**O**)<br>H-bond **;** 3.0 **Å ;** 137 ° **;** Ser129 (sidechain **O**H with propanol-O**H**)<br>π-π interaction ; 4.0 **Å ;** Phe54<br>π-H interaction ; 2.9 **Å ;** Tyr66<br>π-H interaction ; 2.9 **Å ;** Trp90<br>van der Waal ; 3.7 **Å** and 4.3 **Å ;** Arg42 sidechain Cβ and Cδ
**Labetalol**	H-bond **;** 1.8 **Å ;** 149 ° **;** Ser38 (sidechain O**H** with ethanol-**O**H)<br>H-bond **;** 3.3 **Å ;** 137 ° **;** Tyr58 (sidechain O**H** with ethanol-**O**H)<br>H-bond **;** 2.3 **Å ;** 123 ° **;** Trp62 (mainchain N**H** with carbamide C=**O**)<br>π-π interaction ; 3.3 **Å ;** Tyr52<br>π-H interaction ; 3.0 **Å ;** Phe54<br>π-H interaction ; 3.2 **Å ;** Tyr58<br>π-H interaction ; 2.5 **Å ;** Tyr66<br>van der Waal ; 3.3 **Å ;** Arg42 sidechain Cβ
**HLC**	H-bond **;** 2.8 **Å ;** 128 ° **;** Ser38 (sidechain O**H** with amide C=**O**)<br>H-bond **;** 2.1 **Å ;** 148 ° **;** Tyr58 (sidechain N**H** with aryloxy-**O**)<br>H-bond **;** 2.0 **Å ;** 147 ° **;** Trp62 (sidechain N**H** with lactone C=**O**)<br>H-bond **;** 2.6 **Å ;** 149 ° **;** Tyr66 (sidechain O**H** with amide C=**O**)<br>H-bond **;** 2.4 **Å ;** 129 ° **;** Asp75 (sidechain O**H** with amide C=**O**)<br>π-π interaction ; 4.5 **Å ;** Phe54<br>π-H interaction ; 2.7 **Å ;** Trp90<br>van der Waal ; 4.6 **Å ;** Arg42 sidechain Cβ

Table S3. Descriptive ligand-CviR *C. violaceum* binding interactions through directed flexible docking protocol

Compound	Ligand-target interaction description [Type; Length (Å); Angle (°); Binding Residues]
**Pindolol**	Polar ; 3.0 **Å** ; Asp97 (sidechain C**O**⁻ with **N**$^+$H$_2$)<br>H-bond ; 3.1 **Å** ; 141 ° ; Tyr80 (sidechain O**H** with aryloxy-**O**)<br>H-bond ; 3.3 **Å** ; 162 ° ; Tyr80 (sidechain O**H** with propanol-**O**H)<br>H-bond ; 3.2 **Å** ; 164 ° ; Asp97 (sidechain C=**O** with N$^+$**H**H)<br>H-bond ; 2.5 **Å** ; 125 ° ; Ser155 (sidechain **O**H with propanol-O**H**)<br>π-π interaction ; 4.2 **Å** ; Tyr80<br>π-π interaction ; 3.7 **Å** ; Tyr88<br>π-H interaction ; 3.4 **Å** ; Trp111
**Atenolol**	Polar ; 3.2 **Å** ; Asp97 (sidechain C**O**⁻ with **N**$^+$H$_2$)<br>H-bond ; 2.5 **Å** ; 163 ° ; Tyr80 (sidechain O**H** with propanol-**O**H)<br>H-bond ; 3.1 **Å** ; 144 ° ; Met89 (sidechain **S**CH$_3$ with terminal amide NH**H**)<br>H-bond ; 3.3 **Å** ; 128 ° ; Asp97 (sidechain C=**O** with N$^+$**H**H)<br>H-bond ; 2.5 **Å** ; 126 ° ; Ser155 (sidechain **O**H with propanol-O**H**)<br>H-bond ; 3.3 **Å** ; 125 ° ; Met253 (sidechain **S**CH$_3$ with terminal amide N**H**H)<br>π-π interaction ; 3.6 **Å** ; Tyr88<br>π-H interaction ; 2.9 **Å** ; Trp111
**Esmolol**	Polar ; 2.7 **Å** ; Asp97 (sidechain C**O**⁻ with **N**$^+$H$_2$)<br>H-bond ; 3.1 **Å** ; 171 ° ; Tyr80 (sidechain O**H** with propanol-**O**H)<br>H-bond ; 2.9 **Å** ; 174 ° ; Asp97 (sidechain C=**O** with N$^+$**H**H)<br>H-bond ; 2.7 **Å** ; 129 ° ; Ser155 (sidechain **O**H with propanol-O**H**)<br>π-π interaction ; 3.7 **Å** ; Tyr88<br>π-H interaction ; 3.2 **Å** ; Trp111
**Betaxolol**	Polar ; 3.1 **Å** ; Asp97 (sidechain C**O**⁻ with **N**$^+$H$_2$)<br>H-bond ; 3.3 **Å** ; 161 ° ; Tyr80 (sidechain O**H** with propanol-**O**H)<br>H-bond ; 3.2 **Å** ; 150 ° ; Asp97 (sidechain C=**O** with N$^+$**H**H)<br>H-bond ; 1.9 **Å** ; 149 ° ; Asp97 (sidechain C**O**⁻ with propanol-O**H**)<br>π-π interaction ; 3.9 **Å** ; Tyr88<br>π-H interaction ; 3.0 **Å** ; Trp111
**Bisoprolol**	Polar ; 2.7 **Å** ; Asp97 (sidechain C**O**⁻ with **N**$^+$H$_2$)<br>H-bond ; 3.2 **Å** ; 172 ° ; Tyr80 (sidechain O**H** with propanol-**O**H)<br>H-bond ; 3.1 **Å** ; 164 ° ; Asp97 (sidechain C=**O** with N$^+$**H**H)<br>H-bond ; 3.1 **Å** ; 126 ° ; Ser155 (sidechain **O**H with propanol-O**H**)<br>π-π interaction ; 4.3 **Å** ; Tyr88<br>π-H interaction ; 3.3 **Å** ; Trp111
**Metoprolol**	Polar ; 2.8 **Å** ; Asp97 (sidechain C**O**⁻ with **N**$^+$H$_2$)<br>H-bond ; 3.3 **Å** ; 162 ° ; Tyr80 (sidechain O**H** with propanol-**O**H)<br>H-bond ; 3.3 **Å** ; 158 ° ; Asp97 (sidechain C=**O** with N$^+$**H**H)<br>H-bond ; 2.2 **Å** ; 134 ° ; Ser155 (sidechain **O**H with propanol-O**H**)<br>π-π interaction ; 3.8 **Å** ; Tyr88<br>π-H interaction ; 3.0 **Å** ; Trp111
**Acebutolol**	Polar ; 3.3 **Å** ; Asp97 (sidechain C**O**⁻ with **N**$^+$H$_2$)<br>H-bond ; 3.2 **Å** ; 142 ° ; Tyr80 (sidechain O**H** with aryloxy-**O**)<br>H-bond ; 3.3 **Å** ; 165 ° ; Trp84 (sidechain N**H** with propanol-**O**H)<br>H-bond ; 2.9 **Å** ; 128 ° ; Tyr88 (sidechain O**H** with propanol-**O**H)<br>H-bond ; 1.6 **Å** ; 168 ° ; Asp97 (sidechain C=**O** with propanol-O**H**)<br>π-π interaction ; 4.5 **Å** ; Tyr80<br>π-π interaction ; 3.8 **Å** ; Tyr88<br>π-H interaction ; 3.2 **Å** ; Trp111
**Labetalol**	Polar ; 2.2 **Å** ; Asp97 (sidechain C**O**⁻ with **N**$^+$H$_2$)<br>H-bond ; 3.3 **Å** ; 162 ° ; Tyr80 (sidechain O**H** with ethanol-**O**H)

	H-bond **;** 2.4 **Å ;** 162 ° **;** Trp84 (sidechain N**H** with phenyl-4-**O**H)<br>H-bond **;** 2.1 **Å ;** 158 ° **;** Asp97 (sidechain C=**O** with N+**H**H)<br>H-bond **;** 3.2 **Å ;** 134 ° **;** Met135 (sidechain **S**CH_3 with phenyl-4-O**H**)<br>π-H interaction ; 3.8 **Å ;** Tyr88<br>π-π interaction ; 3.0 **Å ;** Trp111
**HLC**	H-bond **;** 2.5 **Å ;** 158 ° **;** Tyr80 (sidechain O**H** with amide C=**O**)<br>H-bond **;** 2.0 **Å ;** 169 ° **;** Trp84 (sidechain N**H** with lactone C=**O**)<br>H-bond **;** 2.8 **Å ;** 121 ° **;** Trp84 (sidechain N**H** with lactone **O**)<br>H-bond **;** 2.3 **Å ;** 161 ° **;** Asp97 (sidechain C=**O** with amide N**H**)<br>π-H interaction ; 4.0 **Å ;** Tyr80<br>π-π interaction ; 5.1 **Å ;** Tyr88<br>π-H interaction ; 2.8 **Å ;** Trp111

Table S4. Estimated ΔRMSF[a] values for ligand-TraR *A. tumefaciens* proteins along the whole MD simulation.

Pocket Residues	HLC	Comp.1	Comp.2	Comp.8	Comp.10	Comp.11	Comp.14
**Ala38**	-0.08	-0.29	-0.15	0.08	0.26	0.10	0.22
**Tyr39**	0.06	-0.1	0.03	0.21	**0.35**	0.16	0.27
**Leu40**	0.18	-0.03	0.10	0.1	**0.36**	0.05	0.20
**Thr51**	**0.53**	0.23	**0.39**	**0.71**	**0.92**	**0.67**	**0.73**
**Tyr53**	**0.31**	-0.18	0.01	**0.38**	**0.62**	0.24	**0.72**
**Trp57**	-0.23	-0.63	-0.64	-0.17	0.11	-0.39	-0.01
**Tyr61**	-0.59	-0.98	-1.07	-0.70	-0.56	-0.91	-0.61
**Phe62**	-1.04	-1.47	-1.61	-1.18	-0.97	-1.39	-1.15
**Asp70**	0.19	**0.32**	0.25	**0.37**	**0.50**	0.27	**0.51**
**Val72**	**0.61**	**0.60**	**0.45**	-0.26	**0.62**	**0.29**	**0.46**
**Trp85**	-0.26	-0.29	-0.14	-0.93	0.16	-0.22	-0.01
**Phe101**	**0.35**	**0.64**	**0.36**	**0.31**	**0.51**	**0.46**	**0.55**
**Tyr102**	**0.49**	**0.73**	**0.63**	**0.31**	**0.73**	**0.35**	**0.67**
**Ala105**	**1.27**	**1.39**	**1.40**	**1.25**	**1.60**	**1.36**	**1.56**
**Ile110**	**1.08**	**1.12**	**1.29**	**1.06**	**1.55**	**1.31**	**1.47**
**Thr115**	**1.48**	**1.53**	**1.55**	**1.36**	**1.69**	**1.55**	**1.58**
**Met127**	-0.09	-0.05	-0.06	-0.05	0.07	-0.10	-0.06
**Phe128**	-0.25	-0.25	-0.17	-0.06	0.08	-0.09	-0.05
**Thr129**	-0.38	-0.36	-0.28	-0.31	0.00	-0.16	-0.11

[a] Relative difference root-mean-square fluctuation (ΔRMSF) was estimated for each ligand-associated TraR protein relative to the apo/unliganded state. Residues showing significant immobility are with ΔRMSF > 0.30 Å cut-off are in bold red color and highlighted.

Table S5. Estimated ΔRMSF[a] values for ligand-QscR *P. aeruginosa* proteins along the whole MD simulation.

Pocket Residues	HLC	Comp.10	Comp.11	Comp.12	Comp.13	Comp.14	Comp.17	Comp.21
**Ser38**	0.18	0.08	**0.37**	**0.37**	0.21	0.12	**0.35**	0.27
**Phe39**	0.00	-0.10	**0.32**	**0.31**	0.15	0.08	0.18	0.19
**Gly40**	-0.05	-0.46	0.11	0.14	-0.13	-0.03	0.07	-0.11
**Ala41**	0.14	-0.04	0.27	**0.31**	0.17	0.14	0.26	0.17
**Arg42**	0.02	0.13	**0.34**	**0.34**	**0.32**	**0.30**	**0.33**	0.06
**Tyr52**	-0.64	-0.61	-0.33	-0.34	-0.25	-0.14	-0.24	-0.98
**His53**	-0.25	-0.44	-0.16	-0.18	-0.26	-0.22	-0.15	-0.59
**Phe54**	-0.44	-0.41	-0.20	-0.11	-0.36	-0.28	-0.21	-0.32
**Ser56**	-0.51	-0.25	0.02	-0.04	-0.14	-0.25	-0.08	-0.10
**Tyr58**	-0.10	-0.21	0.11	-0.12	-0.09	-0.31	0.13	-0.10
**Trp62**	-0.08	0.03	0.28	**0.32**	0.01	0.02	**0.44**	0.19
**Lys63**	-0.40	-0.42	-0.03	-0.13	-0.71	-0.22	0.08	-0.12
**Tyr66**	-0.77	-0.49	-0.20	-0.18	-0.81	-0.04	0.00	-0.30
**Ile67**	-1.19	-0.89	-0.43	-0.53	-1.49	-0.33	-0.19	-0.69
**Thr72**	-0.88	-1.01	-0.57	-0.64	-1.30	-0.37	-0.41	-0.54
**Asp75**	**0.31**	**0.43**	**0.31**	**0.37**	**0.47**	0.28	0.25	0.07
**Ile77**	**0.31**	0.13	**0.45**	**0.43**	0.13	**0.32**	**0.40**	**0.41**
**Val78**	**0.37**	0.18	**0.59**	**0.54**	0.24	**0.46**	**0.51**	**0.47**
**Leu82**	**0.36**	**0.33**	**0.53**	**0.47**	0.19	**0.40**	**0.53**	0.07
**Trp90**	**0.34**	**0.37**	**0.43**	**0.47**	**0.35**	0.22	**0.45**	**0.49**
**Phe101**	-0.49	-0.20	-0.07	0.06	-0.12	-0.13	0.10	0.12
**Trp102**	-0.11	0.11	0.24	**0.35**	0.22	0.14	**0.38**	**0.48**
**Ala105**	-0.58	-0.45	-0.23	-0.17	-0.30	-0.62	-0.16	-0.19
**Ile110**	-0.05	0.06	0.25	0.26	0.12	-0.23	**0.32**	0.25
**Ile125**	**0.74**	**0.63**	**0.86**	**0.88**	**0.67**	**0.71**	**0.88**	**0.74**
**Met127**	**0.82**	**0.55**	**0.93**	**0.98**	**0.79**	**0.73**	**0.93**	**0.86**
**Ser129**	**0.91**	**0.72**	**1.02**	**1.01**	**0.84**	**0.77**	**1.01**	**0.92**

[a] Relative difference root-mean-square fluctuation (ΔRMSF) was estimated for each ligand-associated QscR protein relative to the apo/unliganded state. Residues showing significant immobility are with ΔRMSF > 0.30 Å cut-off are in bold red color and highlighted.

Table S6. Estimated ΔRMSF[a] values for ligand-CviR *C. violaceum* proteins along the whole MD simulation.

Pocket Residues	HLC	Comp.2	Comp.10	Comp.11	Comp.12	Comp.13	Comp.14	Comp.17	Comp.21
**Leu57**	**0.80**	**0.96**	**0.43**	**0.59**	**0.56**	**0.70**	**0.49**	**0.70**	**0.32**
**Ile69**	-0.72	-0.62	-0.29	-0.49	-0.60	-0.71	-0.51	-0.41	-0.85
**Gln70**	-0.87	-0.75	-0.49	-0.78	-0.62	-0.90	-0.78	-0.62	-1.25
**Arg71**	-0.70	-0.66	-0.43	-0.54	-0.48	-0.78	-0.64	-0.51	-1.16
**Leu72**	0.18	0.28	0.25	0.22	0.13	0.26	0.28	0.18	0.13
**Val75**	-0.14	-0.08	0.23	0.18	-0.40	0.02	0.21	**0.36**	0.12
**Asn77**	-0.72	-0.62	-0.29	-0.49	-0.6	-0.71	-0.51	-0.41	-0.85
**Tyr80**	**0.49**	**0.53**	**0.93**	**0.74**	**0.32**	**0.72**	**0.88**	**1.00**	**0.94**
**Trp84**	0.26	0.08	**0.61**	0.28	0.15	**0.30**	**0.53**	**0.47**	**0.46**
**Leu85**	**0.31**	0.04	**0.57**	0.04	0.02	0.28	**0.40**	**0.36**	**0.63**
**Tyr88**	0.10	**0.64**	0.06	**0.41**	**0.43**	0.05	0.07	0.12	**0.36**
**Met89**	**0.52**	0.10	**0.56**	0.10	0.01	**0.40**	**0.40**	**0.43**	**0.68**
**Ala94**	0.23	-0.51	0.04	-0.06	-0.31	-0.23	0.26	-0.17	0.21
**Gln95**	0.25	-0.41	0.20	-0.07	-0.34	-0.17	0.27	0.12	0.16
**Asp97**	**0.43**	**1.07**	**0.53**	**0.98**	**1.18**	**0.83**	**0.50**	**0.67**	0.29
**Pro98**	-0.37	-0.84	-0.39	-0.82	-0.99	-0.74	-0.50	-0.47	-0.53
**Ile99**	**0.44**	**0.83**	**0.64**	**0.49**	**1.05**	0.28	**0.73**	0.28	**0.62**
**Leu100**	**0.29**	**0.32**	**0.70**	0.29	**1.07**	**0.30**	0.28	0.27	**1.00**
**Arg101**	-0.67	-0.93	-0.50	-0.89	-0.83	-0.81	-0.62	-0.78	-0.79
**Trp111**	**1.25**	**0.96**	**0.66**	**0.36**	**0.60**	**0.68**	**0.75**	**0.87**	**0.61**
**Phe115**	0.21	**0.30**	**0.43**	**0.49**	**0.51**	**0.32**	**0.49**	**0.33**	**0.38**
**Phe126**	-0.70	-1.33	-0.41	-1.07	-1.25	-0.72	-0.97	-0.97	-0.80
**Ala130**	-0.55	-1.09	-0.25	-0.99	-1.12	-0.61	-0.77	-0.46	-0.52
**Met135**	-0.16	-0.59	0.14	-0.59	-0.57	-0.27	-0.36	0.16	0.02
**Thr140**	-0.81	-1.16	-0.52	-1.08	-1.07	-0.84	-1.11	-0.77	-0.71
**Ile153**	**1.38**	**1.59**	**0.78**	**1.16**	**1.08**	**1.23**	**1.24**	**1.17**	**1.31**
**Ser155**	**0.84**	**0.71**	**0.38**	-0.04	**0.65**	**0.66**	-0.21	-0.03	-0.21
**Val250**	**1.31**	0.22	0.08	**2.37**	**0.52**	**0.64**	**1.41**	**0.85**	**0.38**

[a] Relative difference root-mean-square fluctuation (ΔRMSF) was estimated for each ligand-associated CviR protein relative to the apo/unliganded state. Residues showing significant immobility are with ΔRMSF > 0.30 Å cut-off are in bold red color and highlighted.

Table S7. Sequences of the used primers in this study

Target gene	Sequence (5′–3′)	Reference
lasI	**For:** CTACAGCCTGCAGAACGACA<br>**Rev:** ATCTGGGTCTTGGCATTGAG	[1]
lasR	**For:** ACGCTCAAGTGGAAAATTGG<br>**Rev:** GTAGATGGACGGTTCCCAGA	[1]
rhlI	**For:** CTCTCTGAATCGCTGGAAGG<br>**Rev:** GACGTCCTTGAGCAGGTAGG	[2]
rhlR	**For:** AGGAATGACGGAGGCTTTTT<br>**Rev:** CCCGTAGTTCTGCATCTGGT	[1]
pqsA	**For:** TTCTGTTCCGCCTCGATTTC<br>**Rev:** AGTCGTTCAACGCCAGCAC	[1]
pqsR	**For:** AACCTGGAAATCGACCTGTG<br>**Rev:** TGAAATCGTCGAGCAGTACG	[1]
rpoD	**For:** GGGCGAAGAAGGAAATGGTC<br>**Rev:** CAGGTGGCGTAGGTGGAGAAC	[1]
qseC	**For:** GGTACCAAATTGACGCAACGTCTCAG<br>**Rev:** GAATTCGCCCAACTTACTACGGCCTC	[3]
qseE	**For:** GGTACCAGCGACACGTTGAAGCGC<br>**Rev:** GAATTCGCGTGTTTGTCAGATGCAGG	[3]
gyrB	**For:** GTGATCAGCGTCGCCACT<br>**Rev:** GCGCGGTGATCAGCGTC	[4]

References

1. Hegazy, W. A. H.; Khayat, M. T.; Ibrahim, T. S.; Nassar, M. S.; Bakhrebah, M. A.; Abdulaal, W. H.; Alhakamy, N. A.; Bendary, M. M., Repurposing Anti-diabetic Drugs to Cripple Quorum Sensing in Pseudomonas aeruginosa. *Microorganisms* **2020,** 8, (9).
2. Aldawsari, M. F.; Khafagy, E. S.; Saqr, A. A.; Alalaiwe, A.; Abbas, H. A.; Shaldam, M. A.; Hegazy, W. A. H.; Goda, R. M., Tackling Virulence of Pseudomonas aeruginosa by the Natural Furanone Sotolon. *Antibiotics (Basel)* **2021,** 10, (7).
3. Moreira, C. G.; Sperandio, V., Interplay between the QseC and QseE bacterial adrenergic sensor kinases in Salmonella enterica serovar Typhimurium pathogenesis. *Infect Immun* **2012,** 80, (12), 4344-53.
4. Askoura, M.; Hegazy, W. A. H., Ciprofloxacin interferes with Salmonella Typhimurium intracellular survival and host virulence through repression of Salmonella pathogenicity island-2 (SPI-2) genes expression. *Pathog Dis* **2020,** 78, (1).

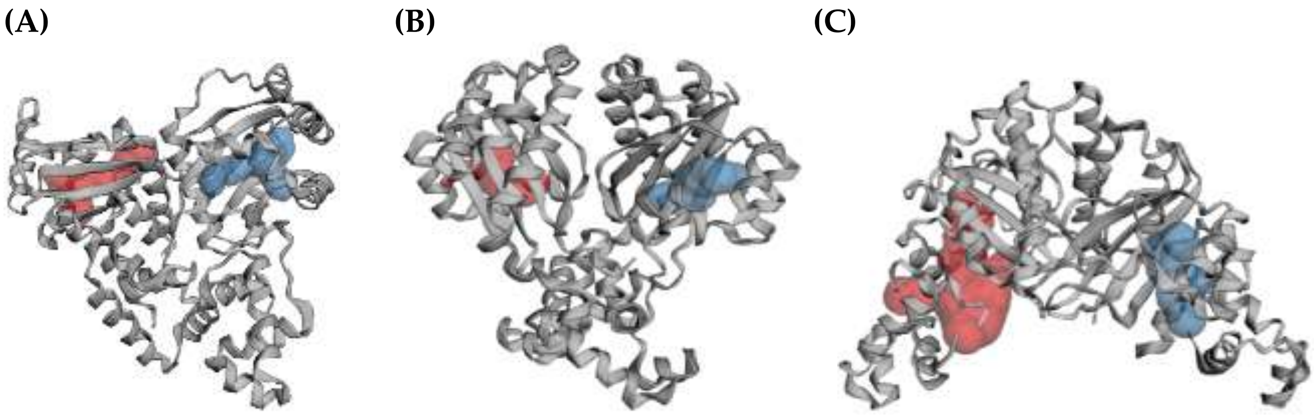

Figure S1. 3D-representation of the binding site topology at the three bacterial LuxR-type quorum-sensing transcription factors. (**A**) TraR *Agrobacterium tumefaciens*; (**B**) QscR *Pseudomonas aeruginosa*; (**C**) CviR *Chromobacterium violaceum*. Putative pockets were calculated via the on-line Computed Atlas of Surface Topography of proteins (CASTp; http://sts.bioe.uic.edu/castp/index.html) using 1.4 Å radius probe, visualized as surface 3D-representation, and colored in different colors (blue and red) for each target protomer. Estimated pocket area and volume were analytically calculated using the Richard's solvent-accessible surface model.

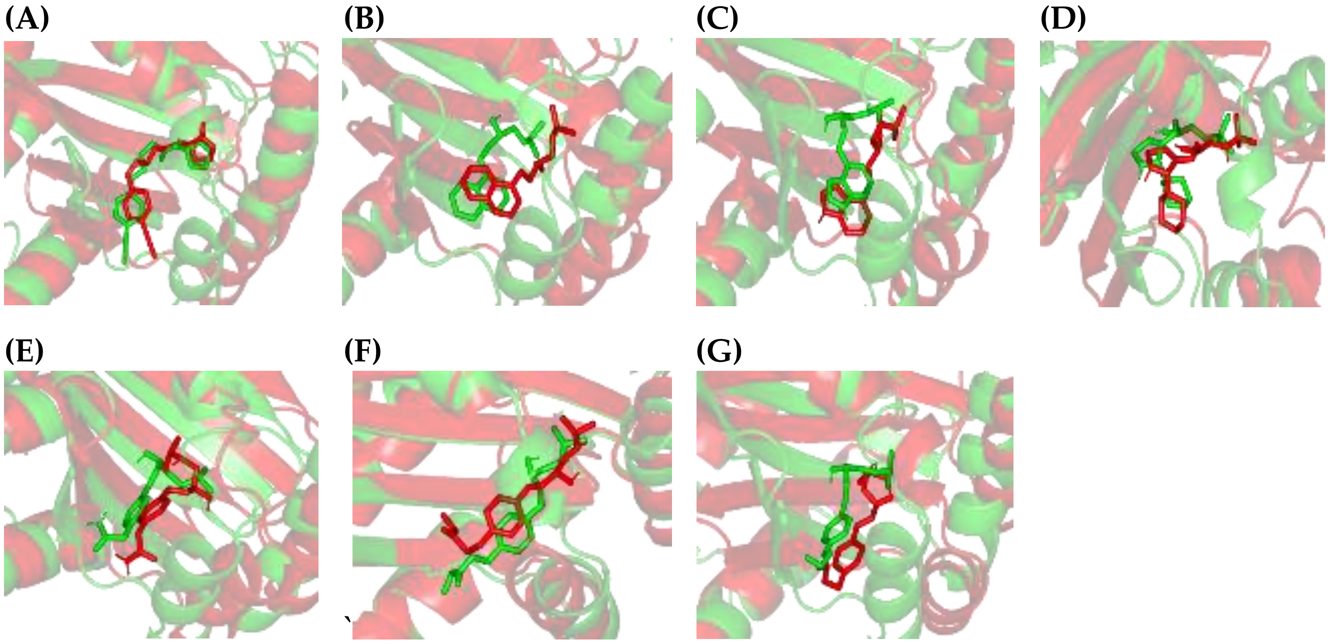

Figure S2. Conformational analysis of simulated ligand-TraR *A. tumefaciens* protein complexes. (**A**) HLC; (**B**) Comp.**1**; (**C**) Comp.**2**; (**D**) Comp.**8**; (**E**) Comp.**10**; (**F**) Comp.**11**; (**G**) Comp.**14**. Overlaid snapshots of the ligand-protein complex at 0 ns and 100 ns of the MD simulation runs. The TraR *A. tumefaciens* proteins are represented in green and red cartoon 3D-representation corresponding to initial and last extracted frames, respectively. Ligands (sticks) are presented in colors corresponding to their respective extracted frames.

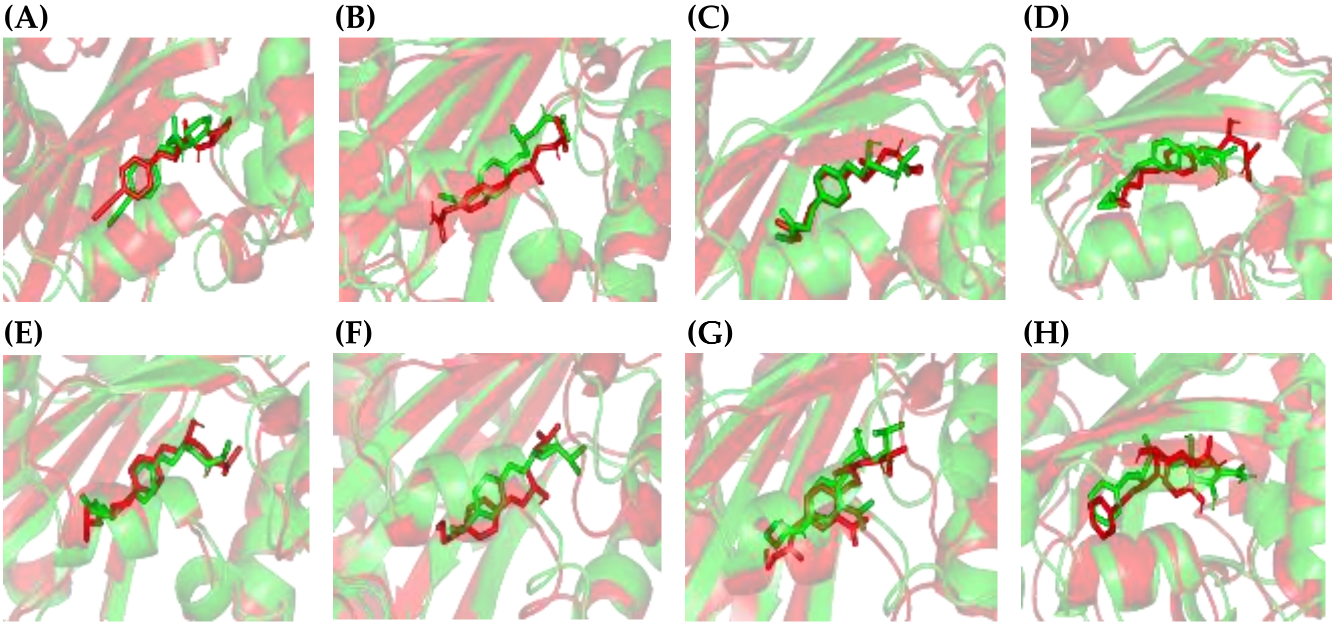

Figure S3. Conformational analysis of simulated ligand-QscR *P. aeruginosa* protein complexes. (**A**) HLC; (**B**) Comp.**10**; (**C**) Comp.**11** (**D**) Comp.**12**; (**E**) Comp.**13**; (**F**) Comp.**14**; (**G**) Comp.**17**; (**H**) Comp.**21**. Overlaid snapshots of the ligand-protein complex at 0 ns and 100 ns of the MD simulation runs. The QscR *P. aeruginosa* proteins are represented in green and red cartoon 3D-representation corresponding to initial and last extracted frames, respectively. Ligands (sticks) are presented in colors corresponding to their respective extracted frames.

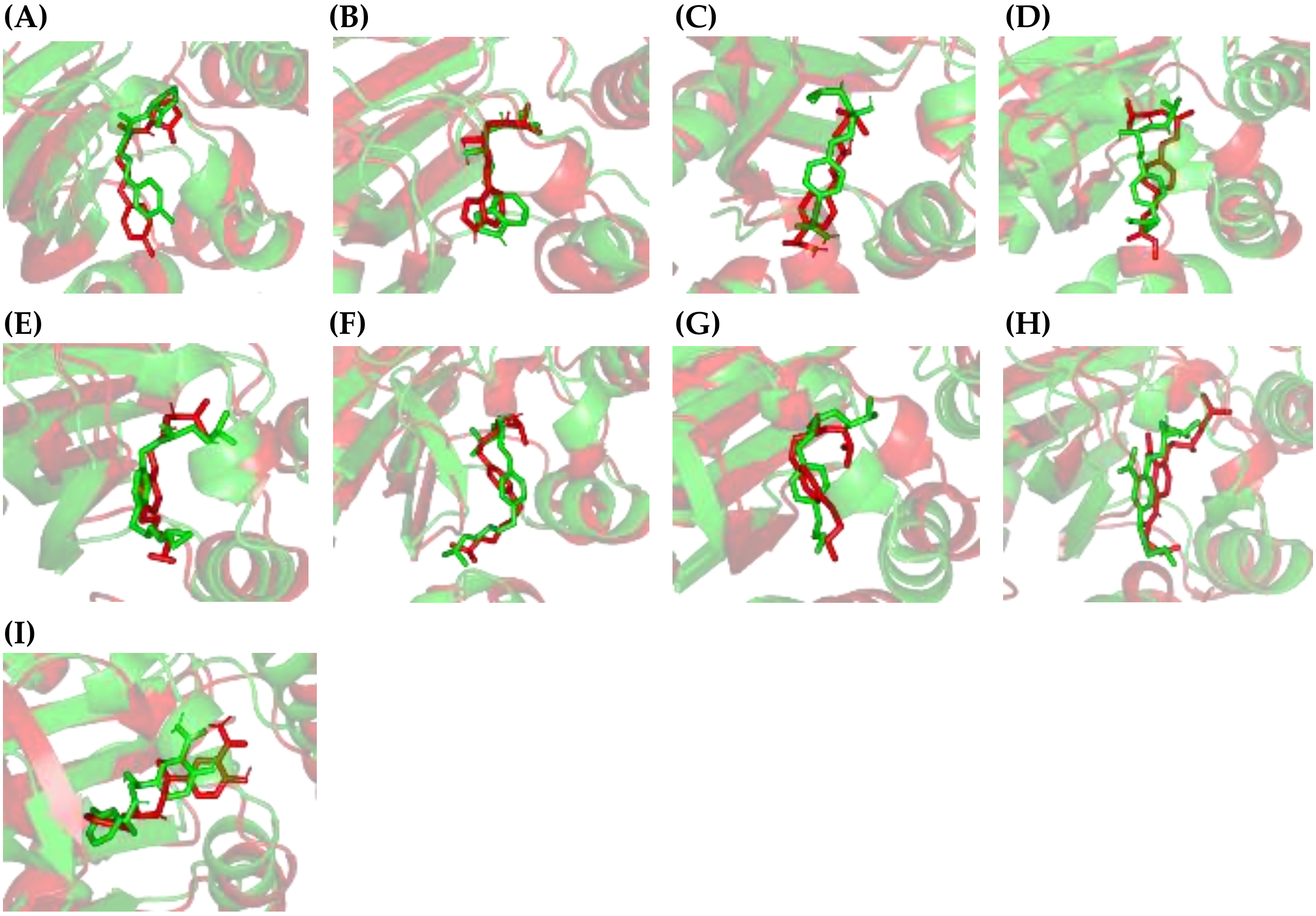

Figure S4. Conformational analysis of simulated ligand-CviR *C. violaceum* protein complexes. (**A**) HLC; (**B**) Comp.**2**; (**C**) Comp.**10** (**D**) Comp.**11**; (**E**) Comp.**12**; (**F**) Comp.**13**; (**G**) Comp.**14**; (**H**) Comp.**17**; (**I**) Comp.**21**. Overlaid snapshots of the ligand-protein complex at 0 ns and 100 ns of the MD simulation runs. The CviR *C. violaceum* proteins are represented in green and red cartoon 3D-representation corresponding to initial and last extracted frames, respectively. Ligands (sticks) are presented in colors corresponding to their respective extracted frames.

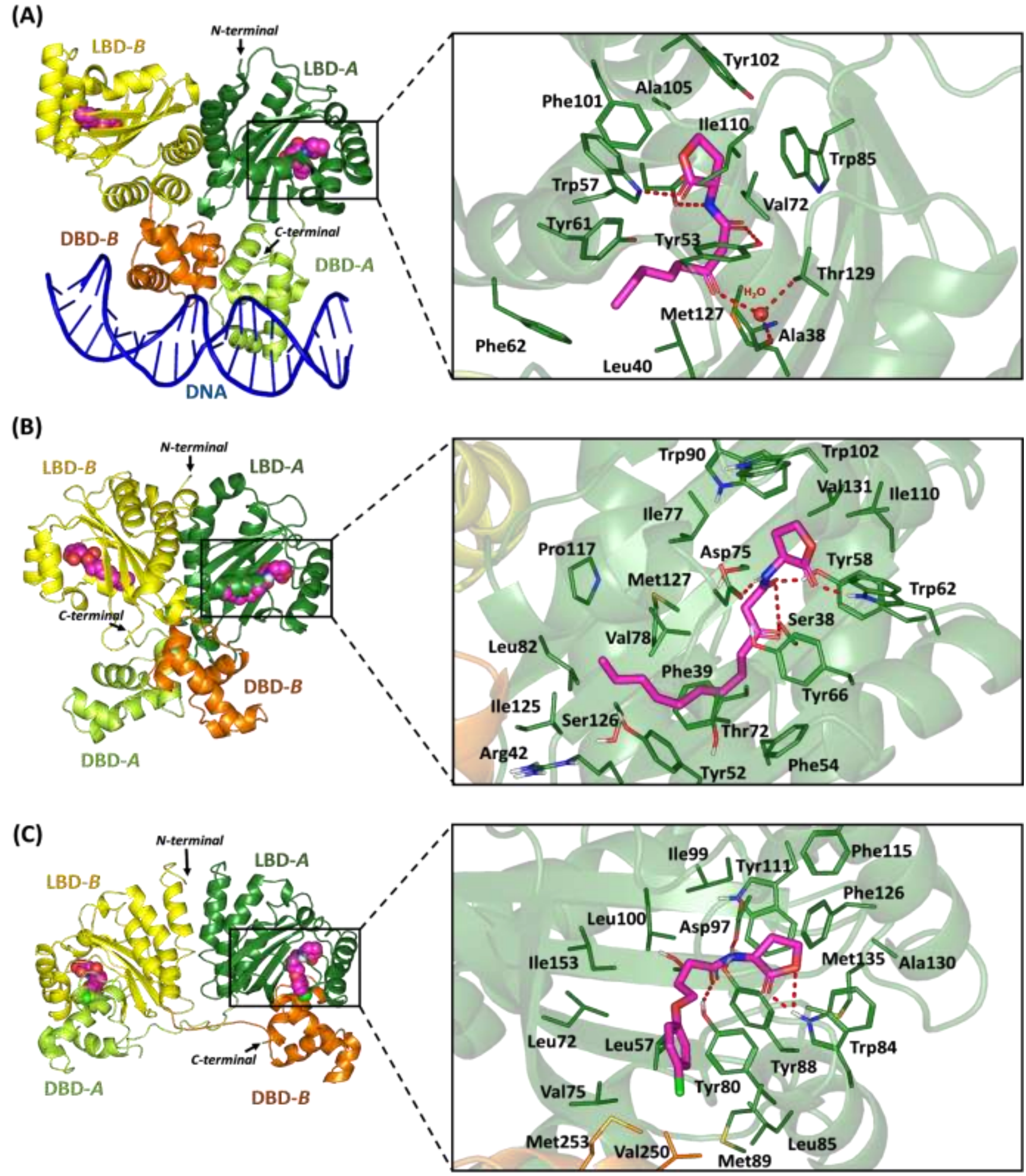

Figure S5. The architecture of three bacterial LuxR-type quorum sensing transcription factors. Left panels are overall cartoon representation of the TraR from *A. tumefaciens* PDB entry: 1L3L (**A**), QscR from *P. aeruginosa* PDB entry: 3SZT (**B**), and CviR from *C. violaceum* PDB entry: 3QP5 (**C**), where each protomer is colored differently in regard to its ligand-binding domain (LBD) and DNA-binding domain (DBD) as light/dark green and dark/light orange for protomer-A and -B, respectively. Ligands are represented as magenta spheres, whereas DNA is presented as blue cartoon only at *A. tumefaciens* TraR crystal structure. Right panels are the overlay of each co-crystallized ligand (magenta sticks) at the binding site of its respective bacterial LuxR-type quorum sensing transcription factor; (**A**) O-C8-HSL, (**B**) O-C12-HSL, and (**C**) HLC. Polar interactions, represented as hydrogen bonds, are illustrated as red dashed-lines and only residues (green lines), located within 4 Å radius of bound ligand, are displayed and labeled with sequence number. Crystallized water molecule bridging the ligand/residue interactions is shown as red sphere.

(A) **(B)** **(C)**

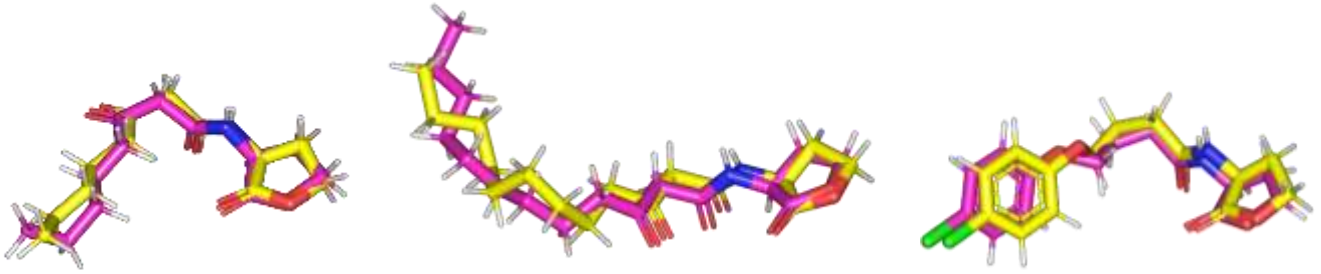

Figure S6. Superimposing the co-crystallized (magenta sticks) and redocked (yellow sticks) ligands. (**A**) TraR *A. tumefaciens*; (**B**) QscR *P. aeruginosa*; (**C**) CviR *C. violaceum* for validating the adopted directed docking protocol.
